# Supplementary material for: Introducing pre-exposure prophylaxis to prevent HIV acquisition among men who have sex with men in Sweden: insights from a mathematical pair formation model
Source: BMJ Open. 2020 Feb 5;10(2):e033852. doi: 10.1136/bmjopen-2019-033852 (PMC7045051; doi:10.1136/bmjopen-2019-033852)
Supplement: Author's manuscript [file bmjopen-2019-033852.draft_revisions.pdf]

BMJ Open

BMJ Open is committed to open peer review. As part of this commitment we make the peer review history of every article we publish publicly available.

When an article is published we post the peer reviewers' comments and the authors' responses online. We also post the versions of the paper that were used during peer review. These are the versions that the peer review comments apply to.

The versions of the paper that follow are the versions that were submitted during the peer review process. They are not the versions of record or the final published versions. They should not be cited or distributed as the published version of this manuscript.

BMJ Open is an open access journal and the full, final, typeset and author-corrected version of record of the manuscript is available on our site with no access controls, subscription charges or pay-per-view fees (http://bmjopen.bmj.com).

If you have any questions on BMJ Open's open peer review process please email info.bmjopen@bmj.com

BMJ Open

Introducing pre-exposure prophylaxis to prevent HIV acquisition among men who have sex with men in Sweden: insights from a mathematical pair-formation model

Journal:	*BMJ Open*
Manuscript ID	bmjopen-2019-033852
Article Type:	Original research
Date Submitted by the Author:	24-Aug-2019

Complete List of Authors:	Hansson, Disa; Stockholms Universitet Matematiska institutionen, mathematics<br>Strömdahl, Susanne; Uppsala University, Department of Medical Sciences; Karolinska Institute, Department of Public Health Sciences<br>Leung, Ka Yin; Stockholm University, Department of mathematics<br>Britton, Tom; Stockholms Universitet Matematiska institutionen, Mathematics

Keywords:	HIV & AIDS < INFECTIOUS DISEASES, Pre-exposure prophylaxis, Mathematical models, Transmission dynamics, Sexual networks

SCHOLARONE™
Manuscripts

I, the Submitting Author has the right to grant and does grant on behalf of all authors of the Work (as defined in the below author licence), an exclusive licence and/or a non-exclusive licence for contributions from authors who are: i) UK Crown employees; ii) where BMJ has agreed a CC-BY licence shall apply, and/or iii) in accordance with the terms applicable for US Federal Government officers or employees acting as part of their official duties; on a worldwide, perpetual, irrevocable, royalty-free basis to BMJ Publishing Group Ltd ("BMJ") its licensees and where the relevant Journal is co-owned by BMJ to the co-owners of the Journal, to publish the Work in this journal and any other BMJ products and to exploit all rights, as set out in our licence.

The Submitting Author accepts and understands that any supply made under these terms is made by BMJ to the Submitting Author unless you are acting as an employee on behalf of your employer or a postgraduate student of an affiliated institution which is paying any applicable article publishing charge ("APC") for Open Access articles. Where the Submitting Author wishes to make the Work available on an Open Access basis (and intends to pay the relevant APC), the terms of reuse of such Open Access shall be governed by a Creative Commons licence – details of these licences and which Creative Commons licence will apply to this Work are set out in our licence referred to above.

Other than as permitted in any relevant BMJ Author's Self Archiving Policies, I confirm this Work has not been accepted for publication elsewhere, is not being considered for publication elsewhere and does not duplicate material already published. I confirm all authors consent to publication of this Work and authorise the granting of this licence.

For peer review only

Introducing pre-exposure prophylaxis to prevent HIV acquisition among men who have sex with men in Sweden: insights from a mathematical pair-formation model

Disa Hansson*[a] Susanne Strömdahl[b] KaYin Leung[a] Tom Britton[a]

Number of words: 3389

For peer review only

* Corresponding author. E-mail address: disa@math.su.se (D. Hansson). Phone number: +46704029803
[a] Department of Mathematics, Stockholm University, SE-106 91 Stockholm, Sweden
[b] Department of Medical Sciences, Section of Infectious Diseases, Uppsala University & Department of Public Health Sciences, Karolinska Institutet

Abstract

Objectives Since 2017, the Public health Agency of Sweden recommend that pre-exposure prophylaxis for HIV (PrEP) should be offered to high-risk individuals, in particular to men who have sex with men (MSM). The objective of this study is to develop a mathematical model investigating the effect of introducing PrEP to MSM in Sweden.

Design A pair-formation model, including steady and casual sex partners, is developed to study the impact of introducing PrEP. Two groups are included in the model: sexually high-active MSM and sexually low-active MSM. Three mixing assumptions between the groups are considered.

Setting A gay-friendly MSM HIV/STI-testing clinic in Stockholm, Sweden. This clinic started offering PrEP to MSM in October 2018.

Participants The model is calibrated according to detailed sexual behaviour data gathered in 2015 among 403 MSM.

Results By targeting sexually high-active MSM, a PrEP coverage of 3.5% of the MSM population (10% of all high-actives) would result in the long-term prevalence to drop considerably (close to 0%). While targeting only low-actives would require a PrEP coverage of 35% for a similar reduction. The main effect of PrEP is the reduced susceptibility, whereas the increased HIV-testing rate (every 3rd month) among PrEP users plays a lesser role.

For peer review only

Conclusions To create a multifaceted picture of the effects of interventions against HIV, we need models that include the different stages of HIV infection and real-world data on detailed sexual behaviour to calibrate the mathematical models. Our findings conclude that targeting HIV high-risk individuals, within HIV risk populations such as MSM, with PrEP programmes could greatly decrease the long-term HIV prevalence in Sweden. Therefore, risk stratification of individuals is of importance in PrEP implementation programmes, to ensure optimising the effect and cost-effectiveness of such programmes.

Strengths and limitations of this study

- Using a mathematical pair-formation model we study the effect of introducing PrEP among MSM in Sweden, a group at high risk of HIV acquisition.
- The model divides the population into sexually high-active MSM and low-active MSM, where high-actives are offered to use PrEP.
- The model is calibrated to detailed sexual behavioural data gathered among the MSM population now being offered PrEP in Sweden.
- Limitations of this study include that the data only makes it possible to include two activity-groups, and that we do not allow for more than one steady sex partner at a time.

For peer review only

INTRODUCTION

In Sweden, the HIV prevalence was estimated to 0.07% in the general population in 2015,[1] whereas the self-reported HIV prevalence among men who have sex with men (MSM) has been estimated between 2% to 6%.[2] Sweden was the first country to report having achieved the UNAIDS/WHO 90-90-90 goal in 2016,[1] with at least 90% of people living with HIV being aware of their HIV status, 90% of HIV diagnosed individuals being on antiretroviral therapy (ART), and with 90% of those on ART being under viral suppression.[3] Viral suppression means achieving continuously undetectable HIV viral load that diminishes onward transmission to close to zero.[4,5]

As a result of ART's effectiveness in viral suppression it can be viewed as an effective preventive measure for further HIV transmission. However, on its own it does not seem to reduce HIV prevalence enough in risk-groups, such as the MSM population, but needs to be combined with additional preventive strategies.[6–8] One such preventive intervention is oral pre-exposure prophylaxis for HIV (PrEP), i.e. that the antiviral drugs tenofovir-emtricitabine are taken by individuals with negative HIV serostatus to prevent HIV acquisition.[9,10] PrEP effectiveness is dependent on adherence to PrEP to ensure that protective concentrations of the drugs are present at exposure to prevent transmission.[11] Two different studies report that PrEP reduces the HIV incidence by 86% among MSM (95% CI 40–98 and 90% CI 64–96 respectively).[9,10] Due to the effectiveness of PrEP, the World Health Organisation (WHO) recommend PrEP to be offered to individuals at substantial risk of HIV acquisition, defined as an HIV incidence of 3 or above per 100 person-years.[12]

For peer review only

The use of PrEP in Sweden was approved in 2016 by the Swedish Medical Products Agency.[13] Since 2017, the Public health Agency of Sweden recommend that PrEP should be offered to high-risk MSM. However, very few clinics started offering PrEP at this time due to logistical and funding concerns. Since June 2018, the New Therapies Council recommend Swedish counties to implement PrEP programmes for MSM and offer subsidised PrEP.[14] The larger gay-friendly sexual health clinics in the major three urban areas of Sweden have since then started to implement PrEP. As of July 2019, approximately 315 individuals have initiated PrEP in Stockholm (personal communication with Dr. FinnFilén responsible for PrEP at Venhälsan, Södersjukhuset).

The objective of this study is to investigate the effect of introducing PrEP to MSM in Sweden. First, we incorporate the use of PrEP into pair-formation models developed to study HIV transmission.[15] This model separates individuals depending on sexual activity-degree, high-active or low-active. The model is then fitted to sexual behaviour data from a gay-friendly HIV/STI-testing clinic in Stockholm, Sweden. Finally, the effect of PrEP on HIV transmission is studied by risk-stratifying MSM for PrEP, to explore the level of PrEP coverage needed to substantially reduce the long-term HIV prevalence.

METHODS

To study the introduction of PrEP, we develop a pair-formation model that includes steady (long-term) partnerships and casual (one-off/occasional) sex partners. We categorise individuals as sexually high-active or low-active, with different sex partner mixing patterns, different HIV-diagnosis rates, and allowing for high-actives to use PrEP. We incorporate two stages of HIV infectiousness: the early acute (primary) stage and the subsequent chronic

For peer review only

(asymptomatic) stage.[16] The model is then calibrated to detailed sexual behaviour data and the observed HIV prevalence. We earlier developed a model to disentangle the roles of casual and steady partnerships on HIV transmission, which we further elaborate in this work.[15] We begin by describing the model for sexual contacts and continue with how transmission of HIV-infection is modelled. Then we apply sexual behaviour data gathered among MSM. The full description of the model can be found in the Supplementary material S1.

Dynamic pair-formation model

Consider a sexually active same-sex population where new individuals enter the sexually active population without having a steady sex partner. Individuals can have at most one steady partner at a time, which can end by separation or death of either partner. Individuals can also have casual sex partners during steady partnerships as well as during periods without steady partnerships; the rate at which this occurs depends on the steady partnership status of the individuals under consideration. Based on data, we allow singles to have a higher rate of casual sex than individuals in a steady partnership. Not only partnership status affects the rate of finding new casual sex partners, we additionally allow for individuals to be either high-active or low-active regarding the frequency of having casual sex partners.

Letting the rate of finding a new casual sex partner depend on the partnership status and the activity-degree of both potential members in the sexual act, yields 16 different casual sex partnership combinations. We let α_{ij}^{rq} denote the rate at which an individual with activity-degree r and i steady partners try to find a casual sex partner with activity-degree q and j steady partners, where $r,q \in \{h = \text{high},\ l = \text{low}\}$ and $i,j \in \{0,1\}$. These rates play an

For peer review only

important role in our modelling and are described in detail in the Supplementary material S1 and S2.

Casual sex and mixing patterns

Creating the groups high-active and low-active makes it necessary to formulate mixing between the groups. Three activity-degree mixing assumptions are considered: proportionate mixing, complete assortativity, and mixing fitted to responses of a proxy question. Common to all three models is that high-actives have casual contacts at a fixed rate being larger than that of low-actives. Proportionate mixing implies that an individual chooses a casual partner at random among the potential casual sex attempts in the population, i.e. the probability of having a high-active casual partner is the same for low-active and high-active individuals. Complete assortativity means that high-actives only have high-active casual partners and low-actives only have low-active casual partners.

We estimate the assortativity according to the testing-clinic participants answer to one question on partners' sexual activity, as described in Supplementary material S3.5. This question is referred to as a proxy question for partners activity-degree. The proxy for partners activity-degree and knowing whether an individual is high-active or low-active, specifies the amount of assortativity in the data. The estimated assortativity can take values between 0 and 1, where 0 corresponds to proportionate mixing and 1 to complete assortativity.

Since we do not know whether or not participants' casual sex partners are in steady partnerships, the mixing between singles and individuals in a partnership is assumed to be random (proportionate mixing).

For peer review only

Model of infection

To model the spread of infection we use a compartmental model, much like the SIR model (Susceptible → Infectious → Recovered),[17] but with two stages of infectiousness: acute followed by chronic. The probability of transmission of HIV depends on the stage of infection when no antiretroviral treatment is used.[18] In the early acute stage the probability of transmission is much higher due to higher viral load than in the chronic stage. The probability of transmission in one unprotected sex act (in our case anal intercourse) is denoted p_A when in the acute stage and p_C when in the chronic stage. The model allows for different levels of condom use with steady and casual sex partners, and it is incorporated by reducing the transmission probability accordingly. The time until diagnosis and the beginning of ART depends on the degree of sexual activity. Further, individuals on ART are assumed to be virally suppressed and thereby to no longer transmit infection. The compartmental model therefore divides the population into susceptible, infectious in the acute stage, infectious in the chronic stage, and being on ART-treatment.

The aim with defining this model is to introduce the possibility for high-actives to take PrEP, which, when taken correctly, decreases the probability of getting infected with HIV by approximately 86%.[9,10] Moreover, individuals accepting PrEP need to test themselves every third month. The model is illustrated in Supplementary material Figure S1.1.

For peer review only

Data and calibration

The data was gathered at a gay-friendly HIV/STI-testing clinic (Venhälsan) in Stockholm, Sweden, during 2015.[19] MSM visiting testing-clinics might be more sexually active than other MSM, e.g. sexually inactive or MSM with one sex partner might not visit testing-clinics as often. 403 MSM participants answered a structured timeline follow-back questionnaire and reported their total number of sex partners during the last 12 months. Detailed sexual behaviour data was collected on participants' last ten sex partners during the last 12 months, including: type of sex partner (casual or steady); frequency of sex acts; condom use with each partner; the duration of each sexual relationship; and the answer to the proxy question on partners' sexual activity-degree. All 403 participants were included in the study by Hansson et al.[15] However, inclusion in this study requires that participants have reported their total number of sex partners during a year to determine their activity-degree. Moreover, for a partner to be included, the proxy question regarding the partner's sexual activity-degree must be answered. Of the 403 participants, four participants reported having zero sex partners the previous 12 months and 28 participants did not answer that particular question. Of the remaining 371 participants, detailed information on 1991 different sex partners (510 steady and 1481 casual) were given. We have an answer to the proxy question for 1424 of the 1481 casual sex partners. When removing the 57 casual sex partners with no answer to the proxy question, 368 participants (and 1903 partners) remain and was included in the analysis presented here.

The participants demonstrate a considerable difference in yearly number of sexual partners, with a range from 1 to 250 sexual partners. We choose the mean (15) as our cut-off for

For peer review only

defining high-active individuals, resulting in that 33.7% are high-actives. The mean number of sex partners for high-actives is 33.2 (sd=32) and for low-actives it is 6.0 (sd=3.2).

Partnership and epidemic parameters

The parameters and their values used in the analysis are given in Table 1. Some parameters are estimated from the testing-clinic data (part 1 - 4 in Table 1), some parameters are varied (part 2 in Table 1), and some are taken from the available literature (part 5 in Table 1). In the analysis, the standard errors of all estimates from the testing-clinic data can be included to obtain a 95% credibility interval (using Monte Carlo simulation) of the prevalence estimated from the model (see Table S3.2).

The mean times to ART, denoted γ_h for a high-active and γ_l for a low-active, are calibrated to fit the observed prevalence. From the data we estimate that $\gamma_h = 2.35\gamma_l$ (Supplementary material S3.3), this relationship will be kept throughout the analysis, such that only one of the parameters need to vary.

Estimated rates of meeting a new casual sex partner

Table 1 shows the estimated rates of meeting a new casual sex partner. In the third part of Table 1 we have not utilised the proxy question on partners activity-degree, these estimates are enough when assuming proportionate mixing or complete assortativity. The proxy question is used to get the estimates in the fourth part of Table 1. From this table and a given choice of mixing pattern, one can estimate the rates of looking for a new casual sex partner α_{ij}^{rq}. The final values of α_{ij}^{rq} can be found in the Supplementary material Table S3.5.

For peer review only

Table 1: *Estimates of partnership and epidemic parameters. Abbreviations used: AI - anal intercourse, URAI - unprotected receptive anal intercourse, UIAI - unprotected insertive anal intercourse.*

**1. Partnership parameters**			
**Parameter**	**Value**	**Definition**	**Source**
$1/(\sigma + 2\mu)$	271.5 days	Mean duration steady partnership	MSM data
$1/\rho P_0$	152.8 days	Mean time being single	MSM data
$1/\lambda$	12.3 days	Mean time between AI within steady partnership	MSM data
P_0	0.360	Fraction without a steady partner	MSM data
P_1	0.640	Fraction with a steady partner	MSM data
π_h	0.337	Fraction high-actives	MSM data
π_l	0.663	Fraction low-actives	MSM data
**2. Parameters for condom use, PrEP, and time to treatment**			**Source**
q_s	54.1%	Mean condom use steady partner	MSM data
q_c	62.9%	Mean condom use casual partner	MSM data
ξ		Rate for a high-active to start taking PrEP (calibrated to achieve different % PrEP coverage)	Varied
		**Mean time from infection to successful antiretroviral treatment for a**	
$1/\gamma_P$	0.25 years	high-active on PrEP	[20]
$1/\gamma_h$	1.5 – 3 years	high-active not on PrEP	Varied
$1/\gamma_l$	3.5 – 7 years	low-active	$1/\gamma_l = 2.35\ /\gamma_h$
**3. Casual sex partner parameters from data not using proxy**			**Source**
		**Mean time until new casual sex partner for a**	
$1/\alpha_0^h$	10.7 days	high-active when single	MSM data
$1/\alpha_1^h$	12.5 days	high-active when in partnership	MSM data
$1/\alpha_0^l$	66.5 days	low-active when single	MSM data
$1/\alpha_1^h$	97.9 days	low-active when in partnership	MSM data
**4. Casual sex partner parameters from data using proxy**			**Source**
		**Mean time until new casual partner for a high-active with a**	
$1/\alpha_0^{hh}$	14.1 days	high-active when single	MSM data
$1/\alpha_0^{hl}$	43.7 days	low-active when single	MSM data
$1/\alpha_1^{hh}$	15.4 days	high-active when in partnership	MSM data
$1/\alpha_1^{hl}$	66.4 days	low-active when in partnership	MSM data
		**Mean time until new casual partner for a low-active with a**	
$1/\alpha_0^{lh}$	109.3 days	high-active when single	MSM data
$1/\alpha_0^{ll}$	169.6 days	low-active when single	MSM data
$1/\alpha_1^{lh}$	136.1 days	high-active when in partnership	MSM data
$1/\alpha_1^{ll}$	348.9 days	low-active when in partnership	MSM data
**5. Parameters from the literature**			
**Parameter**	**Value**	**Definition**	**Source**
	70%	Condom efficiency	[21]
$1/\delta_a$	0.24 years	Mean time in acute infection stage	[18]
$1/\mu$	60 years	Sexually active life-span	[22,23]
		**Per-act transmission probability**	
	0.1835	Acute stage URAI	[16]
	0.0138	Chronic stage URAI	[16]
	1.48%	Overall URAI	[24]
	0.62%	Overall UIAI	[24]
	2.39	Of URAI in comparison to UIAI	[24]
p_A	0.1301	Acute stage combined URAI-UIAI	[16,24]
p_C	0.0098	Chronic stage combined URAI-UIAI	[16,24]

For peer review only

Patient and public involvement

There was no patient and/or public involvement in the planning of this study.

ANALYSIS AND RESULTS

The assortativity regarding activity-degree is measured to 0.14, meaning that the studied population choose casual partners with a moderate assortativity. The HIV prevalence among the 368 participants is 5%, which is in line with national levels among MSM,[2] and this prevalence will be used as a baseline when studying the effect of PrEP. Specifically, the baseline model is the model where no one uses PrEP ($\xi = 0$) and that is calibrated to achieve a 5% equilibrium prevalence. The only parameter not being able to be estimated from the HIV/STI-testing clinic data, or that can be taken from the literature, is the mean time to successful ART-treatment. Hence, to calibrate the model to the observed 5% prevalence, we find the mean time to treatment that corresponds to this prevalence. To study the effect of PrEP, we use the same parameter set-ups as for the baseline model but additionally allow for sexually high-actives to use PrEP ($\xi > 0$), and then find the new equilibrium prevalence. We use 'long-term prevalence' and 'equilibrium prevalence' interchangeably. We begin by presenting the results of the model where PrEP has not yet been introduced, then we move to the model where high-actives are offered PrEP.

Prior to the introduction of PrEP

For peer review only

In dividing the population into two activity-degrees and using fitted assortativity, we find that the prevalence of 5% (95% CRI 2.3-7.6%) is obtained when the mean time to ART is $\gamma_h^{-1} = 1.77$ years for high-actives, (4.15 years for low-actives). In doing this calibration to the observed 5% prevalence, the estimated percent of individuals with positive HIV serostatus that are on ART-treatment is 95.8%.

Disregarding the proxy question, we would not know how the population mix regarding activity-degree. We could then use the other two mixing patterns. Assuming proportionate mixing, the prevalence of 5% (95% CRI 2.1-7.8%) is obtained when $\gamma_h^{-1} = 1.79$ years. This set-up yields that the estimated percent of individuals with HIV that are on ART-treatment is 95.7%. For complete assortativity, the prevalence of 5% (95% CRI 2.7-7.0%) is obtained when $\gamma_h^{-1} = 1.63$ years, and then the estimated percent of individuals with HIV that are on ART-treatment is 96.4%. Figure 1a) depicts the prevalence for varied values of the mean time to ART-treatment and Figure 1b) shows the 95% credibility intervals.

[INSERT FIGURE 1 HERE]

Using the fitted assortativity, a prevalence of 5% was found when the mean time to ART was 1.77 years for high-actives, while the same time to ART for the proportionate mixing assumption yields a prevalence of 4.6% (95% CRI 1.7-7.3%), and complete assortativity yields a prevalence of 6.9% (95% CRI 4.6-8.8%). This shows that higher assortativity regarding activity-degree leads to higher prevalence and easier allows for HIV being endemic. With increased assortativity, the allocation of the infected individuals becomes different, as seen in Table 2, with more high-actives being infected. Interesting to note, from Figure 1a, is that the difference between the mixing assumption decreases with an increased time to ART.

For peer review only

Additionally, from Table 2 we note that approximately 35% of HIV transmissions occur within a steady partnership.

Table 2: For the three mixing assumptions, we show the estimated mean time to ART corresponding to a prevalence of 5%. For this prevalence and for each of the three mixing assumptions, we also show the route of transmissions and HIV prevalence for the two respective activity-degree groups. The shown values for the time to ART-treatment are for high-active individuals, the time to ART-treatment for low-actives is 2.35 times larger. For the allocation of the 5% infected we show the percentage of high-actives and low-actives, respectively, that are HIV-positive.

	**Overall HIV prevalence of 5%**		
	Prop. mixing	Fitted Assort.	Compl. Assort.
**Time to ART (years)**	1.79	1.77	1.63
**Route of transmission**			
Steady partner	35%	35%	32%
Casual sex when in partnership	38%	39%	41%
Casual sex when single	26%	26%	27%
**HIV prevalence in the group**			
High-actives	9.05%	9.23%	10.79%
Low-actives	2.94%	2.85%	2.06%

Effect of introducing PrEP

We now present the effect of introducing PrEP. This is done by starting at a prevalence of 5% and then increasing the PrEP coverage. That is, we use the parameter values from the model without PrEP that achieved a 5% equilibrium prevalence, but now allow high-actives to take PrEP and find the new equilibrium prevalence. We stress that the results are the long-term effect of certain levels of PrEP coverages, even with no more infections it will naturally take a long time for the HIV prevalence to reach 0%.

For peer review only

If the population is not divided according to activity-degree, the PrEP coverage would need to be 5.2% of the total population to reduce the long-term prevalence from the observed 5% to close to 0%. Dividing the population according to activity-degree and only targeting high-actives for PrEP, Figure 2a shows the combined effect of PrEP and an increased testing rate: reaching a coverage of 1% of the population (3.0% of high-actives) will reduce the long-term prevalence from 5% to 3.6%; reaching a coverage of 3.5% of the population (10.4% of high-actives) will reduce the long-term prevalence to 0%.

[INSERT FIGURE 2 HERE]

Being able to target risk groups for PrEP makes a big difference: targeting low-actives instead would result in a needed PrEP coverage of 34.4% of the population to reach an equilibrium prevalence close to 0% (Figure S4.1).

To ascertain the respective effects of PrEP, the decreased susceptibility by 86% and the more frequent HIV-testing rate (every third month), we do two additional analyses. If being on PrEP is not combined with an increased testing rate, but only a reduced susceptibility, reaching a coverage of 3.5% of the population will reduce the prevalence to 0.5% (Figure 2b). If being on PrEP does not give any reduced susceptibility, but only an increased testing rate, reaching a coverage of 3.5% of the population will reduce the prevalence to 1.9%.

For results concerning the more short-term effect of PrEP, see Supplementary material S4.7.

DISCUSSION

Our results suggest that a PrEP coverage of at least 3.5% of the MSM population, when sexually high-actives are targeted, is needed for the long-term prevalence of HIV to drop to

For peer review only

close to 0% among MSM in Sweden. This can be compared to a 34.4% PrEP coverage needed if only low-active MSM were targeted for PrEP. These results emphasise the need for risk stratification among MSM, to ensure that those in need of PrEP receive the intervention. To reach high-risk MSM, out-reach programmes and peer education programmes have been found to be effective, scale up of these may increase the effect of PrEP implementation on the HIV epidemic among MSM in Sweden.[25]

We find that the greatest effect of the combined PrEP intervention follows from the decreased susceptibility to HIV, not the increased HIV-testing rate. This result would be hidden in a model not taking the different stages of infection into account (Supplementary material S4.2). Hence, to make a correct assessment of a PrEP programme's effect, the complexities of HIV transmission, the different stages of infection, need to be accounted for.

The benefit of targeting high-risk individuals for PrEP has been found by other studies.[8,26–29] Our analyses adds to these findings by including additional parameters. Punyacharoensin et al.[8] investigate the effect of different HIV interventions, including PrEP, among MSM in the UK. They define low-actives as MSM with one or fewer new sexual partners a year, while our definition of high-active MSM (at least 15 partners a year) is to address a group with very high HIV risk. Secondly, they address mixing through a different method using an odds ratio among male heterosexuals, while we use data from the MSM population under study. Rozhnova et al.[29] use four risk-groups, however, they do not estimate mixing between the groups but assume intermediate mixing.

Our model has four strengths worth mentioning. First, our model design is strengthened by that it is calibrated to fit detailed data of MSM who visited an STI/HIV-testing clinic in Sweden. For example, when the model is calibrated to data and the observed 5% HIV prevalence, the

For peer review only

estimated percentage of individuals with HIV that are on ART-treatment, 95.8%, are very close to the observed value of 95.1%.[1] In addition, the very same clinic where the data was gathered is the largest implementer of PrEP in Sweden, prescribing PrEP to 315 MSM since October 2018. Secondly, we can measure the assortativity with respect to activity-degree of the study participants. The mixing between high-actives and low-actives is estimated to be more assortative than proportionate mixing (0.14 vs 0), and by using the estimated assortativity we get more reliable results with narrower 95% CRIs than for proportionate mixing (Figure S4.3). For realistic mean times to ART, the mixing assumption has an impact on the estimated prevalence, making it an important factor to include. Thirdly, an important model choice is to include steady partnerships, not only casual contacts, since HIV transmission occurs to a large extent within a steady partnership (Table 2). Finally, the result concerning PrEP coverage is robust to variations in the parameter set-ups (Supplementary material S4.6).

Our model includes limitations. First, the proxy question used to fit the assortativity can only define two activity-degree groups and not more. The real-life scenario is probably more heterogeneous than accounted for in our model; even with a high PrEP coverage, the prevalence would likely stay above 0% due to some sub-groups of MSM taking larger HIV risks than the high-actives within our model. Additionally, some individuals are probably even more low-active (such as MSM in monogamous steady partnerships) than we allow for. Secondly, many possible changes in sexual behaviour are not included. Our model assumes no change in sexual behaviour when being on ART and we do not assume any increased HIV-testing rate for a sex partner to someone living with HIV. Individuals on PrEP are assumed to stay on PrEP, except if they get diagnosed with HIV and are put on ART-treatment. Moreover, individuals are assumed to belong to one of the sexual activity-groups during their whole life.

For peer review only

Thirdly, the data is collected among a convenience sample of MSM visiting an HIV/STI-testing clinic, thereby it is not representative of all MSM in Sweden. Fourthly, the model does not consider imperfect PrEP adherence. However, in Supplementary material S4.4 we consider different values of PrEP effectiveness. Finally, our model does not incorporate concurrent steady partnerships. This is a common assumption for compartmental models,[8,15,30] and inclusion would possibly strengthen the model. However, our model does consider casual sex partners concurrent to steady partners.

In future work, risk compensation could be studied more thoroughly, e.g. changed behaviour of individuals on PrEP. Other risk behaviours for HIV than sexual activity-degree could be considered to define the risk-group offered PrEP, such as taking part in group sex, consistent drug use, and transactional sex. Another possible extension is to stratify our model by age, letting also activity-degree vary between the age groups to capture that certain age groups could be more sexually active.

We conclude by stating the result emerged from the heterogeneous activity-degrees: heterogeneity in sexual activity does increase the prevalence, however, it also makes targeted interventions much more effective.

Acknowledgements The authors thank the study participants for their contribution and acknowledge the work of staff at Venhälsan STI/HIV clinic in Stockholm, Sweden.

Contributions DH, SS, KYL, and TB conceived the study; SS designed and managed the gathering of data; DH, TB and KYL defined the model. DH, SS and TB drafted the manuscript. All authors approved the manuscript before submission for publication.

For peer review only

Funding The data collection was financially supported by the Swedish State Grant for HIV/STI prevention (HSN 1309-1016) and an unrestricted grant from Gilead Sciences Nordic Fellowship Programme 2014. The funders had no role in study design, data collection, analysis or write up of results. TB and KYL are supported by The Swedish Research Council, grant no. 2015-05015.

Competing interests None to declare.

Patient consent for publication Written informed consent was provided by all participants.

Ethics approval Ethical approval for the study data collection was obtained from the Regional Ethical Committee in Stockholm, Sweden (Dnr. 2014/1729-31/5).

Data availability statement Extra data is available by contacting SS.

For peer review only

REFERENCES

1 Gisslén M, Svedhem V, Lindborg L, *et al.* Sweden, the first country to achieve the Joint United Nations Programme on HIV/AIDS (UNAIDS)/World Health Organization (WHO) 90-90-90 continuum of HIV care targets. *HIV Med* 2017;**18**:305–7. doi:10.1111/hiv.12431

2 The Public Health Agency of Sweden. MSM2013, A study about sex, HIV, and health among men who have sex with men in Sweden. https://www.folkhalsomyndigheten.se/contentassets/4133e13e29204433a459d5ebbf0fcebe/msm2013-studie-sex-hiv-halsa-15139.pdf

3 Ministry of Health and Social A airs GO ces of S. Nationell strategi mot hiv/aids och

vissa andra smittsamma sjukdomar. 2017.https://www.regeringen.se/informationsmaterial/2017/12/nationell-strategi-mot-hivaids-och-andra-smittsamma-sjukdomar/ (accessed 6 May 2018).

4 Cohen MS, Chen YQ, McCauley M, *et al.* Prevention of HIV-1 Infection with Early Antiretroviral Therapy. *N Engl J Med* 2011;**365**:493–505. doi:10.1056/NEJMoa1105243

5 HIV TL. U=U taking off in 2017. *Lancet HIV* 2017;**4**:e475. doi:10.1016/S2352-3018(17)30183-2

6 Cremin I, Alsallaq R, Dybul M, *et al.* The new role of antiretrovirals in combination HIV prevention: a mathematical modelling analysis. *AIDS* 2013;**27**.https://journals.lww.com/aidsonline/Fulltext/2013/01280/The_new_role_of_antiretrovirals_in_combination_HIV.15.aspx

7 Beyrer C, Sullivan P, Sanchez J, *et al.* The increase in global HIV epidemics in MSM. *AIDS* 2013;**27**.https://journals.lww.com/aidsonline/Fulltext/2013/11130/The_increase_in_global_HIV_epidemics_in_MSM.1.aspx

8 Punyacharoensin N, Edmunds WJ, De Angelis D, *et al.* Effect of pre-exposure prophylaxis and combination HIV prevention for men who have sex with men in the UK: a mathematical modelling study. *Lancet HIV* 2016;**3**:e94–104. doi:10.1016/S2352-3018(15)00056-9

9 Molina J-M, Capitant C, Spire B, *et al.* On-Demand Preexposure Prophylaxis in Men at High Risk for HIV-1 Infection. *N Engl J Med* 2015;**373**:2237–46.

For peer review only

doi:10.1056/NEJMoa1506273

10 McCormack S, Dunn DT, Desai M, *et al.* Pre-exposure prophylaxis to prevent the acquisition of HIV-1 infection (PROUD): effectiveness results from the pilot phase of a pragmatic open-label randomised trial. *Lancet (London, England)* 2016;**387**:53–60. doi:10.1016/S0140-6736(15)00056-2

11 Sidebottom D, Ekström AM, Strömdahl S. A systematic review of adherence to oral pre-exposure prophylaxis for HIV - how can we improve uptake and adherence? *BMC Infect Dis* 2018;**18**:581. doi:10.1186/s12879-018-3463-4

12 World Health Organization. *Consolidated guidelines on the use of antiretroviral drugs for treating and preventing HIV infection. Recommendations for a public health approach - Second edition*. 2016. https://www.who.int/hiv/pub/arv/arv-2016/en/

13 The Public Health Agency of Sweden. Preexpositionsprofylax för att minska risken för infektion med hiv – en kunskapsöversikt. 2017.https://www.folkhalsomyndigheten.se/publicerat-material/publikationsarkiv/p/preexpositionsprofylax-for-att-minska-risken-for-infektion-med-hiv-en-kunskapsoversikt/ (accessed 13 Sep 2018).

14 NT-rådet. Emtricitabin tenofovirdisoproxil som preexpositionsprofylax (PrEP) för att minska risken för hiv-infektion. https://janusinfo.se/download/18.11b119de1639e38ca5fb2e4a/1535626547383/Emtricitabin-tenofovirdisoproxil-(PrEP)-180611.pdf

15 Hansson D, Leung KY, Britton T, *et al.* A dynamic network model to disentangle the roles of steady and casual partners for HIV transmission among MSM. *Epidemics*

For peer review only

Published Online First: 2019. doi:https://doi.org/10.1016/j.epidem.2019.02.001

16 Leynaert B, M Downs A, de Vincenzi I. *Heterosexual transmission of human immunodeficiency virus: variability of infectivity throughout the course of infection. European Study Group on Heterosexual Transmission of HIV*. 1998. doi:10.1093/oxfordjournals.aje.a009564

17 Ogilvy KW, G. MA, Thomas WG. A contribution to the mathematical theory of epidemics. *Proc R Soc London Ser A, Contain Pap a Math Phys Character* 1927;**115**:700–21. doi:10.1098/rspa.1927.0118

18 Hollingsworth TD, Anderson RM, Fraser C. HIV-1 Transmission, by Stage of Infection. *J Infect Dis* 2008;**198**:687–93.http://dx.doi.org/10.1086/590501

19 Sidebottom D, Hansson D, Liljeros F, *et al.* Comprehensive measurement of HIV/STI risk behaviour among MSM using a tablet-based timeline follow-back tool. *Submitted*

20 (RAV) TSRG for AT. HIV preexpositionsprofylax (PrEP) – Rekommendation om praktisk handläggning 2017. https://www.sls.se/globalassets/rav/rekommendationer/rav_hiv_prep_2017_v170824.pdf

21 Smith DK, Herbst JH, Zhang X, *et al.* Condom Effectiveness for HIV Prevention by Consistency of Use Among Men Who Have Sex With Men in the United States. *JAIDS J Acquir Immune Defic Syndr* 2015;**68**.https://journals.lww.com/jaids/Fulltext/2015/03010/Condom_Effectiveness_for_HIV_Prevention_by.14.aspx

22 The Public Health Agency of Sweden. Sexualitet och hälsa bland unga i Sverige –

For peer review only

UngKAB15 – en studie om kunskap, attityder och beteende bland unga 16–29 år. 2017. https://www.folkhalsomyndigheten.se/publicerat-material/publikationsarkiv/s/sexualitet-och-halsa-bland-unga-i-sverige-ungkab15-en-studie-om-kunskap-attityder-och-beteende-bland-unga-1629-ar/

23 World Bank. Life expectancy at birth 2015. https://data.worldbank.org/indicator/SP.DYN.LE00.MA.IN?locations=SE (accessed 12 Jul 2017).

24 Jin F, Jansson J, Law M, *et al.* Per-contact probability of HIV transmission in homosexual men in Sydney in the era of HAART. *AIDS* 2010;**24**.https://journals.lww.com/aidsonline/Fulltext/2010/03270/Per_contact_probability_of_HIV_transmission_in.14.aspx

25 Strömdahl S, Hoijer J, Eriksen J. Uptake of peer-led venue-based HIV testing sites in Sweden aimed at men who have sex with men (MSM) and trans persons: a cross-sectional survey. *Sex Transm Infect* 2019;:sextrans-2019-054007. doi:10.1136/sextrans-2019-054007

26 Pretorius C, Stover J, Bollinger L, *et al.* Evaluating the cost-effectiveness of pre-exposure prophylaxis (PrEP) and its impact on HIV-1 transmission in South Africa. *PLoS One* 2010;**5**:e13646–e13646. doi:10.1371/journal.pone.0013646

27 Abbas UL, Anderson RM, Mellors JW. Potential Impact of Antiretroviral Chemoprophylaxis on HIV-1 Transmission in Resource-Limited Settings. *PLoS One* 2007;**2**:e875.https://doi.org/10.1371/journal.pone.0000875

28 Vissers DCJ, Voeten HACM, Nagelkerke NJD, *et al.* The Impact of Pre-Exposure

For peer review only

Prophylaxis (PrEP) on HIV Epidemics in Africa and India: A Simulation Study. *PLoS One* 2008;**3**:e2077.https://doi.org/10.1371/journal.pone.0002077

29 Rozhnova G, Heijne J, Bezemer D, *et al.* Elimination prospects of the Dutch HIV epidemic among men who have sex with men in the era of preexposure prophylaxis. *AIDS* 2018;**32**:2615–23. doi:10.1097/QAD.0000000000002050

30 A. van WD, Chantal den D, E. KME, *et al.* Double trouble: modelling the impact of low risk perception and high-risk sexual behaviour on chlamydia transmission. *J R Soc Interface* 2018;**15**:20170847. doi:10.1098/rsif.2017.0847

For peer review only

FIGURE TITLES

Figure 1: (a) Estimated prevalence of HIV (*y*-axis) for the three mixing assumptions and different mean time to ART-treatment (*x*-axis). The presented time to ART-treatment is for high-active individuals, for low-actives it is a factor 2.35 higher. (b) The same estimated prevalence as in (a), but now showing the prevalence separately and including the 95% credibility interval for the three mixing assumptions. In one simulation, each partnership parameter (estimated from data) was drawn from its distribution. With that set-up of drawn parameters, we calculated the prevalence. This was repeated 1000 times to obtain the credibility interval.

Figure 2: The effect of introducing PrEP to sexually high-actives. In (a) the *x*-axis shows different PrEP coverage levels and the *y*-axis the corresponding HIV prevalence. The three lines show: the HIV prevalence in the total population (black solid line), the HIV prevalence among high-actives (lighter short-dashed line), and the HIV prevalence among low-actives (darker long-dashed line). (b) depicts the effect of PrEP by looking at: (i) solely the reduction of susceptibility and no increased testing rate; and (ii) solely the increased testing rate and no reduced susceptibility.

For peer review only

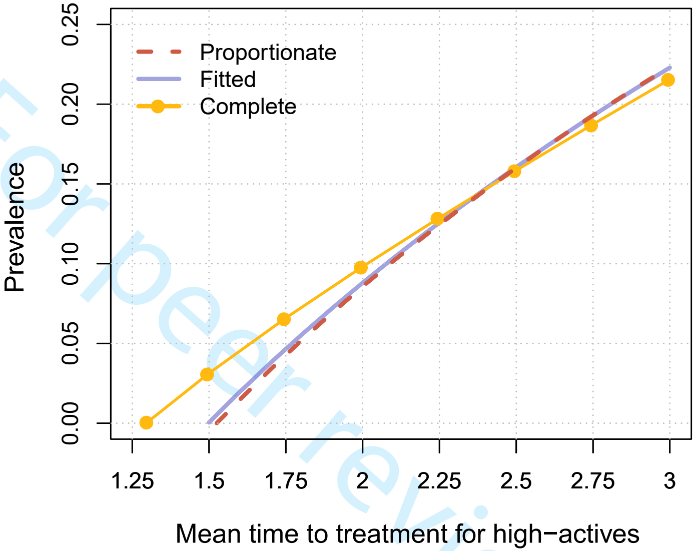

(a)

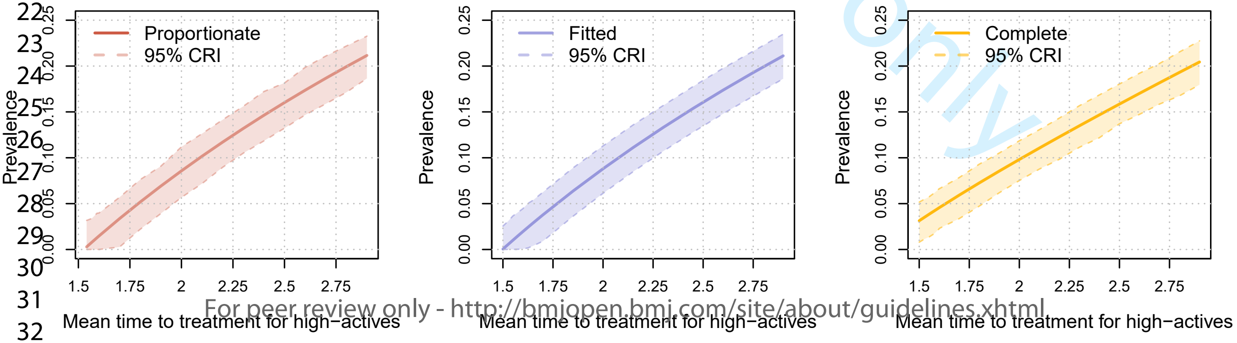

(b)

For peer review only

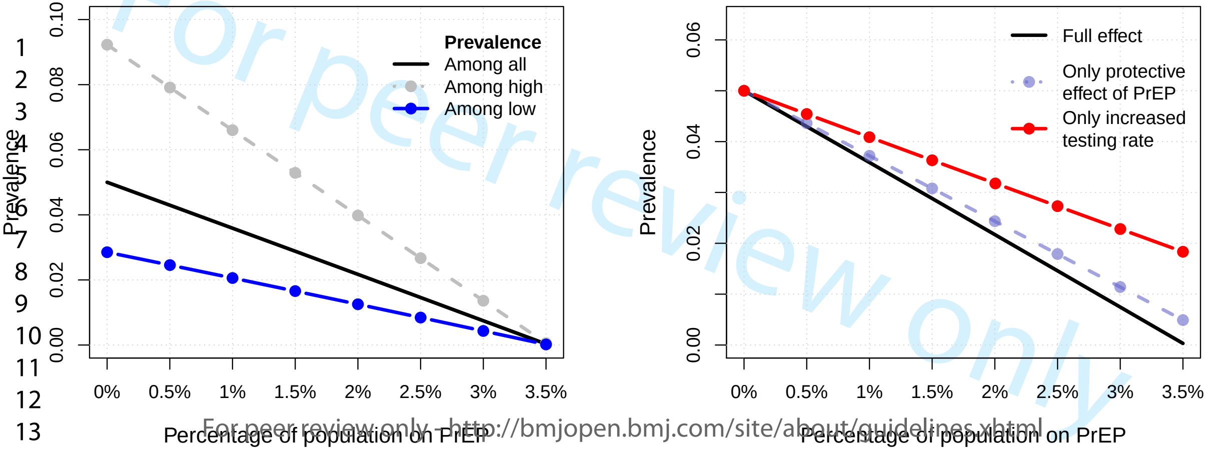

(a)

(b)

For peer review only - http://bmjopen.bmj.com/site/about/guidelines.xhtml

Online Supplementary Material: Introducing pre-exposure prophylaxis to prevent HIV acquisition among men who have sex with men in Sweden: insights from a mathematical pair-formation model

Disa Hansson, Susanne Strömdahl, KaYin Leung and Tom Britton

For peer review only

Contents

S1 Formulation of the model **2**
S1.1 Pair-formation model . . . 2
S1.2 Model of infection . . . 2

S2 Rate of new casual sex partners **5**
S2.1 Proportionate Mixing with respect to activity degree . . . 7
S2.2 Complete assortativity . . . 7
S2.3 Mixing determined by the proxy question . . . 8

S3 Data and parameter estimates **8**
S3.1 Data description . . . 8
S3.2 Scaling of the rate of finding a new casual sex partner . . . 8
S3.3 Time since last HIV-test . . . 9
S3.4 Distribution of parameters . . . 9
S3.5 Proxy of partners' activity degree . . . 12
S3.6 Final estimates of the rates of acquiring new casual sex partners . . . 13

S4 Additional results **15**
S4.1 Not distinguishing individuals according to activity degree . . . 15
S4.2 Not including the different infectious stages acute and chronic . . . 15
S4.3 Range of 95% CRI . . . 16
S4.4 Effect of different PrEP effectiveness in reducing susceptibility . . . 16
S4.5 Effect of giving PrEP to low-actives instead of high-actives . . . 16
S4.6 PrEP coverage for alternative transmission probabilities . . . 19
S4.7 Short-term effect of different PrEP strategies . . . 20

S5 Finding the endemic prevalence, a deterministic approximation **22**
S5.1 Single states . . . 24
S5.2 Partnership states . . . 25
S5.2.1 Susceptible not on PrEP . . . 25
S5.2.2 Acute infectious individuals not on PrEP . . . 27
S5.2.3 Chronic infectious not on PrEP . . . 28
S5.2.4 Treated individual . . . 29
S5.2.5 Individuals on PrEP . . . 29

For peer review only - http://bmjopen.bmj.com/site/about/guidelines.xhtml

S1 Formulation of the model

S1.1 Pair-formation model

We consider a sexually active same-sex population where new individuals enter the sexually active population according to an exponential distribution with rate μn, and each individual leaves the sexually active population at rate μ. The size of the sexually active population will therefore fluctuate around the value n, which is assumed to be large. Individuals enter the sexually active population without a steady partner. The rate at which an individual who is single enters into a partnership is ρP_0, where P_0 is the fraction of single individuals in the population. This means that the higher the fraction of single individuals, the higher the pair-formation rate. Individuals can have at most one steady partner at a time and the separation rate for each partnership is denoted σ. Therefore, a partnership lasts for an exponential time with mean duration $1/(\sigma + 2\mu)$.

The partnership network is assumed to be stable, i.e. the proportion of singles remains at P_0 for all t. We can then express P_0 (and the proportion $P_1 = 1 - P_0$ of individuals with a partner) in terms of model parameters [S1]:

$$P_0 = \frac{\sqrt{(\sigma + 2\mu)(4\rho + \sigma + 2\mu)} - (\sigma + 2\mu)}{2\rho}. \tag{S1.1}$$

The rate of sexual acts within a partnership is denoted λ. Beside steady partners, individuals may have casual sex partners during steady partnerships as well as during single periods; the rate at which this occurs depends on the partnership status of the individual under consideration.

Up to this point, the pair-formation model described is the same as in [S2]. The first extension of the model from [S2] is to allow for individuals to be either low-active or high-active with regards to the number of casual sex partners. In our application, an individual is assumed to be sexually high-active if they have 15 or more sex partners per year. The fractions of sexually high-active and sexually low-active in the population are denoted π_h and π_l, respectively ($\pi_h + \pi_l = 1$).

Let α_{ij}^{rq} be the rate an individual with activity degree $r \in \{l = \text{low}, h = \text{high}\}$ and $i \in \{0, 1\}$ steady partners tries to find a casual sex partner with activity degree $q \in \{l = \text{low}, h = \text{high}\}$ and $j \in \{0, 1\}$ steady partners. For this attempt to succeed the individual must actually meet an individual with activity degree q and j steady partners, and therefore, the rate of actual casual sex is $\alpha_{ij}^{rq} P_j \pi_q$. For example, a single who is low-active has casual sex with another low-active single at rate $\alpha_{00}^{ll} P_0 \pi_l$, and with a high-active individual in a steady partnership at rate $\alpha_{01}^{lh} P_1 \pi_h$.

S1.2 Model of infection

As explained in the main text, to model an infection on the network we use a so-called SIR compartmental model (for a survey on stochastic SIR models see [S3]). Individuals can either be susceptible (S), infectious in the acute stage (A), infectious in the chronic stage (C), or on ART-treatment (T). The second extension of the model in [S2] is to allow for these two infectious stages. Once an individual becomes aware of their infection and starts ART-treatment they are interpreted as immune and can no longer transmit infection. The average duration of the acute infection stage is 2.9 months (= 0.24 years) [S4]. Hence, an individual goes from A to C at rate $\delta_a = 1/0.24$ years^{-1}.

Given an unprotected sexual contact (in our case anal intercourse) between an infectious and susceptible individual, there is a probability of transmission depending on stage of infection: p_A when in the acute stage and p_C when in the chronic stage. Therefore, the transmission rate for an infectious individual in the acute stage in a steady partnership with a susceptible individual is $p_A \lambda$, and the transmission rate in a casual sexual encounter is $p_A \alpha_{ij}^{rq}$. Note that the probabilities p_A and p_C of transmission are for the unprotected case, in reality some of the intercourses are with condom. Condom use may also differ with steady and casual sex partners.

The third extension to [S2] is that the time until diagnosis and the beginning of successful ART-treatment may depend on the degree of sexual activity. A sexually high-active individual is put on ART-treatment at rate γ_h and a sexually low-active at rate γ_l.

For peer review only - http://bmjopen.bmj.com/site/about/guidelines.xhtml

Table S1.1: **Summary of model parameters.** The partnership formation model parameters are given in the first part of the table and the parameters connected to the epidemic in the second part.

**Partnership parameters**	
n	average population size
μ	rate of leaving the sexually active population
ρ	partnership formation rate
σ	separation rate
λ	rate of sex acts within a steady partnership
π_h	fraction of high-active individuals
π_l	fraction of low-active individuals
ξ	rate for high-actives to start taking PrEP
α_{ij}^{rq}	rate for a r-active individual with i steady partners to try to have casual sex with a q-active with j steady partners
**Epidemic parameters**	
p_A	probability of infection in one unprotected anal intercourse during the acute infectious stage
p_C	probability of infection in one unprotected anal intercourse during the chronic infectious stage
γ_h	ART-treatment rate for high-actives
γ_l	ART-treatment rate for low-actives
γ_P	ART-treatment rate for high-actives on PrEP
δ_a	rate of going from acute infection stage to the chronic stage

The fourth and main extension is to introduce the possibility for a high-active to take PrEP which dramatically decreases the probability of getting infected with HIV. The rate a high-active initiate PrEP is denoted ξ, and in our model the use of PrEP reduces the per-act probability of infection by 86% [S5]. A high-active on PrEP is tested and, if HIV-positive, put on ART-treatment at a rate $\gamma_P = 1/0.24$ years^{-1}.

To summarise, the model is captured by 30 parameters (20 free parameters): n, μ, ρ, σ, λ, p_A, p_C, δ_a, ξ; the fraction high-actives π_h and the fraction and low-actives $\pi_l = 1 - \pi_h$; the 16 parameters α_{ij}^{rq} (8 free), where $r, q \in \{l, h\}$ and $i, j \in \{0, 1\}$; and the three γ_P, γ_h and γ_l, where $\gamma_h = 2.349\gamma_l$ as described later in Section S3.3. We provide an overview of the notation in Table S1.1 and an illustration of the model in Figure S1.1. Note that, we could instead allow for low-actives, instead of high-actives, to be offered PrEP. This possibility will be briefly explored to be compared to the effect of targeting HIV high-risk individuals for a PrEP intervention programme.

For peer review only - http://bmjopen.bmj.com/site/about/guidelines.xhtml

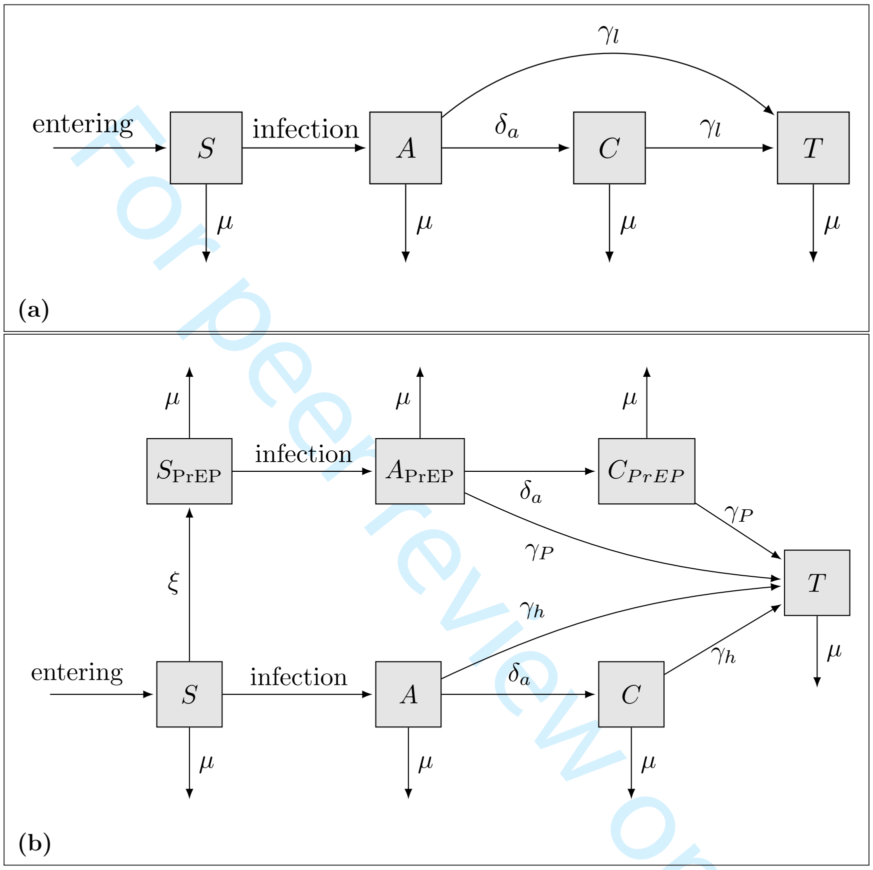

Figure S1.1: Representation of possible states a low-active (a) and a high-active (b) can be in. Individuals enter the MSM population as singles into the S compartment. High-actives move to S_{PrEP} at a rate ξ whereas low-actives can never start to use PrEP. Susceptible individuals who acquire infection move to the A compartment (acute infection). Individuals in the A compartment can move to the C (chronic infection) at rate δ_a or to the T (ART-treatment) compartment. The rate an individual moves to the T compartment is γ_P for a high-active on PrEP, γ_h for a high-active not on PrEP, and γ_l for a low-active. Individuals in the T compartment stays there until they leave the sexually active population. The rate of leaving the sexually active population is denoted μ.

For peer review only - http://bmjopen.bmj.com/site/about/guidelines.xhtml

S2 Rate of new casual sex partners

From our egocentric data on the sexual behaviour, we can determine if a participant is low-active or high-active and if he was single or in a steady partnership while having casual sex. It is therefore possible to obtain an estimate of the rate that an $r-$active individual with i steady partners finds new casual sex partners, let us denote this by $\alpha_{i\cdot}^{r\cdot}$, where $r \in \{h = \text{high}, l = \text{low}\}$ and $i \in \{0,1\}$. These rates ($\alpha_{i\cdot}^{r\cdot}$) can be used to express the different α_{ij}^{qr} for different mixing assumptions.

Before we give the equations of the observable $\alpha_{i\cdot}^{r\cdot}$ in terms of the sought α_{ij}^{qr}, we will present some symmetry arguments which reduces the number of free parameters. To begin with, we have 16 different parameters α_{ij}^{rq}. For symmetry reasons, when disregarding the activity degrees, the total rate in the population at which singles have casual sex with individuals in a partnership needs to equal the rate at which individuals in partnership have casual sex with singles. Assume that we have a population of size $n = n_0+n_1$, where $n_0 = nP_0$ is the number of individuals without a steady partner and $n_1 = nP_1$ is the number with a steady partner. A similar consistency criterion as for the rate of casual contacts disregarding heterogeneity in activity degree must also hold for the model with two activity degrees: the rate low-active singles ($n_0\pi_l$ in the population) have casual sex with high-active singles must equal the rate high-active singles ($n_0\pi_h$ in the population) have casual sex with low-active singles, i.e.

$$n_0\pi_l\alpha_{00}^{lh}P_0\pi_h = n_0\pi_h\alpha_{00}^{hl}P_0\pi_l,$$

which simplifies to

$$\alpha_{00}^{lh} = \alpha_{00}^{hl}.$$

Consistency hence require that

$$\alpha_{11}^{lh} = \alpha_{11}^{hl}, \quad \alpha_{10}^{lh} = \alpha_{01}^{hl}, \quad \alpha_{01}^{lh} = \alpha_{10}^{hl},$$

and

$$\alpha_{10}^{ll} = \alpha_{01}^{ll}, \quad \alpha_{10}^{hh} = \alpha_{01}^{hh},$$

or in one equation:

$$\alpha_{ij}^{rq} = \alpha_{ji}^{qr}. \tag{S2.1}$$

This reduces the number of free parameters to 10.

Let us now express the rate that an $r-$active individual with i steady partners finds new casual sex partners, $\alpha_{i\cdot}^{r\cdot}$, in terms of α_{ij}^{rq}

$$\begin{cases} \alpha_{0\cdot}^{h\cdot} = (\alpha_{00}^{hl}P_0 + \alpha_{01}^{hl}P_1)\pi_l + (\alpha_{00}^{hh}P_0 + \alpha_{01}^{hh}P_1)\pi_h \\ \alpha_{0\cdot}^{l\cdot} = (\alpha_{00}^{ll}P_0 + \alpha_{01}^{ll}P_1)\pi_l + (\alpha_{00}^{lh}P_0 + \alpha_{01}^{lh}P_1)\pi_h \\ \alpha_{1\cdot}^{h\cdot} = (\alpha_{10}^{hl}P_0 + \alpha_{11}^{hl}P_1)\pi_l + (\alpha_{10}^{hh}P_0 + \alpha_{11}^{hh}P_1)\pi_h \\ \alpha_{1\cdot}^{l\cdot} = (\alpha_{10}^{ll}P_0 + \alpha_{11}^{ll}P_1)\pi_l + (\alpha_{10}^{lh}P_0 + \alpha_{11}^{lh}P_1)\pi_h. \end{cases} \tag{S2.2}$$

This system of equations can later together with a proportionate mixing or complete assortativity assumption be solved, the solutions can be found in Section S2.1 and section S2.1, respectively.

For the case of an assortativity between the proportionate mixing and complete assortativity, we need further information than the $\alpha_{i\cdot}^{r\cdot}$ provides. With the help of a proxy question on participants' partners sexual behaviour (explained in Section S3.5), we can additionally estimate more detailed rates than $\alpha_{i\cdot}^{r\cdot}$: the rate

For peer review only - http://bmjopen.bmj.com/site/about/guidelines.xhtml

that an $r-$active individual with i steady partners finds new casual sex partners that are $q-$active, $\alpha_{i\cdot}^{rq}$. The following hold for these rates

$$\begin{cases} \alpha_{0\cdot}^{hh} = (\alpha_{00}^{hh}P_0 + \alpha_{01}^{hh}P_1)\pi_h \\ \alpha_{0\cdot}^{hl} = (\alpha_{00}^{hl}P_0 + \alpha_{01}^{hl}P_1)\pi_l \\ \alpha_{0\cdot}^{lh} = (\alpha_{00}^{lh}P_0 + \alpha_{01}^{lh}P_1)\pi_h \\ \alpha_{0\cdot}^{ll} = (\alpha_{00}^{ll}P_0 + \alpha_{01}^{ll}P_1)\pi_l \\ \alpha_{1\cdot}^{hh} = (\alpha_{10}^{hh}P_0 + \alpha_{11}^{hh}P_1)\pi_h \\ \alpha_{1\cdot}^{hl} = (\alpha_{10}^{hl}P_0 + \alpha_{11}^{hl}P_1)\pi_l \\ \alpha_{1\cdot}^{lh} = (\alpha_{10}^{lh}P_0 + \alpha_{11}^{lh}P_1)\pi_h \\ \alpha_{1\cdot}^{ll} = (\alpha_{10}^{ll}P_0 + \alpha_{11}^{ll}P_1)\pi_l. \end{cases} \tag{S2.3}$$

This gives us 8 equations with 10 unknowns. The data does not provide information on whether a casual sex partner is single or in a steady partnership. Therefore, we need to make further assumptions concerning the relation (ratio) between the rate of finding a casual sex partner that are single and the rate of finding a casual sex partner that are in a steady partnership. Let us consider one high-active individual, we assume that the rate of casual contact with a high-active in a partnership compared to the rate with a high-active single, is the same regardless if the considered individual is in a partnership or not, i.e.

$$\frac{\alpha_{00}^{hh}}{\alpha_{01}^{hh}} = \frac{\alpha_{10}^{hh}}{\alpha_{11}^{hh}}, \tag{S2.4}$$

and similarly, for a low-active individual (finding another low-active)

$$\frac{\alpha_{00}^{ll}}{\alpha_{01}^{ll}} = \frac{\alpha_{10}^{ll}}{\alpha_{11}^{ll}}. \tag{S2.5}$$

However, it turns out that the two equations are linearly dependent, we therefore need one more equation to be able to solve the system of equations (S2.3). We make the same kind of assumption but for the case when a high-active meets a low-active: for a high-active individual, the rate of casual contact with a low-active in a partnership compared to the rate with a low-active being single is the same regardless if the high-active individual is in a partnership or not

$$\frac{\alpha_{00}^{hl}}{\alpha_{01}^{hl}} = \frac{\alpha_{10}^{hl}}{\alpha_{11}^{hl}}, \tag{S2.6}$$

which further implies that $\frac{\alpha_{00}^{lh}}{\alpha_{01}^{lh}} = \frac{\alpha_{10}^{lh}}{\alpha_{11}^{lh}}$.

From the consistency criteria given in Equation (S2.1), Equation (S2.4)-(S2.6) the system of Equations (S2.3) can therefore be solved. Let $D_{qr} = \pi_q(\alpha_{0\cdot}^{qr}P_0 + \alpha_{1\cdot}^{qr}P_1)$, then we can express the rate for a $q-$active to try to find a r-active as:

$$\alpha_{ij}^{qr} = \frac{\alpha_{i\cdot}^{qr}\alpha_{j\cdot}^{rq}}{D_{qr}}. \tag{S2.7}$$

Note that, by consistency

$$D_{hl} = \pi_h(\alpha_{0\cdot}^{hl}P_0 + \alpha_{1\cdot}^{hl}P_1) = \pi_l(\alpha_{0\cdot}^{lh}P_0 + \alpha_{1\cdot}^{lh}P_1) = D_{lh}. \tag{S2.8}$$

To conclude, the consistency criteria given in Equation (S2.1), that $D_{hl} = D_{lh}$, together with the assumption given in Equation (S2.4)-(S2.6) implies that we can write α_{ij}^{qr} as a product:

$$\alpha_{ij}^{qr} = \omega_i^{qr}\omega_j^{rq}, \tag{S2.9}$$

where

$$\omega_i^{qr} = \frac{\alpha_{i\cdot}^{qr}}{\sqrt{D_{qr}}}.$$

For peer review only - http://bmjopen.bmj.com/site/about/guidelines.xhtml

S2.1 Proportionate Mixing with respect to activity degree

Proportionate mixing with respect to activity degree means that an individual has no preference regarding which type, high or low-active, it has casual sex with. An individual chooses at random of the potential casual sex attempts in the population. The fraction of potential high-active and low-active casual sex partners will not only depend on the sizes of the two groups, but also the rates at which they try to find new casual sex partners. If the sizes of the groups would be equal, someone trying to find a new casual sex partner would by chance meet a high-active more often since the high-active try to find a new casual sex partner more often than the low-active. Proportionate mixing then implies that the rate a low-active single has casual sex with a high-active single will be

$$\alpha_{00}^{lh} P_0 \pi_h = \alpha_{0\cdot}^{l\cdot} \times \frac{\alpha_{0\cdot}^{h\cdot} P_0 \pi_h}{\alpha_{0\cdot}^{l\cdot} P_0 \pi_l + \alpha_{0\cdot}^{h\cdot} P_0 \pi_h + \alpha_{1\cdot}^{l\cdot} P_1 \pi_l + \alpha_{1\cdot}^{h\cdot} P_1 \pi_h}$$

i.e. the rate a low-active single has casual sex, times the proportion of all casual sex partners that are from high-active singles. In terms of α_{ij}^{qr} we have that

$$\alpha_{ij}^{qr} = \alpha_{i\cdot}^{q\cdot} \times \frac{\alpha_{j\cdot}^{r\cdot}}{\alpha_{0\cdot}^{l\cdot} P_0 \pi_l + \alpha_{0\cdot}^{h\cdot} P_0 \pi_h + \alpha_{1\cdot}^{l\cdot} P_1 \pi_l + \alpha_{1\cdot}^{h\cdot} P_1 \pi_h}$$

The expression for α_{ij}^{qr} can also be found by using that proportionate mixing implies that $\omega_i^{lh} = \omega_i^{ll}$ and $\omega_i^{hh} = \omega_i^{hl}$. By dropping the second superscript and simply write ω_i^l and ω_i^h, yields that

$$\alpha_{ij}^{qr} = \omega_i^q \omega_j^r.$$

This together with the system of equations (S2.2) gives the above solution for α_{ij}^{rq}.

S2.2 Complete assortativity

Complete assortativity in whom you choose to have casual sex with regarding activity-degree implies that no casual sex occurs between high and low active: $\alpha_{ij}^{hl} = 0$. With Equation (S2.9) the system of Equations in (S2.2) can be written as

$$\begin{cases} \alpha_{0\cdot}^{h\cdot} = (\alpha_{00}^{hh} P_0 + \alpha_{01}^{hh} P_1)\pi_h \\ \alpha_{0\cdot}^{l\cdot} = (\alpha_{00}^{ll} P_0 + \alpha_{01}^{ll} P_1)\pi_l \\ \alpha_{1\cdot}^{h\cdot} = (\alpha_{10}^{hh} P_0 + \alpha_{11}^{hh} P_1)\pi_h \\ \alpha_{1\cdot}^{l\cdot} = (\alpha_{10}^{ll} P_0 + \alpha_{11}^{ll} P_1)\pi_l \end{cases} \overset{S2.9}{\Longleftrightarrow} \begin{cases} \alpha_{0\cdot}^{h\cdot} = \omega_0^{hh}(\omega_0^{hh} P_0 + \omega_1^{hh} P_1)\pi_h \\ \alpha_{0\cdot}^{l\cdot} = \omega_0^{ll}(\omega_0^{ll} P_0 + \omega_1^{ll} P_1)\pi_l \\ \alpha_{1\cdot}^{h\cdot} = \omega_1^{hh}(\omega_0^{hh} P_0 + \omega_1^{hh} P_1)\pi_h \\ \alpha_{1\cdot}^{l\cdot} = \omega_1^{ll}(\omega_0^{ll} P_0 + \omega_1^{ll} P_1)\pi_l \end{cases}$$

with the solution

$$\omega_i^{rr} = \frac{\alpha_{i\cdot}^{r\cdot}}{\sqrt{\alpha_{0\cdot}^{r\cdot} P_0 \pi_r + \alpha_{1\cdot}^{r\cdot} P_1 \pi_r}}.$$

For example, the rate a high-active single finds new casual sex partners that also are high-active, but in a partnership, becomes

$$\alpha_{01}^{hh} P_1 \pi_h = \omega_0^{hh} \omega_1^{hh} P_1 \pi_h = \alpha_{0\cdot}^{h\cdot} \times \frac{\alpha_{1\cdot}^{h\cdot} P_1 \pi_h}{\alpha_{0\cdot}^{h\cdot} P_0 \pi_h + \alpha_{1\cdot}^{h\cdot} P_1 \pi_h} = \alpha_{0\cdot}^{h\cdot} \times \frac{\alpha_{1\cdot}^{h\cdot} P_1}{\alpha_{0\cdot}^{h\cdot} P_0 + \alpha_{1\cdot}^{h\cdot} P_1}.$$

For peer review only - http://bmjopen.bmj.com/site/about/guidelines.xhtml

S2.3 Mixing determined by the proxy question

To obtain the different α_{ij}^{qr} for the case of an assortativity between the proportionate mixing case and complete assortativity, we use the 8 different $\alpha_{i\cdot}^{qr}$ estimated from data via the proxy question (see Table S3.2) and Equation S2.9 to get the 16 α_{ij}^{qr}, i.e.

$$\alpha_{ij}^{qr} = \omega_i^{qr}\omega_j^{rq} = \frac{\alpha_{i\cdot}^{qr}\alpha_{j\cdot}^{rq}}{D_{qr}}.$$

S3 Data and parameter estimates

We will here describe the data gathering, calibration of the model and the parameter estimates obtained from the STI-clinic.

S3.1 Data description

The data used in this study was gathered at a gay-friendly STI-testing clinic in Stockholm, Sweden. Collection of data took place between February 2 and December 15, 2015. Participants first reported demographic information and the total number of sex partners during the last 12 months, then the participants were asked to fill in an app-based timeline follow-back (TLFB) questionnaire.

In the TLFB questionnaire participants were asked to mark up to 10 of their most recent sex partners on a 12-month timeline. Participants did themselves label their partners into one of four partnership types: 1) casual unknown sex partner, 2) casual known sex partner, 3) regular sex partner (regular sex partner but not a 'love' relationship), and 4) main sex partner (a loving relationship, e.g. boyfriend/husband). For casual sex partners, a partner was represented by a single point on the interactive timeline, and a steady sex partner was represented by marking the start and end date of the relationship. For each sex partner on the timeline the participants could report: the partnership type 1) to 4); age of partner; frequency of each sex type (oral/anal; receptive/insertive); frequency of condom use; if the sex took place in Sweden or abroad; drug use and transactions in connection to sex with each partner; and if the participant believed the sex partner had other sex partners concurrently. This last question on concurrency is by us here referred to as the proxy question (for activity-degree assortativity).

In total 403 participants completed the TLFB questionnaire, giving detailed information on 2112 different sex partners. However, for a participant to be included in this study the total number of sex partners and the proxy question need to be answered, as explained in the main manuscript, yielding the data-set of this study consisting of 368 participants and 1903 partners.

S3.2 Scaling of the rate of finding a new casual sex partner

Participants reported their total number of sex partners during a year. The maximum number of sex partners of a participant was 250. When dividing the population into a category of high-active ($\geq$ 15 sex partners a year) and one category low-active ($<$ 15 sex partners a year), 124 (33.7%) participants are defined as high-active and 244 (66.3%) are defined as low-active. The mean number of sex partners of high-actives is 33.21 (median 25, sd 32), and the mean number of low-actives is 5.96 (median 5, sd 3.2). The assumption that only the number of casual sex partners is affected by activity-degree is supported by our data: the mean number of steady partners for high-actives and low-actives is 1.37 and 1.39 respectively.

Additionally, the participants gave detailed information on their (up to) 10 most recent of these sex partners. Participants reported what type of partner these 10 most recent sex partners were, either casual or steady, and the timings of these partners on a timeline. As mentioned, a casual sex partner was reported as a cross on the timeline representing the date of sex and a steady sex partner was given by the start date and end date of the relationship. This timeline data is used to estimate, for example, the time until a new casual sex partner. However, the timeline data only considers data on up to 10 casual sex partners, when we in fact know that many participants have more than this. We therefore need to scale the rates of finding a new casual sex partner according to the total number of partners reported by the participants.

For peer review only - http://bmjopen.bmj.com/site/about/guidelines.xhtml

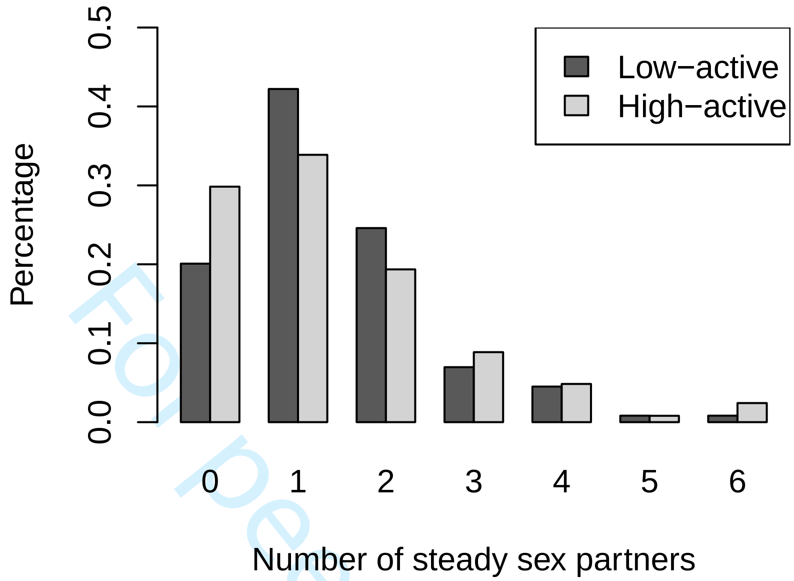

Figure S3.1: Distribution of steady sex partners of high-actives and low-actives, respectively.

The distribution of the number of steady sex partners of high-actives and low-actives are shown in Figure S3.1, as seen the distributions are similar. The mean number of steady sex partners per year of a high-active is 1.37 (sd 1.38), from which we estimate that the mean number of casual sex partners is $\mu_h = 31.84$ (total number - number of steady). The mean number of steady sex partners of a low-active is 1.39 (sd 1.15) and the mean number of casual sex partners is therefore $\mu_l = 4.57$. We use μ_h and μ_l to scale the rates of finding casual sex partners, since the detailed data from where timings of casual sex partners was given only include (up to) the 10 most recent sexual partners. In the detailed data, the mean number of casual sex partners is 5.35 for high-active individuals and 3.12 for low-active individuals. Hence the scaling factor will be $\mu_h/5.35$ for high-active individuals and $\mu_l/3.12$ for low-active individuals.

Finally, participants reported to have (a mean value of) 1.4 sex acts with each casual sex partner.

S3.3 Time since last HIV-test

In Table S3.1 the time since the last HIV-test are shown, indicating that sexually high-active participants test themselves more often than sexually low-active participants. The rate to successful ART-treatment is denoted γ_h and γ_l for high-actives and low-actives, respectively. Calculating ML-estimates of the time since the last HIV-test, assuming an exponential distribution, we find that the testing rate of a high-active is 2.349 higher than the testing rate of a low-active. We will further assume that the same relationship holds for the time to successful ART-treatment, i.e. the rate of initiating successful ART-treatment for an infected high-active is 2.349 times higher than the rate of initiating ART-treatment for an infected low-active. Therefore, we fix $\gamma_h = 2.349\gamma_l$ such that we only need to vary one of the parameters.

S3.4 Distribution of parameters

The time durations in the model, such as the time until finding a new steady sex partner, are assumed to be exponential and are hence specified by their rates. With this assumption we calculate a point estimate and its standard error. In general, if we are looking at something occurring at an exponential rate, α say, then

For peer review only - http://bmjopen.bmj.com/site/about/guidelines.xhtml

Table S3.1: **Time since last HIV-test separated by degree of activity.**

	High	(%)	Low	(%)	Σ
< 6 mths ago	85	(68.5)	121	(49.6)	206
6 to 12 mths ago	20	(16.1)	54	(22.1)	74
1 to 5 yrs ago	9	(7.3)	35	(14.3)	44
> 5 yrs ago	0	(0)	11	(4.5)	11
No answer	10	(8.1)	14	(5.7)	24
Do not remember	0	(0)	2	(0.1)	2
Σ	124	(100)	244	(100)	368

the number of events N occurring during a time interval of length t is Poisson distributed with parameter αt ($N \sim Poisson(\alpha t)$). Observing n events during a time t leads to the ML-estimate $\hat{\alpha} = n/t$. The variance of the estimate equal $Var\,(\hat{\alpha}) = Var\,(N/t) = Var(N)/t^2 = \alpha/t$, which leads to a standard error of the estimate of $\sqrt{\hat{\alpha}/t}$.

For example, in calculating the estimate of the rate for a high-active in a steady partnership to find a high-active casual sex partner, we do as follows: find the total time high-active individuals are in a steady partnership (T_1^h), then find the number of casual sex partners that occur during that time that is with someone that also is high-active (N_1^{hh}) and multiply it with the scaling factor from Section S3.2 ($N_1^{hh} \times \frac{\mu_h}{5.35}$). The point estimate is given by $\hat{\alpha}_{1\cdot}^{hh} = \left(N_1^{hh} \times \frac{\mu_h}{5.35}\right)/T_1^h$ and its standard error by $s.e.(\hat{\alpha}_{1\cdot}^{hh}) = \sqrt{\hat{\alpha}_{1\cdot}^{hh}/T_1^h}$.

For the number of occurrences of anal intercourses (AIs) in a steady partnership, the participants reported the number of acts during a 1-month period. Let m be the number of steady partners among all participants and let a_i denote the number of occurrences of AI with partner $i = 1, \ldots, m$. The estimated rate of AI, in units of months, is

$$\hat{\lambda} = \frac{\sum_{i=1}^m a_i}{\sum_{i=1}^m 1} = \frac{\sum_{i=1}^m a_i}{m},$$

and the standard error is

$$s.e(\hat{\lambda}) = \sqrt{\frac{\hat{\lambda}}{m}}.$$

In the data for casual sex partners, it is recorded if a condom was used (1) or not used (0) during receptive anal intercourse (RAI) and during insertive anal intercourse (IAI). To estimate the condom use in casual contacts we use a Bernoulli assumption and calculate the mean condom use during RAI and IAI, respectively. With a Bernoulli assumption we mean that, in each new casual sex act a condom is used with a probability, p_c say, independently of previous sex acts. Then the estimate $\hat{p}_c$ is given by the mean number times a condom was used. The standard error is given by

$$s.e(\hat{p}_c) = \sqrt{\frac{\hat{p}_c(1-\hat{p}_c)}{n}},$$

where n here is the number of observations, i.e. the number of casual sex partners where a binary response on condom use was given.

For condom use with a steady sex partner, participants could choose from a five-degree scale on how often a condom was used during RAI and during IAI: always (100%), often (75%), half of the times (50%), seldom (25%), and never (0%). Here, the participants did themselves, in a sense, give the mean number of times they used condom with a partner. Assume the data consist of m such steady partners with corresponding responses $(y_1, \ldots, y_m)$. The estimated condom use in steady partnerships, $\hat{p}_s$, is then the mean of the m reported values on the five-degree scale, and the distribution of $\hat{p}_s$ is approximated by the normal distribution

$$\hat{p}_s = \frac{1}{m}\sum_{i=1}^m y_i \approx N(p_s, \tau^2/m).$$

For peer review only - http://bmjopen.bmj.com/site/about/guidelines.xhtml

Table S3.2: **Estimates of partnership and epidemic parameters.**

**Partnership parameters from data**			
Parameter	Estimate	S.E.	Definition
$\sigma + 2\mu$	1.344/year	0.070	Rate of ending steady partnership
ρP_0	2.389/year	0.164	Rate of acquiring new steady partner
λ^*	29.793/year	0.837	Rate of sex acts (AI) within steady partnership
$\hat{\alpha}^h_{0\cdot}$	34.169/year	0.722	
$\hat{\alpha}^h_{1\cdot}$	29.227/year	0.707	
$\hat{\alpha}^l_{0\cdot}$	5.490/year	0.221	
$\hat{\alpha}^l_{1\cdot}$	3.729/year	0.168	
$\hat{\alpha}^{hh}_{0\cdot}$	25.809/year	0.628	
$\hat{\alpha}^{hl}_{0\cdot}$	8.360/year	0.357	
$\hat{\alpha}^{hh}_{1\cdot}$	23.728/year	0.637	
$\hat{\alpha}^{hl}_{1\cdot}$	5.499/year	0.307	
$\hat{\alpha}^{lh}_{0\cdot}$	3.338/year	0.172	
$\hat{\alpha}^{ll}_{0\cdot}$	2.152/year	0.138	
$\hat{\alpha}^{lh}_{1\cdot}$	2.682/year	0.143	
$\hat{\alpha}^{ll}_{1\cdot}$	1.046/year	0.089	
q_s^{RAI}	51.9%	2.4%	Condom use steady partner RAI
q_s^{IAI}	56.2%	2.3%	Condom use steady partner IAI
$\%RAI_s$	49%	-	Percentage of steady RAI and IAI acts that are RAI
q_c^{RAI}	62.8%	2.5%	Condom use casual partner RAI
q_c^{IAI}	63.1%	2.6%	Condom use casual partner IAI
$\%RAI_c$	52%	-	Percentage of casual RAI and IAI partners that are RAI

Note AI: anal intercourse; RAI: receptive anal intercourse; IAI: insertive anal intercourse

Where p_s is the true expected value of the condom use in steady partnerships and τ is the standard deviation of the condom use. The estimate of τ^2 is $\frac{1}{m-1}\sum_{i=1}^{m}(y_i - \hat{p}_s)^2$. The standard error of the estimated condom use with steady sex partners is then given by

$$s.e.(\hat{p}_s) = \hat{\tau}/\sqrt{m} = \sqrt{\frac{\frac{1}{m-1}\sum_{i=1}^{m}(y_i - \hat{p}_s)^2}{m}}.$$

With the estimated condom use during RAI and during IAI, we calculated the overall mean condom use by taking the weighted average of these two estimates. See Table S3.2 for condom use estimates and the proportion (weights) of sex acts that are RAI and IAI, from these values we can calculate the overall condom use for steady partners

$$q_s = q_s^{RAI} \times \%RAI_s + q_s^{IAI} \times \%IAI_s = 0.519 \cdot 0.49 + 0.562 \cdot 0.51 = 0.541,$$

and for casual sex partners

$$q_c = q_c^{RAI} \times \%RAI_c + q_c^{IAI} \times \%IAI_c = 0.628 \cdot 0.52 + 0.631 \cdot 0.48 = 0.629.$$

For peer review only - http://bmjopen.bmj.com/site/about/guidelines.xhtml

S3.5 Proxy of partners' activity degree

For the 10 most recent sex partners the participants responded to detailed questions. One of the detailed questions was what we referred to as the proxy question: 'Do you think that your sex partner had other sex partners than you during the same time frame that he/she met you?', with the possible answers

1. I know this person had sex with others
2. I think this person had sex with others
3. No, this person only had sex with me
4. I don't know

The percentages of the answers to the proxy question are given in Table S3.3.

Table S3.3: **Distribution of proxy variable on partners' activity degree.** One of the questions participants answered concerning their 10 most recent sex partners was whether they believed their partners had other concurrent sex partners. We use this as a proxy for partners activity-degree, as shown in the fourth and last column. With the help of a consistency criterion, Equation (S2.8), we assigned the partners of answer 4 as low-active.

Answer	Of all partners ($n = 1903$)	Of all casual partners ($n = 1424$)	Proxy for partner being
1. Yes	33.58%	32.65%	High-active
2. Think so	33.63%	38.76%	High-active
3. No	9.98%	3.30%	Low-active
4. Don't know	22.81%	25.28%	Low-active

If a participant answered either 1 or 2 for a partner, we will take this as a proxy for that the partner is high-active. If a participant answered 3, we will use this as a proxy for that the partner is low-active. We now need to decide what to do with the 25% of the partners that participants labelled as No. 4 on the proxy question, the partners that participants did not know whether they had other sex partners.

In the total population, consistency requires that the the number of casual sex acts high-actives have with low-actives needs to equal the number of casual sex acts low-actives have with high-actives. This criterion can be written, in terms of rates, as (Equation (S2.8))

$$D_{hl} = D_{lh}.$$

With this consistency we get help in determining how to assign the partners labelled as No. 4 on the proxy question. If we simply remove these partners entirely, the left-hand side of Equation (S2.8) becomes 0.18 and the right-hand side 1.93, very far from each other. Hence, to remove the partners of which the participants do not know (No. 4) yields a too big inconsistency. If we instead assign all these partners as low-active, we get that the left-hand side of Equation (S2.8) becomes 2.19 the right-hand side 1.93. This suggests that many of the partners participants labelled as No. 4 should be categorised as low-active.

In Table S3.4 we show (for the two choices of actions of answer No. 4 "I don't know"), the proportion of high-active individuals' casual sex partners that will be with low-actives and with high-actives, respectively; and the proportion of low-active individuals' casual sex partners that will be with low-actives and with high-actives, respectively. For example, if we assign all partners that participants labelled as No. 4 on the proxy question as low-active, we find that 34.3% of low-active individuals' casual sex partners will be with low-actives, and 65.7% will be with high-actives.

For peer review only - http://bmjopen.bmj.com/site/about/guidelines.xhtml

For peer review only

Table S3.4: **Consequence of the two assignments of the partners of which participants do not know if the partner did have other partners.** The column named *Removed* means that the partners of participants of which we do not know (answer 4 on the proxy question) were removed, and the column *As low-active* means we assigned those partners as low-active. First, for the two assignments, we show the proportion of low-active and high-active partners of participants who are low-active, then the same kind of proportion but for participants who are high-active. Then we show the values of the right-hand side (D_{lh}) and left-hand side D_{hl} of the consistency criterion Equation (S2.8)

	**Action on "Don't know"**	
Proportion	Removed	As low-active
low-low	0.067	0.343
low-high	0.933	0.657
high-low	0.021	0.220
high-high	0.979	0.780
Consistency		
D_{lh}	1.9345	1.9345
D_{hl}	0.1825	2.19

S3.6 Final estimates of the rates of acquiring new casual sex partners

We will now show the final estimates of the rates of finding new casual sex partners, using the three different mixing assumptions with respect to activity degree.

We found that the estimates for α_{ij}^{qr} that utilises the proxy question could be written as Equation S2.9

$$\alpha_{ij}^{qr} = \omega_i^{qr}\omega_j^{rq} = \frac{\alpha_{i\cdot}^{qr}\alpha_{j\cdot}^{rq}}{D_{qr}} = \frac{\alpha_{i\cdot}^{qr}\alpha_{j\cdot}^{rq}}{\pi_q(\alpha_{0\cdot}^{qr}P_0 + \alpha_{1\cdot}^{qr}P_1)},$$

where consistency requires that $D_{hl} = D_{lh}$ (Equation (S2.8)) needs to be fulfilled. Utilising Table S3.4, we found that assigning all partners that participants answered "I don't know" (answer No. 4) as low-active on the proxy question yielded a value of 2.19 for the left-hand side and a value of 1.93 of the right-hand side of Equation (S2.8), i.e. similar but not equal. We choose to work with the left-hand side, setting $D_{hl} = D_{lh} = 2.19$ when estimating the different α_{ij}^{qr}, which can be seen in Table S3.5. To make the comparison between the different mixing assumptions, we also use that $D_{hl} = D_{lh} = 2.19$ for all mixing assumptions. In Table S3.5 we show the estimates for proportionate and complete mixing under the assumption that $D_{hl} = D_{lh}$, where the values in parenthesis are the ones not requiring that $D_{hl} = D_{lh}$.

To quantify the degree of assortativity (with respect to activity degree) as a value θ between 0 and 1, where $\theta = 0$ means proportionate mixing and $\theta = 1$ means complete assortativity, we write

$$\alpha_{proxy}^{qr} = (1-\theta)\alpha_P^{qr} + \theta\alpha_C^{qr}.$$

Where α_P^{qr} is the rate, disregarding partnership status, a q-active tries to find an r-active under proportionate mixing and α_C^{qr} under complete assortativity. These α_{proxy}^{qr}, α_P^{qr}, and α_C^{qr} can be found by calculating the following quantity where α_{ij}^{qr}, $i, j \in 0, 1$ is taken from the corresponding mixing assumption,

$$\alpha_X^{qr} = \alpha_{11}^{qr}P_1^2 + (\alpha_{01}^{qr} + \alpha_{10}^{qr})P_0P_1 + \alpha_{00}^{qr}P_0^2.$$

Where P_0 is the proportion of the population without a steady sex partner and P_1 the proportion with a steady sex partner. As examples, using table S3.5 and the estimated value for $P_0 = 0.36$ and $P_1 = 0.64$, we get

$$\begin{aligned} \alpha_P^{hh} &= 62.20 \cdot P_1^2 + (72.44 + 72.44)P_0P_1 + 84.10 \cdot P_0^2 = 69.8, \\ \alpha_C^{hh} &= 80.13 \cdot P_1^2 + (93.02 + 93.02)P_0P_1 + 108.00 \cdot P_0^2 = 89.7, \\ \alpha_{proxy}^{hh} &= 68.26 \cdot P_1^2 + (74.25 + 74.25)P_0P_1 + 80.76 \cdot P_0^2 = 72.6. \end{aligned}$$

For peer review only - http://bmjopen.bmj.com/site/about/guidelines.xhtml

Table S3.5: **Estimates of rate parameters of finding new casual sex partner (years).** For the two extreme cases, proportionate mixing and complete assortativity, we give in parenthesis the casual sex rate estimates not requiring that $D_{hl} = D_{lh}$. Note that $\hat{\alpha}_{01}^{qr} = \hat{\alpha}_{10}^{rq}$ and is therefore not explicitly presented.

Parameter	Proxy	Prop	Complete
$\hat{\alpha}_{11}^{hh}$	68.26	62.20 (64.03)	80.13 (81.76)
$\hat{\alpha}_{11}^{hl}$	6.70	8.15 (8.17)	-
$\hat{\alpha}_{11}^{lh}$	6.70	8.15 (8.17)	-
$\hat{\alpha}_{11}^{ll}$	1.14	1.06 (1.04)	4.81 (4.81)
$\hat{\alpha}_{10}^{hh}$	74.25	72.44 (74.85)	93.02 (95.58)
$\hat{\alpha}_{10}^{hl}$	8.34	11.99 (12.03)	-
$\hat{\alpha}_{10}^{lh}$	10.19	9.46 (9.55)	-
$\hat{\alpha}_{10}^{ll}$	2.35	1.57 (1.53)	7.08 (7.08)
$\hat{\alpha}_{00}^{hh}$	80.76	84.10 (87.52)	108.00 (111.75)
$\hat{\alpha}_{00}^{hl}$	12.69	13.92 (14.06)	-
$\hat{\alpha}_{00}^{lh}$	12.69	13.92 (14.06)	-
$\hat{\alpha}_{00}^{ll}$	4.83	2.30 (2.26)	10.42 (10.42)

The value of θ that corresponds to these values is 0.141. Doing the same kind of calculations but for α^{lh} and α^{ll} yields the same θ.

Note that, there could exist disassortative with respect to activity-degree, however, we disregard from this since it seems unlikely in our application.

For peer review only

S4 Additional results

We will in this Section go through some additional results mentioned in the main manuscript.

S4.1 Not distinguishing individuals according to activity degree

Here we examine what happens if we do not divide the population according to active-degree but assume that everyone behaves in the same way regarding the number of casual sex partners and regarding the rate to ART-treatment. For the case when no one yet is on PrEP, we find that $R_0 = 1$ when the mean time to successful ART-treatment is 3.28 years. A prevalence of 5% is obtained for a mean time to ART-treatment of 3.57 years. In Figure S4.1 we show the effect of introducing PrEP in this model without high-actives and low-actives. We see that the PrEP coverage in such a population would need to exceed 5% to reach a prevalence close to 0 (in contrast to 3.5% as in the model where we have two activity degrees).

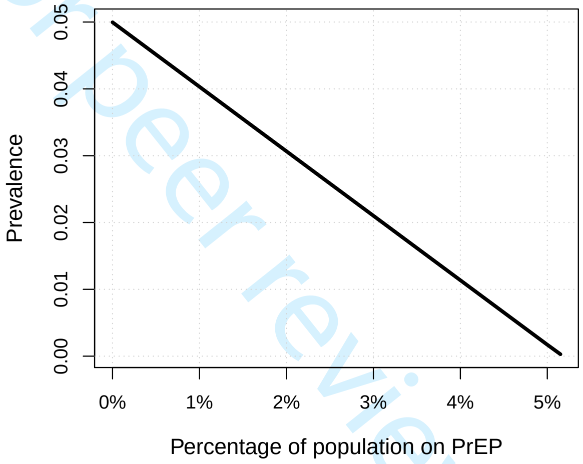

Figure S4.1: Effect of introducing PrEP in a population that is not separated according to activity degree, but where everyone is assumed to behave the same with regards of finding new casual sex partners.

S4.2 Not including the different infectious stages acute and chronic

In Figure 2b) in the main text, we saw that the reduction in susceptibility due to PrEP had a larger effect in reducing the prevalence than the increased testing rate of those on PrEP. The transmission probability of HIV is much higher in the acute infectious phase, the first 3 months following infection, than in the chronic phase. The reason for the lesser effect of an increased testing rate could be that it misses a large proportion of the acute stage.

To help verify that this is the case, we modified the model to not make a distinction between the acute and chronic stage; to only include one transmission probability during the whole infectious lifetime of an infected individual. We calibrate this transmission probability so that when no one is on PrEP, and the mean time to successful ART-treatment is 1.77 years for high-actives, the prevalence is equal to 5%. This is done to match the set-up of the analysis in Figure 2b). The transmission probability is then 0.0208 for the whole infectious time, instead of 0.1301 for the acute stage and 0.0098 for the chronic stage.

In Figure S4.2 it is seen that when only one infectious stage is included, the increased testing and diagnosis rate has as equally big impact on the reduction of the prevalence as the reduced susceptibility of PrEP. This implies that the lesser effect of the increased testing rate, that we found in Figure 2b) in the main manuscript, can be assigned to it missing the 3 month long acute stage. Because, when we in this analysis distributed

For peer review only - http://bmjopen.bmj.com/site/about/guidelines.xhtml

the increased transmission probability of the acute stage over an infected individual's lifetime, an increased testing rate got a bigger effect in reducing the prevalence than when we separated the infectious stages.

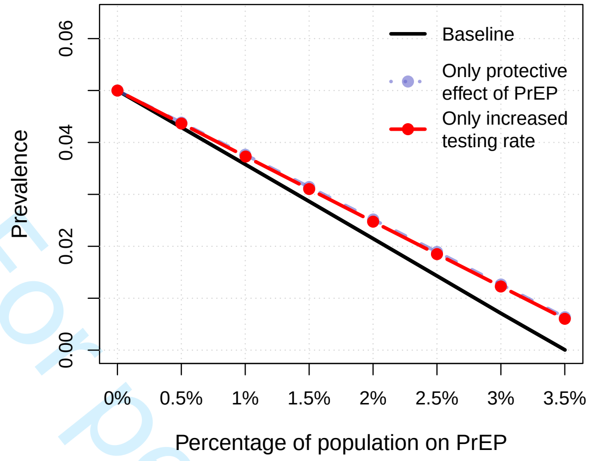

Figure S4.2: Same set up as corresponding figure in main text, Figure 2b), but with no distinction between the acute and chronic stage but only one infectious stage with one transmission probability.

S4.3 Range of 95% CRI

In Figure 1b) in the main manuscript we show the 95% credibility intervals of the estimated prevalence for all three mixing assumptions. This was done for different mean times to ART-treatment to obtain the credibility bands as in Figure S4.3a. Since it can be somewhat hard to compare the widths of said intervals in Figure 1b) and Figure S4.3a, we additionally calculated the range of each credibility intervals and show the boxplot of these values in Figure S4.3b. From Figure S4.3b it is seen that using the complete assortativity assumption regarding activity-degree has most narrow 95% credibility intervals. Moreover, the fitted assortativity has more narrow credibility intervals than the proportionate mixing assumption.

S4.4 Effect of different PrEP effectiveness in reducing susceptibility

In the introduction of the main manuscript we gave the estimated reduction of susceptibility of PrEP: 86% with a 95% confidence interval of 40 - 98% [S5]. To investigate the effect of this variability, we analysed the effect of introducing PrEP among high-active individuals with a 40% PrEP effectiveness, and then with a 98% PrEP effectiveness. The results can be seen in Figure S4.4. We see that, using the much less effective value of 40% instead of 86%, to reach an endemic prevalence close to 0 increases the needed PrEP coverage from 3.5% of the population (10.4% of all high-actives) to 4.4% of the population (13.1% of all high-actives).

S4.5 Effect of giving PrEP to low-actives instead of high-actives

In our analysis we mainly focus on the effect of high-active individuals accepting PrEP. If we instead want to determine the effect of targeting low-actives for PrEP, we could just reverse which activity-group is allowed to start taking PrEP. Here, we also show the results for when only low-actives are offered PrEP. As we can see from Figure S4.5, a much higher coverage (35%) is needed to reach the same long-term prevalence reduction compared to if high-actives were offered PrEP (3.5%) (Figure 2a in the main manuscript).

For peer review only - http://bmjopen.bmj.com/site/about/guidelines.xhtml

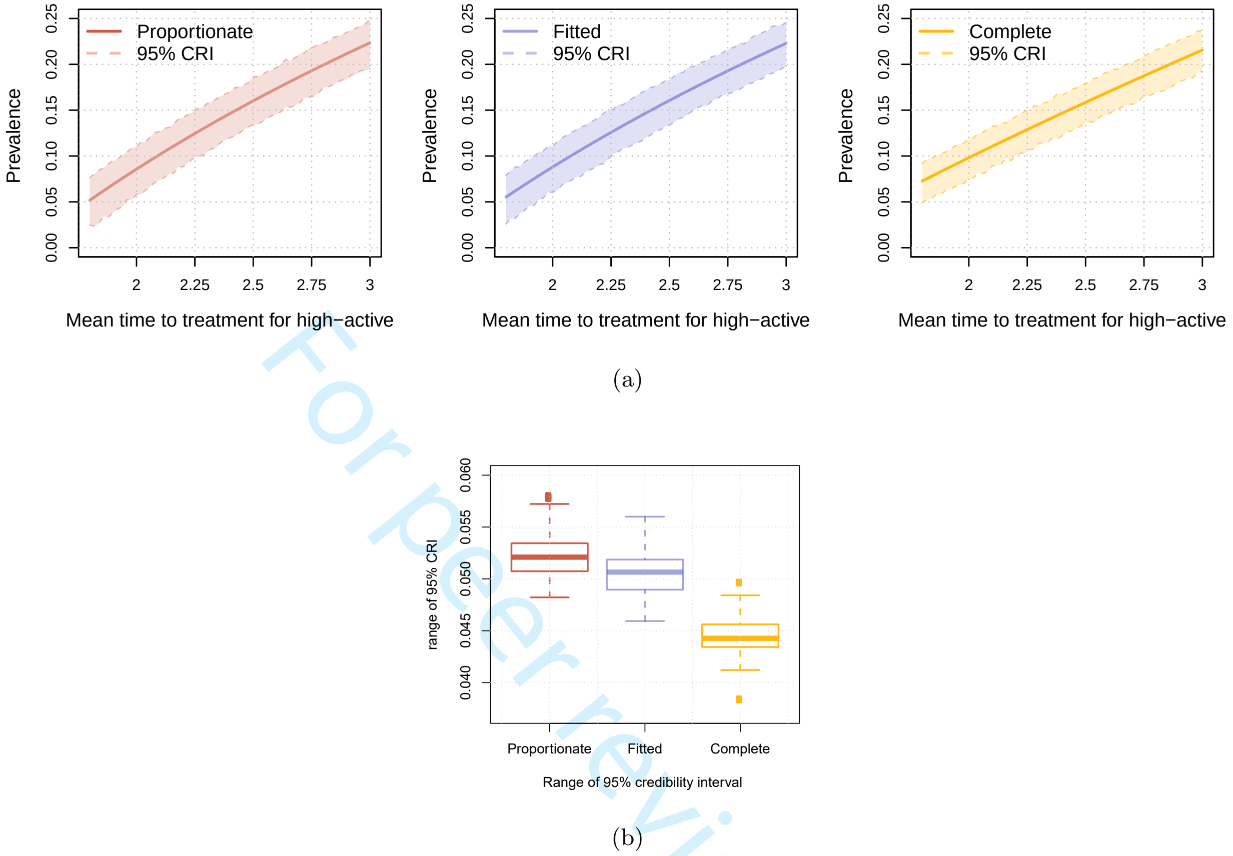

Figure S4.3: The width of the estimated 95% credibility intervals. (a) depicts the credibility bands from which we calculated the range of the credibility intervals, summarised as boxplots in (b).

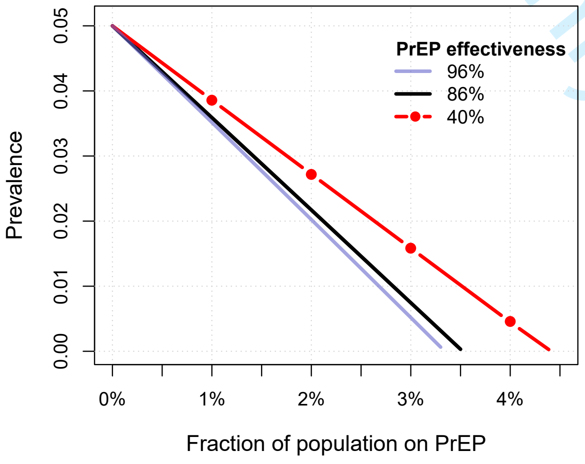

Figure S4.4: Endemic prevalence for different PrEP coverages and effectiveness.

For peer review only

For peer review only - http://bmjopen.bmj.com/site/about/guidelines.xhtml

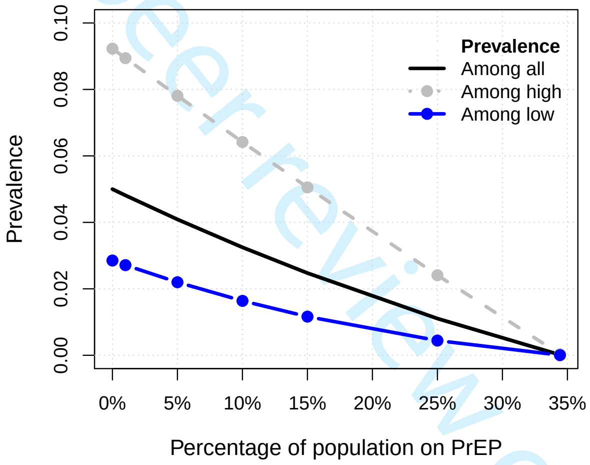

Figure S4.5: Effect of giving PrEP to low-actives instead of high-actives.

S4.6 PrEP coverage for alternative transmission probabilities

The transmission probabilities for unprotected receptive anal intercourse (URAI) during the acute and chronic stage are taken from the literature [S6] (0.1835 and 0.0138, respectively). To get the transmission probabilities during unprotected insertive anal intercourse (UIAI), we use an estimate of the relationship between the transmission probability between URAI and UIAI. The transmission probability for URAI is 2.39 times larger than the transmission probability for UIAI [S7]. Assuming equally many insertive as receptive acts, the transmission probability during the acute stage was set to

$$p_A = 0.1835 \times 0.5 + 0.1835 \times 0.5/2.39 = 0.1301,$$

and during the chronic stage

$$p_C = 0.0138 \times 0.5 + 0.0138 \times 0.5/2.39 = 0.0098.$$

We now want to study how robust our conclusion concerning PrEP coverage, to achieve an endemic prevalence close to 0%, is. We do this by altering the two transmission probabilities by setting them to $50\% - 150\%$ of their estimated values. For example: with 50% of the transmission probabilities, we have that $p_A = 0.0651$ and $p_C = 0.0049$; with 150% of the transmission probabilities, we have that $p_A = 0.1952$ and $p_C = 0.0147$. With given transmission probabilities p_A and p_C, we find the mean time to ART-treatment corresponding to a prevalence of 5%. With the different set-ups generating a prevalence of 5%, PrEP is introduced to high-active individuals. In Table S4.1 we show the different set-ups and the PrEP coverage needed to get an endemic prevalence close to 0. Additionally, we alter the two transmission probabilities one at a time in Table S4.2 and Table S4.3. In Table S4.2 we vary p_A but let p_C stay fixed at 0.0098. In Table S4.3 we vary p_C but let p_A stay fixed at 0.1301. We conclude by noting that the results are almost invariant to which set-up is used.

Table S4.1: **Robustness of the PrEP coverage to obtain a long-term prevalence close to 0: alteration of p_A and p_C.** Assuming no one is on PrEP, we first find other combinations than the one used in the main manuscript of the transmission probabilities and mean time to ART-treatment that generates a prevalence of 5%. With these different scenarios that generates a prevalence of 5%, we then study the needed PrEP coverage to obtain a long-term prevalence close to 0.

	**% of transmission probabilities** $p_A = 0.1301$ **and** $p_C = 0.0098$										
	50%	60%	70%	80%	90%	100%	110%	120%	130%	140%	150%
Time to ART-treatment	5.38	4.10	3.23	2.60	2.13	1.77	1.48	1.25	1.07	0.92	0.80
PrEP coverage for 0 prevalence	3.42%	3.43%	3.44%	3.47%	3.49%	3.52%	3.55%	3.58%	3.61%	3.65%	3.68%

Table S4.2: **Robustness of the PrEP coverage to obtain a long-term prevalence close to 0: alteration of p_A.** Same procedure as in Table S4.1, but only the transmission probability in the acute stage is altered. The transmission probability in the chronic stage is remained fixed at $p_C = 0.0098$.

	**% of transmission probabilities** $p_A = 0.1301$**.**										
	50%	60%	70%	80%	90%	100%	110%	120%	130%	140%	150%
Time to ART-treatment	2.76	2.55	2.35	2.15	1.96	1.77	1.59	1.42	1.26	1.12	0.98
PrEP coverage for 0 prevalence	3.44%	3.45%	3.46%	3.48%	3.50%	3.52%	3.55%	3.58%	3.61%	3.64%	3.67%

For peer review only - http://bmjopen.bmj.com/site/about/guidelines.xhtml

Table S4.3: **Robustness of the PrEP coverage to obtain a long-term prevalence close to 0: alteration of p_C.** Same procedure as in Table S4.1, but only the transmission probability in the chronic stage is altered. The transmission probability in the acute stage is remained fixed at $p_A = 0.1301$.

	**% of transmission probabilities $p_C = 0.0098$.**										
	50%	60%	70%	80%	90%	100%	110%	120%	130%	140%	150%
Time to ART-treatment	3.14	2.68	2.36	2.11	1.92	1.77	1.64	1.54	1.45	1.37	1.30
PrEP coverage for 0 prevalence	3.5%	3.5%	3.51%	3.51%	3.52%	3.52%	3.53%	3.53%	3.53%	3.54%	3.54%

S4.7 Short-term effect of different PrEP strategies

In our model, we assume that a sexually high-active start using PrEP at rate ξ. We then determine the lowest possible rate ξ that yields an equilibrium prevalence of 0% and calculate which PrEP coverage this corresponds to. The lowest PrEP coverage that *eventually* results in a 0% HIV prevalence is 3.5% of the population. This 'eventually' could be a very long time in the future. If no new HIV cases would occur, it would still take many years before the prevalence reaches 0%; the HIV prevalence would not reach 0% until the last person with HIV dies. However, HIV will effectively disappear when no new infections occur. Remember that, in our model we assume that diagnosis and the beginning of ART-treatment is the same as being uninfectious, and consequently, only individuals with undiagnosed HIV can transmit the infection. Hence, we will here study not only the prevalence but also the percentage undiagnosed HIV cases for different PrEP initiation rates, ξ.

In what follows, we will look at different rates ξ, where all rates result in an HIV prevalence of 0% in the equilibrium steady state. The lowest ξ we look at will therefore corresponds to an equilibrium PrEP coverage of 3.5% of the population ($\approx$ 10% of high-actives). As a starting point, before any high-active accepts PrEP, the prevalence is set to 5% and the percentage undiagnosed HIV cases to 0.21% (the model with $\xi = 0$ calibrated to data). In the left panel of Figure S4.6, we show the HIV prevalence (%) at different PrEP initiation rates, ξ, for 50 years after the beginning of a PrEP implementation programme. In the middle panel of Figure S4.6, we show the percentage of individuals that are infectious and undiagnosed. In the right panel of S4.6, we show the corresponding percentages of the population that are on PrEP for 50 years after the beginning of the PrEP programme. For the lowest rate ξ that results in an equilibrium HIV prevalence of 0%, we see that after 50 years the PrEP coverage has only had time to reach 2%, but the percentage undiagnosed has more than halved, and the prevalence has dropped from 5% to almost 4%. This can be compared to the scenario with a ξ that results in an equilibrium PrEP coverage of 11%. Then the PrEP coverage has reached a bit over 7% after 50 years, and the percentage undiagnosed HIV cases is only 1/20 of its value before the initiation of a PrEP programme (from 0.21% to 0.01%).

In Figure S4.7 we study, in more detail, the effects of different PrEP scenarios 10 and 20 years after their initiation. We look at both the prevalence and the percentage undiagnosed HIV cases. After 10 years, if the PrEP coverage has reached 5% (15% of all high-actives), the percentage undiagnosed HIV cases is reduced from 0.21% to 0.14%. Looking at 20 years after a PrEP programmes initiation and where the PrEP coverage has reached 5%, the percentage undiagnosed HIV cases is reduced to the low level of 0.04%. If the PrEP coverage on the other hand has reached almost all high-actives after 10 years, that is 30% of the population, then the percentage undiagnosed HIV cases is reduced to 0.03%. The same percentage of coverage after 20 years yields a percentage of 0.004% infectious and undiagnosed HIV cases; that is, almost no new HIV infections occur.

For peer review only - http://bmjopen.bmj.com/site/about/guidelines.xhtml

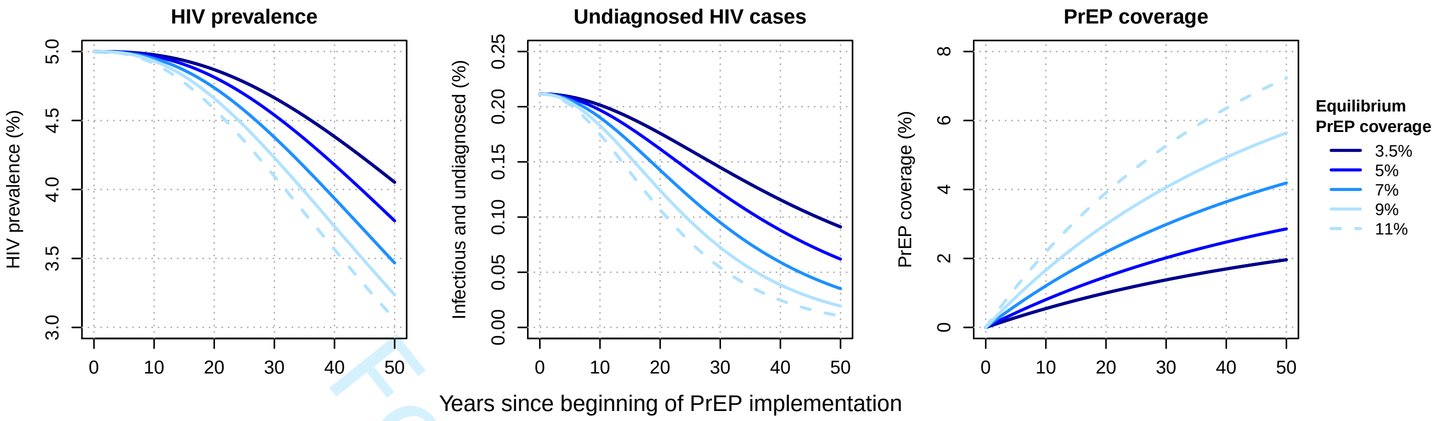

Figure S4.6: Effect of different PrEP scenarios on the HIV prevalence and new HIV cases (infectious and undiagnosed).

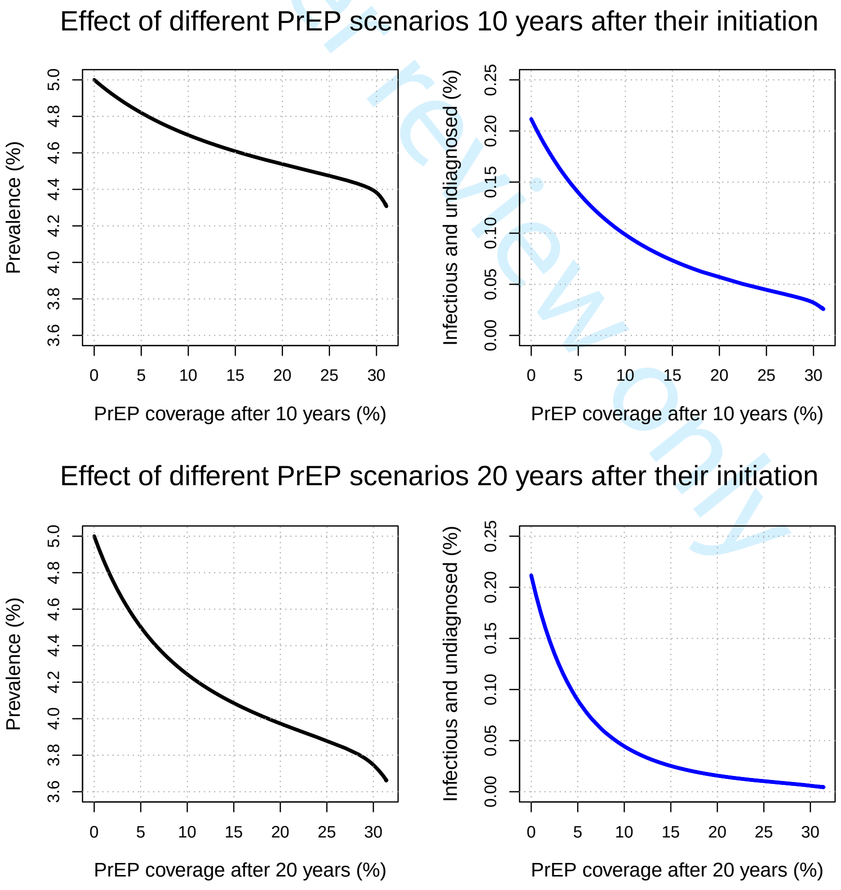

Figure S4.7: Effect of different PrEP scenarios 10 and 20 years after their initiation.

For peer review only - http://bmjopen.bmj.com/site/about/guidelines.xhtml

S5 Finding the endemic prevalence, a deterministic approximation

Both the basic reproduction number and the endemic level can be obtained by using a deterministic approximation of the stochastic model. Assuming that the population is large, it is then enough to consider expected values of the fraction of individuals that are susceptible, infectious, or recovered to obtain these quantities. In the following section, we explain how we construct the compartments and give the differential equations governed by the possible transitions within the network model. To find the endemic (or equilibrium) prevalence one needs to find the non-trivial steady state of the system of differential equations. The trivial solution is that everyone is susceptible.

In Figure S1.1 we showed the different infectious states an individual can be in; the four different infectious states are susceptible S, acute infectious A, chronic infectious C, and treated (on ART-treatment) T. We further divide the population into different types, these types specify: if an individual is single or in a partnership, if the individual is low-active or high-active in having casual sex partners, the infectious state of the individual, and the partner's infectious state. We will study the fraction of the population belonging to each type, each individual will therefore contribute with $1/n$ to the type it belongs to.

The fraction of all individuals that are susceptible, single and r-active is denoted by S_0^r; the fraction of all individuals that are single, r-active, and infectious in the acute stage is denoted by A_0^r; the fraction of all individuals that are single, r-active, and infectious in the chronic stage is denoted by C_0^r; and recovered singles (on ART-treatment) that are r-active is denoted by T_0^r.

Let $\mathcal{X} = \{S, A, C, T\}$ be the set of possible states not including PrEP, let $\mathcal{X_P} = \{SP, AP, CP\}$ be the possible states when being on PrEP, and let $\mathcal{D} = \{l, h\}$ be the set of possible activity degrees with regards to the casual contacts. Furthermore, let X_Y^{rq} denote the fraction of individuals that are r-active of type $X \in \mathcal{X} \cup \mathcal{X_P}$, with a q-active partner of type $Y \in \mathcal{X} \cup \mathcal{X_P}$. Note that, this counts each individual in the fraction X_Y^{rq}, not each pair. E.g. S_S^{rq} is the fraction of all individuals that are susceptible r-active and in a partnership with a q-active susceptible. The reason for taking this individual-based perspective is that the data is individual based. Moreover, the individual-based perspective makes it simpler to extend the model by allowing for more than one steady partner at a time in future work.

Disregarding the use of PrEP, there are in total 8 different single types and 64 types of partnerships (hence 72 equations). Some of these types' fraction must by consistency be equal, namely

- the 4 equations $X_X^{hl} = X_X^{lh}$ where $X \in \mathcal{X}$
- the 12 equations $X_S^{qr} = S_X^{rq}$ where $X \in \{A, C, T\}$ and $q, r \in \mathcal{D}$,
- the 8 equations $X_A^{qr} = A_X^{rq}$ where $X \in \{C, T\}$ and $q, r \in \mathcal{D}$,
- the 4 equations $T_C^{qr} = C_T^{rq}$ where $q, r \in \mathcal{D}$,

which reduces the number of equations to 44.

Including the use of PrEP for high-actives creates: 3 more single types (denoted SP_0^h, AP_0^h, and CP_0^h); 48 partnership types between one participant on PrEP and one not on PrEP; and 9 partnership types where both participants in the steady partnership are on PrEP. Hence, introducing PrEP increases the number of types by 60; however, some of the PrEP types' fraction must also by consistency be equal

- the 8 equations $X_{SP}^{qh} = SP_X^{hq}$ where $X \in \mathcal{X}$ and $q \in \mathcal{D}$,
- the 8 equations $X_{AP}^{qh} = AP_X^{hq}$ where $X \in \mathcal{X}$ and $q \in \mathcal{D}$,
- the 8 equations $X_{CP}^{qh} = CP_X^{hq}$ where $X \in \mathcal{X}$ and $q \in \mathcal{D}$,
- $SP_{AP}^{hh} = AP_{SP}^{hh}$,
- $SP_{CP}^{hh} = CP_{SP}^{hh}$,
- $AP_{CP}^{hh} = CP_{AP}^{hh}$.

For peer review only - http://bmjopen.bmj.com/site/about/guidelines.xhtml

Introducing PrEP increases the number of equations needed to be specified by 33, from 44 to 77.

There are some additional facts that will reduce the number of equations. Recall that the fraction without a steady partner is denoted P_0, the fraction with a steady partner is denoted P_1, and the fraction high-active individuals and low-active individuals in the population are denoted π_h and π_l, respectively. The following two constrains concerning singles must hold:

$$\begin{aligned}\pi_l P_0 &= S_0^l + A_0^l + C_0^l + T_0^l,\\ \pi_h P_0 &= S_0^h + SP_0^h + A_0^h + AP_0^h + C_0^h + CP_0^h + T_0^h.\end{aligned} \tag{S5.1}$$

The following three constrains for individuals in steady partnerships must hold. (I) The fraction of the population that is low-active in a steady partnership with a low-active is

$$\begin{aligned}\pi_l^2 P_1 &= S_S^{ll} + S_A^{ll} + S_C^{ll} + S_T^{ll}\\ &+ A_S^{ll} + A_A^{ll} + A_C^{ll} + A_T^{ll}\\ &+ C_S^{ll} + C_A^{ll} + C_C^{ll} + C_T^{ll}\\ &+ T_S^{ll} + T_A^{ll} + T_C^{ll} + T_T^{ll}\\ &= \sum_{X\in\mathcal{X}}\sum_{Y\in\mathcal{X}} X_Y^{ll}.\end{aligned}$$

That is, we sum over all possible states $X \in \mathcal{X}$, the first low-active individual in the relationship can have, and over all possible states that $Y \in \mathcal{X}$, the second low-active individual in the relationship can have. (II) The fraction of the population that is low-active in a steady partnership with a high-active is

$$\begin{aligned}\pi_l\pi_h P_1 &= S_S^{lh} + S_A^{lh} + S_C^{lh} + S_T^{lh} + S_{SP}^{lh} + S_{AP}^{lh} + S_{CP}^{lh}\\ &+ A_S^{lh} + A_A^{lh} + A_C^{lh} + A_T^{lh} + A_{SP}^{lh} + A_{AP}^{lh} + A_{CP}^{lh}\\ &+ C_S^{lh} + C_A^{lh} + C_C^{lh} + C_T^{lh} + C_{SP}^{lh} + C_{AP}^{lh} + C_{CP}^{lh}\\ &+ T_S^{lh} + T_A^{lh} + T_C^{lh} + T_T^{lh} + T_{SP}^{lh} + T_{AP}^{lh} + T_{CP}^{lh}\\ &= \sum_{X\in\mathcal{X}}\left(\sum_{Y\in\mathcal{X}\cup\mathcal{X}_\mathcal{P}} X_Y^{lh}\right),\end{aligned}$$

here we sum over all possible states $X \in \mathcal{X}$, the first low-active individual in the relationship can have, and over all possible states that $Y \in \mathcal{X}\cup\mathcal{X}_\mathcal{P}$, the second high-active individual in the relationship can have. The difference from the previous sum is that a high-active individual can be on PrEP. The fraction of the population that is high-active in a steady partnership with a low-active (which is the same as fraction low with high above) is

$$\pi_h\pi_l P_1 = \sum_{X\in\mathcal{X}\cup\mathcal{X}_\mathcal{P}}\left(\sum_{Y\in\mathcal{X}} X_Y^{hl}\right).$$

(III) The fraction of the population that is high-active in a steady partnership with a high-active is

$$\pi_h^2 P_1 = \sum_{X\in\mathcal{X}\cup\mathcal{X}_\mathcal{P}}\left(\sum_{Y\in\mathcal{X}\cup\mathcal{X}_\mathcal{P}} X_Y^{hh}\right).$$

This leads to that we can reduce the number of equations further, from 77 to 72.

Before we show the system of differential equations that describe our model, we define some quantities that will improve readability. Let us write the fraction of the population that is r-active susceptible in a partnership as S_1^r (where 1 refer to having a steady partner),

$$\begin{aligned}S_1^r &= S_{SP}^{rh} + S_{AP}^{rh} + S_{CP}^{rh} + S_S^{rh} + S_A^{rh} + S_C^{rh} + S_T^{rh} + S_S^{rl} + S_A^{rl} + S_C^{rl} + S_T^{rl}\\ &= S_{SP}^{rh} + S_{AP}^{rh} + S_{CP}^{rh} + \sum_{q\in\mathcal{D}} S_S^{rq} + S_A^{rq} + S_C^{rq} + S_T^{rq}\\ &= \sum_{X\in\mathcal{X}_\mathcal{P}} S_X^{rh} + \sum_{X\in\mathcal{X}}\sum_{q\in\mathcal{D}} S_X^{rq}.\end{aligned}$$

For peer review only - http://bmjopen.bmj.com/site/about/guidelines.xhtml

Similarly, we write the fraction r-active acute infectious individuals in a partnership as A_1^r,

$$A_1^r = \sum_{X \in \mathcal{X}_\mathcal{P}} A_X^{rh} + \sum_{X \in \mathcal{X}} \sum_{q \in \mathcal{D}} A_X^{rq};$$

the fraction r-active chronic infectious individuals in a partnership as C_1^r,

$$C_1^r = \sum_{X \in \mathcal{X}_\mathcal{P}} C_X^{rh} + \sum_{X \in \mathcal{X}} \sum_{q \in \mathcal{D}} C_X^{rq};$$

and the fraction r-active diagnosed and on ART-treatment in a partnership as T_1^r,

$$T_1^r = \sum_{X \in \mathcal{X}_\mathcal{P}} T_X^{rh} + \sum_{X \in \mathcal{X}} \sum_{q \in \mathcal{D}} T_X^{rq}.$$

For the PrEP states we have

$$\begin{aligned}
SP_1^h &= \sum_{X \in \mathcal{X}_\mathcal{P}} SP_X^{hh} + \sum_{X \in \mathcal{X}} \sum_{q \in \mathcal{D}} SP_X^{hq},\\
AP_1^h &= \sum_{X \in \mathcal{X}_\mathcal{P}} AP_X^{hh} + \sum_{X \in \mathcal{X}} \sum_{q \in \mathcal{D}} AP_X^{hq},\\
CP_1^h &= \sum_{X \in \mathcal{X}_\mathcal{P}} CP_X^{hh} + \sum_{X \in \mathcal{X}} \sum_{q \in \mathcal{D}} CP_X^{hq}.
\end{aligned}$$

Remember that the transmission probability in one sex act in the acute stage is denoted by p_A and in the chronic stage by p_C. An r-active individual who is single, susceptible, and not on PrEP acquire infection at rate

$$\begin{aligned}
\beta_0^r = {} & \alpha_{00}^{rh} \left(p_A(A_0^h + AP_0^h) + p_C(C_0^h + CP_0^h) \right)\\
& + \alpha_{01}^{rh} \left(p_A(A_1^h + AP_1^h) + p_C(C_1^h + CP_1^h) \right)\\
& + \alpha_{00}^{rl} (p_A A_0^l + p_C C_0^l)\\
& + \alpha_{01}^{rl} (p_A A_1^l + p_C C_1^l).
\end{aligned}$$

Similarly, an r-active susceptible not on PrEP with a steady partner acquire infection via casual sex at rate

$$\begin{aligned}
\beta_1^r = {} & \alpha_{10}^{rh} \left(p_A(A_0^h + AP_0^h) + p_C(C_0^h + CP_0^h) \right)\\
& + \alpha_{11}^{rh} \left(p_A(A_1^h + AP_1^h) + p_C(C_1^h + CP_1^h) \right)\\
& + \alpha_{10}^{rl} (p_A A_0^l + p_C C_0^l)\\
& + \alpha_{11}^{rl} (p_A A_1^l + p_C C_1^l).
\end{aligned}$$

Introducing PrEP will make some susceptible less likely to acquire infection, let us denote the reduction by ζ. Assuming a susceptible is protected by 86% by PrEP yields a value of $\zeta = 1 - 0.86 = 0.14$. A high-active susceptible that is single and on PrEP will acquire infection via casual sex at rate $\zeta \beta_0^h$.

The model can now be described by a set of 72 differential equations. We will begin by specifying all single state equations, followed by the different partnership state equations. We remind the reader of the parameter definitions that can be found in Table S1.1.

S5.1 Single states

For high-active susceptible singles not on PrEP we have that

$$\frac{dS_0^h}{dt} = \overbrace{\mu \pi_h}^{\substack{\text{Birth of}\\ \text{high-active}}} + \underbrace{(\sigma + \mu) S_1^h}_{\substack{\text{Separation of high-active}\\ \text{from a partner}}} - \overbrace{(\xi + \mu + \rho P_0) S_0^h}^{\substack{\text{High-active single starts PrEP,}\\ \text{dies or enter partnership}}} - \underbrace{\beta_0^h S_0^h}_{\substack{\text{High-active single}\\ \text{acquire infection}}}.$$

For the other single states, we have

$$\frac{dSP_0^h}{dt} = \xi S_0^h + (\sigma+\mu)SP_1^h - (\mu+\rho P_0)SP_0^h - \zeta\beta_0^h SP_0^h,$$
$$\frac{dA_0^h}{dt} = (\sigma+\mu)A_1^h - (\mu+\gamma_h+\delta_a+\rho P_0)A_0^h + \beta_0^h S_0^h,$$
$$\frac{dAP_0^h}{dt} = (\sigma+\mu)AP_1^h - (\mu+\gamma_P+\delta_a+\rho P_0)AP_0^h + \zeta\beta_0^h SP_0^h,$$
$$\frac{dC_0^h}{dt} = (\sigma+\mu)C_1^h + \delta_a A_0^h - (\mu+\gamma_h+\rho P_0)C_0^h,$$
$$\frac{dCP_0^h}{dt} = (\sigma+\mu)CP_1^h + \delta_a AP_0^h - (\mu+\gamma_P+\rho P_0)CP_0^h.$$

Due to constrain (S5.1), the fraction of the population that is high-active on ART-treatment is equal to

$$T_0^h = \pi_h P_0 - \left(S_0^h + SP_0^h + A_0^h + AP_0^h + C_0^h + CP_0^h\right).$$

In a similar way we get the equations for a low-active single. Note that a low-active never starts to use PrEP; however, we could switch which activity-group is targeted for the intervention.

$$\frac{dS_0^l}{dt} = \mu\pi_l + (\sigma+\mu)S_1^l - (\mu+\rho P_0)S_0^l - \beta_0^l S_0^l,$$
$$\frac{dA_0^l}{dt} = (\sigma+\mu)A_1^l - (\mu+\gamma_h+\delta_a+\rho P_0)A_0^l + \beta_0^l S_0^l,$$
$$\frac{dC_0^l}{dt} = (\sigma+\mu)C_1^l + \delta_a A_0^l - (\mu+\gamma_h+\rho P_0)C_0^l.$$

And the fraction low-active on ART-treatment is equal to $T_0^l = \pi_l P_0 - \left(S_0^l + A_0^l + C_0^l\right)$.

S5.2 Partnership states

We will specify the partnership state equations in the following order: first all possible combinations of a susceptible not on PrEP, S_S, S_A, S_C, S_T, S_{SP}, S_{AP}, S_{CP}; then all possible combinations of an acute infectious individual not on PrEP that has not previously been specified, A_A, A_C, A_T, A_{SP}, A_{AP}, A_{CP}; then all possible combinations of a chronic infectious individual not on PrEP that has not previously been specified, C_C, C_T, C_{SP}, C_{AP}, C_{CP}; then the treated individuals not previously specified, T_T, T_{SP}, T_{AP}, T_{CP}. Then we specify the partnership type equations of individuals on PrEP: for a susceptible on PrEP, SP_{SP}, SP_{AP}, SP_{CP}; for an acute infectious on PrEP, AP_{AP}, AP_{CP}; and finally for a chronic infectious on PrEP, CP_{CP}.

S5.2.1 Susceptible not on PrEP

For susceptible individuals not on PrEP in a partnership with another susceptible not on PrEP we have that

$$\frac{dS_S^{hh}}{dt} = \rho\left(S_0^h\right)^2 - (2\xi+\sigma+2\mu)S_S^{hh} - 2\beta_1^h S_S^{hh},$$
$$\frac{dS_S^{hl}}{dt} = \rho S_0^h S_0^l - (\xi+\sigma+2\mu)S_S^{hl} - (\beta_1^h+\beta_1^l)S_S^{hl},$$
$$\frac{dS_S^{ll}}{dt} = \rho\left(S_0^l\right)^2 - (\sigma+2\mu)S_S^{ll} - 2\beta_1^l S_S^{ll},$$

where $S_S^{lh} = S_S^{hl}$.

For peer review only - http://bmjopen.bmj.com/site/about/guidelines.xhtml
For peer review only

For susceptible individuals not on PrEP in a partnership with an acute infectious individual not on PrEP

$$\frac{dS_A^{hh}}{dt} = \rho S_0^h A_0^h + \beta_1^h S_S^{hh} - (\xi + \lambda p_A + \delta_a + \gamma_h + \sigma + 2\mu) S_A^{hh} - \beta_1^h S_A^{hh},$$
$$\frac{dS_A^{hl}}{dt} = \rho S_0^h A_0^l + \beta_1^l S_S^{hl} - (\xi + \lambda p_A + \delta_a + \gamma_l + \sigma + 2\mu) S_A^{hl} - \beta_1^h S_A^{hl},$$
$$\frac{dS_A^{lh}}{dt} = \rho S_0^l A_0^h + \beta_1^h S_S^{lh} - (\lambda p_A + \delta_a + \gamma_h + \sigma + 2\mu) S_A^{lh} - \beta_1^l S_A^{lh},$$
$$\frac{dS_A^{ll}}{dt} = \rho S_0^l A_0^l + \beta_1^l S_S^{ll} - (\lambda p_A + \delta_a + \gamma_l + \sigma + 2\mu) S_A^{ll} - \beta_1^l S_A^{ll}.$$

Note that $A_S^{rq} = S_A^{qr}$.

For susceptible individuals not on PrEP in a partnership with a chronic infectious individual not on PrEP

$$\frac{dS_C^{hh}}{dt} = \rho S_0^h C_0^h + \delta_a S_A^{hh} - (\xi + \lambda p_C + \gamma_h + \sigma + 2\mu) S_C^{hh} - \beta_1^h S_C^{hh}$$
$$\frac{dS_C^{hl}}{dt} = \rho S_0^h C_0^l + \delta_a S_A^{hl} - (\xi + \lambda p_C + \gamma_l + \sigma + 2\mu) S_C^{hl} - \beta_1^h S_C^{hl},$$
$$\frac{dS_C^{lh}}{dt} = \rho S_0^l C_0^h + \delta_a S_A^{lh} - (\lambda p_C + \gamma_h + \sigma + 2\mu) S_C^{lh} - \beta_1^l S_C^{lh},$$
$$\frac{dS_C^{ll}}{dt} = \rho S_0^l C_0^l + \delta_a S_A^{ll} - (\lambda p_C + \gamma_l + \sigma + 2\mu) S_C^{ll} - \beta_1^l S_C^{ll}.$$

And additionally, $C_S^{rq} = S_C^{qr}$.

For susceptible individuals not on PrEP in a partnership with an individual on ART-treatment

$$\frac{dS_T^{hh}}{dt} = \rho S_0^h T_0^h + \gamma_h (S_A^{hh} + S_C^{hh}) + \gamma_P (S_{AP}^{hh} + S_{CP}^{hh}) - (\xi + \sigma + 2\mu) S_T^{hh} - \beta_1^h S_T^{hh},$$
$$\frac{dS_T^{hl}}{dt} = \rho S_0^h T_0^l + \gamma_l (S_A^{hl} + S_C^{hl}) - (\xi + \sigma + 2\mu) S_T^{hl} - \beta_1^h S_T^{hl},$$
$$\frac{dS_T^{lh}}{dt} = \rho S_0^l T_0^h + \gamma_h (S_A^{lh} + S_C^{lh}) + \gamma_P (S_{AP}^{lh} + S_{CP}^{lh}) - (\sigma + 2\mu) S_T^{lh} - \beta_1^l S_T^{lh},$$
$$\frac{dS_T^{ll}}{dt} = \rho S_0^l T_0^l + \gamma_l (S_A^{ll} + S_C^{ll}) - (\sigma + 2\mu) S_T^{ll} - \beta_1^l S_T^{ll}.$$

We also have that $T_S^{rq} = S_T^{qr}$.

For susceptible individuals not on PrEP with a steady susceptible partner on PrEP we have

$$\frac{dS_{SP}^{hh}}{dt} = \rho SP_0^h S_0^h + \xi S_S^{hh} - (\xi + \sigma + 2\mu) S_{SP}^{hh} - (\beta_1^h + \zeta \beta_1^h) S_{SP}^{hh},$$
$$\frac{dS_{SP}^{lh}}{dt} = \rho SP_0^h S_0^l + \xi S_S^{lh} - (\sigma + 2\mu) S_{SP}^{lh} - (\beta_1^l + \zeta \beta_1^h) S_{SP}^{lh}.$$

Note that $SP_S^{hh} = S_{SP}^{hh}$ and $SP_S^{hl} = S_{SP}^{lh}$.

For susceptible individuals not on PrEP in a partnership with an acute infectious individual on PrEP

$$\frac{dS_{AP}^{hh}}{dt} = \rho S_0^h AP_0^h + \zeta \beta_1^h S_{SP}^{hh} - (\xi + \lambda p_A + \delta_a + \gamma_P + \sigma + 2\mu) S_{AP}^{hh} - \beta_1^h S_{AP}^{hh},$$
$$\frac{dS_{AP}^{lh}}{dt} = \rho S_0^l AP_0^h + \zeta \beta_1^h S_{SP}^{lh} - (\lambda p_A + \delta_a + \gamma_P + \sigma + 2\mu) S_{AP}^{lh} - \beta_1^l S_{AP}^{lh}.$$

Note that $AP_S^{hh} = S_{AP}^{hh}$ and $AP_S^{hl} = S_{AP}^{lh}$.

For susceptible individuals not on PrEP in a partnership with a chronic infectious individual on PrEP

$$\frac{dS_{CP}^{hh}}{dt} = \rho S_0^h CP_0^h + \delta_a S_{AP}^{hh} - (\xi + \lambda p_C + \gamma_P + \sigma + 2\mu) S_{CP}^{hh} - \beta_1^h S_{CP}^{hh}$$
$$\frac{dS_{CP}^{lh}}{dt} = \rho S_0^l CP_0^h + \delta_a S_{AP}^{lh} - (\lambda p_C + \gamma_P + \sigma + 2\mu) S_{CP}^{lh} - \beta_1^l S_{CP}^{lh}.$$

For peer review only - http://bmjopen.bmj.com/site/about/guidelines.xhtml

Note that $CP_S^{hh} = S_{CP}^{hh}$ and $CP_S^{hl} = S_{CP}^{lh}$.

S5.2.2 Acute infectious individuals not on PrEP

For acute infectious individuals not on PrEP in a partnership with another acute infectious not on PrEP we have

$$\begin{aligned}
\frac{dA_A^{hh}}{dt} &= \rho \left(A_0^h\right)^2 - (\sigma + 2\mu + 2\gamma_h + 2\delta_a)A_A^{hh} + 2\left(\lambda p_A + \beta_1^h\right) S_A^{hh},\\
\frac{dA_A^{hl}}{dt} &= \rho A_0^h A_0^l - (\sigma + 2\mu + \gamma_h + \gamma_l + 2\delta_a)A_A^{hl} + (\lambda p_A + \beta_1^h)S_A^{hl} + (\lambda p_A + \beta_1^l)A_S^{hl},\\
\frac{dA_A^{ll}}{dt} &= \rho \left(A_0^l\right)^2 - (\sigma + 2\mu + 2\gamma_l + 2\delta_a)A_A^{ll} + 2\left(\lambda p_A + \beta_1^l\right) S_A^{ll}.
\end{aligned}$$

where additionally $A_A^{lh} = A_A^{hl}$.

For acute infectious individuals in a partnership with a chronic infectious individual

$$\begin{aligned}
\frac{dA_C^{hh}}{dt} &= \rho A_0^h C_0^h + \delta_a A_A^{hh} - (\sigma + 2\mu + 2\gamma_h + \delta_a)A_C^{hh} + \left(\lambda p_C + \beta_1^h\right) S_C^{hh},\\
\frac{dA_C^{hl}}{dt} &= \rho A_0^h C_0^l + \delta_a A_A^{hl} - (\sigma + 2\mu + \gamma_h + \gamma_l + \delta_a)A_C^{hl} + \left(\lambda p_C + \beta_1^h\right) S_C^{hl},\\
\frac{dA_C^{lh}}{dt} &= \rho A_0^l C_0^h + \delta_a A_A^{lh} - (\sigma + 2\mu + \gamma_h + \gamma_l + \delta_a)A_C^{lh} + \left(\lambda p_C + \beta_1^l\right) S_C^{lh},\\
\frac{dA_C^{ll}}{dt} &= \rho A_0^l C_0^l + \delta_a A_A^{ll} - (\sigma + 2\mu + 2\gamma_l + \delta_a)A_C^{ll} + \left(\lambda p_C + \beta_1^l\right) S_C^{ll}.
\end{aligned}$$

Note that $C_A^{rq} = A_C^{qr}$.

For acute infectious individuals in a partnership with an individual on ART-treatment

$$\begin{aligned}
\frac{dA_T^{hh}}{dt} &= \rho A_0^h T_0^h + \gamma_h(A_C^{hh} + A_A^{hh}) + \gamma_P(A_{CP}^{hh} + A_{AP}^{hh}) - (\gamma_h + \sigma + 2\mu + \delta_a)A_T^{hh} + \beta_1^h S_T^{hh}\\
\frac{dA_T^{hl}}{dt} &= \rho A_0^h T_0^l + \gamma_l(A_C^{hl} + A_A^{hl}) - (\gamma_h + \sigma + 2\mu + \delta_a)A_T^{hl} + \beta_1^h S_T^{hl},\\
\frac{dA_T^{lh}}{dt} &= \rho A_0^l T_0^h + \gamma_h(A_C^{lh} + A_A^{lh}) + \gamma_P(A_{CP}^{lh} + A_{AP}^{lh}) - (\gamma_l + \sigma + 2\mu + \delta_a)A_T^{lh} + \beta_1^l S_T^{lh},\\
\frac{dA_T^{ll}}{dt} &= \rho A_0^l T_0^l + \gamma_l(A_C^{ll} + A_A^{ll}) - (\gamma_l + \sigma + 2\mu + \delta_a)A_T^{ll} + \beta_1^l S_T^{ll}.
\end{aligned}$$

Also, $T_A^{rq} = A_T^{qr}$.

For acute infectious individuals not on PrEP in a partnership with a susceptible individual on PrEP

$$\begin{aligned}
\frac{dA_{SP}^{hh}}{dt} &= \rho A_0^h SP_0^h + \xi A_S^{hh} + \beta_1^h S_{SP}^{hh} - (\zeta\lambda p_A + \delta_a + \gamma_h + \sigma + 2\mu)A_{SP}^{hh} - \zeta\beta_1^h A_{SP}^{hh},\\
\frac{dA_{SP}^{lh}}{dt} &= \rho A_0^l SP_0^h + \xi A_S^{lh} + \beta_1^l S_{SP}^{lh} - (\zeta\lambda p_A + \delta_a + \gamma_l + \sigma + 2\mu)A_{SP}^{lh} - \zeta\beta_1^h A_{SP}^{lh}.
\end{aligned}$$

Note that $SP_A^{hh} = A_{SP}^{hh}$ and $SP_A^{hl} = A_{SP}^{lh}$.

For acute infectious individuals not on PrEP in a partnership with an acute infectious individual on PrEP

$$\begin{aligned}
\frac{dA_{AP}^{hh}}{dt} &= \rho A_0^h AP_0^h - (\sigma + 2\mu + \gamma_h + \gamma_P + 2\delta_a)A_{AP}^{hh} + (\lambda p_A + \beta_1^h)S_{AP}^{hh} + (\zeta\lambda p_A + \zeta\beta_1^h)A_{SP}^{hh},\\
\frac{dA_{AP}^{lh}}{dt} &= \rho A_0^l AP_0^h - (\sigma + 2\mu + \gamma_l + \gamma_P + 2\delta_a)A_{AP}^{lh} + (\lambda p_A + \beta_1^l)S_{AP}^{lh} + (\zeta\lambda p_A + \zeta\beta_1^h)A_{SP}^{lh}.
\end{aligned}$$

For peer review only - http://bmjopen.bmj.com/site/about/guidelines.xhtml
For peer review only

Note that $AP_A^{hh} = A_{AP}^{hh}$ and $AP_A^{hl} = A_{AP}^{lh}$.

For acute infectious individuals not on PrEP in a partnership with a chronic infectious individual on PrEP

$$\frac{dA_{CP}^{hh}}{dt} = \rho A_0^h CP_0^h + \delta_a A_{AP}^{hh} - (\sigma + 2\mu + \gamma_h + \gamma_P + \delta_a) A_{CP}^{hh} + (\lambda p_C + \beta_1^h) S_{CP}^{hh},$$
$$\frac{dA_{CP}^{lh}}{dt} = \rho A_0^l CP_0^h + \delta_a A_{AP}^{lh} - (\sigma + 2\mu + \gamma_l + \gamma_P + \delta_a) A_{CP}^{lh} + (\lambda p_C + \beta_1^l) S_{CP}^{lh}.$$

Note that $CP_A^{hh} = A_{CP}^{hh}$ and $CP_A^{hl} = A_{CP}^{lh}$.

S5.2.3 Chronic infectious not on PrEP

For an individual with a chronic infection not on PrEP in a steady partnership with another individual with a chronic infection

$$\frac{dC_C^{hh}}{dt} = \rho \left(C_0^h\right)^2 + \delta_a (A_C^{hh} + C_A^{hh}) - (\sigma + 2\mu + 2\gamma_h) C_C^{hh},$$
$$\frac{dC_C^{hl}}{dt} = \rho C_0^h C_0^l + \delta_a (A_C^{hl} + C_A^{hl}) - (\sigma + 2\mu + \gamma_h + \gamma_l) C_C^{hl}$$
$$\frac{dC_C^{ll}}{dt} = \rho \left(C_0^l\right)^2 + \delta_a (A_C^{ll} + C_A^{ll}) - (\sigma + 2\mu + 2\gamma_l) C_C^{ll}.$$

where $C_C^{lh} = C_C^{hl}$.

For an individual with a chronic infection not on PrEP in a steady partnership with an individual under ART-treatment

$$\frac{dC_T^{hh}}{dt} = \rho C_0^h T_0^h + \delta_a A_T^{hh} + \gamma_h (C_A^{hh} + C_C^{hh}) + \gamma_P (C_{AP}^{hh} + C_{CP}^{hh}) - (\sigma + 2\mu + \gamma_h) C_T^{hh},$$
$$\frac{dC_T^{hl}}{dt} = \rho C_0^h T_0^l + \delta_a A_T^{hl} + \gamma_l (C_A^{hl} + C_C^{hl}) - (\sigma + 2\mu + \gamma_h) C_T^{hl},$$
$$\frac{dC_T^{lh}}{dt} = \rho C_0^l T_0^h + \delta_a A_T^{lh} + \gamma_h (C_A^{lh} + C_C^{lh}) + \gamma_P (C_{AP}^{lh} + C_{CP}^{lh}) - (\sigma + 2\mu + \gamma_l) C_T^{lh},$$
$$\frac{dC_T^{ll}}{dt} = \rho C_0^l T_0^l + \delta_a A_T^{ll} + \gamma_l (C_A^{ll} + C_C^{ll}) - (\sigma + 2\mu + \gamma_l) C_T^{ll}.$$

where $T_C^{rq} = C_T^{qr}$.

For an individual with a chronic infection not on PrEP in a partnership with a susceptible individual on PrEP

$$\frac{dC_{SP}^{hh}}{dt} = \rho C_0^h SP_0^h + \xi C_S^{hh} + \delta_a A_{SP}^{hh} - (\zeta \lambda p_C + \gamma_h + \sigma + 2\mu) C_{SP}^{hh} - \zeta \beta_1^h C_{SP}^{hh}$$
$$\frac{dC_{SP}^{lh}}{dt} = \rho C_0^l SP_0^h + \xi C_S^{lh} + \delta_a A_{SP}^{lh} - (\zeta \lambda p_C + \gamma_l + \sigma + 2\mu) C_{SP}^{lh} - \zeta \beta_1^h C_{SP}^{lh}.$$

And additionally $SP_C^{hh} = C_{SP}^{hh}$ and $SP_C^{hl} = C_{SP}^{lh}$.

For an individual with a chronic infection not on PrEP in a partnership with an acute infectious individual on PrEP

$$\frac{dC_{AP}^{hh}}{dt} = \rho C_0^h AP_0^h + \delta_a A_{AP}^{hh} - (\sigma + 2\mu + \gamma_h + \gamma_P + \delta_a) C_{AP}^{hh} + (\zeta \lambda p_C + \zeta \beta_1^h) C_{SP}^{hh},$$
$$\frac{dC_{AP}^{lh}}{dt} = \rho C_0^l AP_0^h + \delta_a A_{AP}^{lh} - (\sigma + 2\mu + \gamma_l + \gamma_P + \delta_a) C_{AP}^{lh} + (\zeta \lambda p_C + \zeta \beta_1^h) C_{SP}^{lh}.$$

Note that $AP_C^{hh} = C_{AP}^{hh}$ and $AP_C^{hl} = C_{AP}^{lh}$.

For peer review only - http://bmjopen.bmj.com/site/about/guidelines.xhtml

For an individual with a chronic infection not on PrEP in a partnership with a chronic infectious individual on PrEP

$$\frac{dC^{hh}_{CP}}{dt} = \rho C^h_0 CP^h_0 + \delta_a(A^{hh}_{CP} + C^{hh}_{AP}) - (\sigma + 2\mu + \gamma_h + \gamma_P)C^{hh}_{CP},$$
$$\frac{dC^{lh}_{CP}}{dt} = \rho C^l_0 CP^h_0 + \delta_a(A^{lh}_{CP} + C^{lh}_{AP}) - (\sigma + 2\mu + \gamma_l + \gamma_P)C^{lh}_{CP}.$$

where $CP^{hh}_C = C^{hh}_{CP}$ and $CP^{hl}_C = C^{lh}_{CP}$.

S5.2.4 Treated individual

For an individual under ART-treatment in a steady partnership with another individual under ART-treatment we have

$$\frac{dT^{hh}_T}{dt} = \rho(T^h_0)^2 + \gamma_h(A^{hh}_T + C^{hh}_T + T^{hh}_A + T^{hh}_C) + \gamma_P(AP^{hh}_T + CP^{hh}_T + T^{hh}_{AP} + T^{hh}_{CP}) - (\sigma + 2\mu)T^{hh}_T,$$
$$\frac{dT^{hl}_T}{dt} = \rho T^h_0 T^l_0 + \gamma_h(A^{hl}_T + C^{hl}_T) + \gamma_l(T^{hl}_A + T^{hl}_C) + \gamma_P(AP^{hl}_T + CP^{hl}_T) - (\sigma + 2\mu)T^{hl}_T$$
$$\frac{dT^{ll}_T}{dt} = \rho(T^l_0)^2 + \gamma_l(A^{ll}_T + C^{ll}_T) + \gamma_l(T^{ll}_A + T^{ll}_C) - (\sigma + 2\mu)T^{ll}_T.$$

where $T^{lh}_T = T^{hl}_T$.

For treated individuals in a partnership with a susceptible individual on PrEP

$$\frac{dT^{hh}_{SP}}{dt} = \rho T^h_0 SP^h_0 + \xi T^{hh}_S + \gamma_h(A^{hh}_{SP} + C^{hh}_{SP}) + \gamma_P(AP^{hh}_{SP} + CP^{hh}_{SP}) - (\sigma + 2\mu)T^{hh}_{SP} - \zeta\beta^h_1 T^{hh}_{SP},$$
$$\frac{dT^{lh}_{SP}}{dt} = \rho T^l_0 SP^h_0 + \xi T^{lh}_S + \gamma_l(A^{lh}_{SP} + C^{lh}_{SP}) - (\sigma + 2\mu)T^{lh}_{SP} - \zeta\beta^h_1 T^{lh}_{SP}.$$

We also have that $SP^{hh}_T = T^{hh}_{SP}$ and $SP^{hl}_T = T^{lh}_{SP}$.

For treated individuals in a partnership with an acute infectious individual on PrEP

$$\frac{dT^{hh}_{AP}}{dt} = \rho T^h_0 AP^h_0 + \gamma_h(C^{hh}_{AP} + A^{hh}_{AP}) + \gamma_P(AP^{hh}_{AP} + CP^{hh}_{AP}) - (\gamma_P + \sigma + 2\mu + \delta_a)T^{hh}_{AP} + \zeta\beta^h_1 T^{hh}_{SP}$$
$$\frac{dT^{lh}_{AP}}{dt} = \rho T^l_0 AP^h_0 + \gamma_l(C^{lh}_{AP} + A^{lh}_{AP}) - (\gamma_P + \sigma + 2\mu + \delta_a)T^{lh}_{AP} + \zeta\beta^h_1 T^{lh}_{SP}.$$

Also, $AP^{hh}_T = T^{hh}_{AP}$ and $AP^{hl}_T = T^{lh}_{AP}$.

For treated individuals in a partnership with a chronic infectious individual on PrEP

$$\frac{dT^{hh}_{CP}}{dt} = \rho T^h_0 CP^h_0 + \delta_a T^{hh}_{AP} + \gamma_h(A^{hh}_{CP} + C^{hh}_{CP}) + \gamma_P(AP^{hh}_{CP} + CP^{hh}_{CP}) - (\sigma + 2\mu + \gamma_P)T^{hh}_{CP},$$
$$\frac{dT^{lh}_{CP}}{dt} = \rho T^l_0 CP^h_0 + \delta_a T^{lh}_{AP} + \gamma_l(A^{lh}_{CP} + C^{lh}_{CP}) - (\sigma + 2\mu + \gamma_P)T^{lh}_{CP}.$$

where $CP^{hh}_T = T^{hh}_{CP}$ and $CP^{hl}_T = T^{lh}_{CP}$.

S5.2.5 Individuals on PrEP

For susceptible individuals on PrEP with a steady partner on PrEP we have

$$\frac{dSP^{hh}_{SP}}{dt} = \rho(SP^h_0)^2 + 2\xi SP^{hh}_S - (\sigma + 2\mu)SP^{hh}_{SP} - 2\zeta\beta^h_1 SP^{hh}_{SP},$$
$$\frac{dSP^{hh}_{AP}}{dt} = \rho SP^h_0 AP^h_0 + \xi S^{hh}_{AP} + \zeta\beta^h_1 SP^{hh}_{SP} - (\zeta\lambda p_A + \delta_a + \gamma_P + \sigma + 2\mu)SP^{hh}_{AP} - \zeta\beta^h_1 SP^{hh}_{AP},$$
$$\frac{dSP^{hh}_{CP}}{dt} = \rho SP^h_0 CP^h_0 + \xi S^{hh}_{CP} + \delta_a SP^{hh}_{AP} - (\zeta\lambda p_C + \gamma_P + \sigma + 2\mu)SP^{hh}_{CP} - \zeta\beta^h_1 SP^{hh}_{CP}.$$

For peer review only - http://bmjopen.bmj.com/site/about/guidelines.xhtml

Note that $AP^{hh}_{SP} = SP^{hh}_{AP}$ and that $CP^{hh}_{SP} = SP^{hh}_{CP}$.

For acute infectious individuals on PrEP in a steady partnership with an individual on PrEP

$$\frac{dAP^{hh}_{AP}}{dt} = \rho(AP^h_0)^2 - (\sigma + 2\mu + 2\gamma_P + 2\delta_a)AP^{hh}_{AP} + 2(\zeta\lambda p_A + \zeta\beta^h_1)AP^{hh}_{SP},$$

$$\frac{dAP^{hh}_{CP}}{dt} = \rho AP^h_0 CP^h_0 + \delta_a AP^{hh}_{AP} - (\sigma + 2\mu + 2\gamma_P + \delta_a)AP^{hh}_{CP} + (\zeta\lambda p_C + \zeta\beta^h_1)SP^{hh}_{CP}.$$

Note that $CP^{hh}_{AP} = AP^{hh}_{CP}$.

And very much finally, for an individual with a chronic infection on PrEP in a partnership with a chronic infectious individual on PrEP we have

$$\frac{dCP^{hh}_{CP}}{dt} = \rho(CP^h_0)^2 + \delta_a(AP^{hh}_{CP} + CP^{hh}_{AP}) - (\sigma + 2\mu + 2\gamma_P)CP^{hh}_{CP}.$$

References

[S1] Leung KY, Kretzschmar M, and Diekmann O. SI infection on a dynamic partnership network: characterization of R_0. *Journal of Mathematical Biology*, 71(1):1–56, 2015.

[S2] D. Hansson, K.Y. Leung, T. Britton, and S. Strömdahl. A dynamic network model to disentangle the roles of steady and casual partners for HIV transmission among MSM. *Epidemics*, 2019. ISSN 1755-4365. doi: https://doi.org/10.1016/j.epidem.2019.02.001. URL http://www.sciencedirect.com/science/article/pii/S1755436518300963.

[S3] Tom Britton. Stochastic epidemic models: A survey. *Mathematical Biosciences*, 225(1):24 – 35, 2010. ISSN 0025-5564. doi: https://doi.org/10.1016/j.mbs.2010.01.006. URL http://www.sciencedirect.com/science/article/pii/S0025556410000143.

[S4] Hollingsworth TD, Anderson RM., and Fraser C. HIV-1 transmission, by stage of infection. *The Journal of Infectious Diseases*, 198(5):687, 2008. doi: 10.1086/590501.

[S5] Molina JM, Capitant C, Spire B, et al. On-Demand Preexposure Prophylaxis in Men at High Risk for HIV-1 Infection. *New England Journal of Medicine*, 373(23):2237–2246, 2015. doi: 10.1056/NEJMoa1506273. PMID: 26624850.

[S6] Leynaert B, Downs AM, de Vincenzi I., et al. Heterosexual transmission of human immunodeficiency virus variability of infectivity throughout the course of infection. *American journal of epidemiology*, 148(1):88–96, 1998.

[S7] Jin F, Jansson J, Law M, Prestage GP, Zablotska I, Imrie JCG, Kippax SC, Kaldor JM, Grulich AE, and Wilson DP. Per-contact probability of HIV transmission in homosexual men in Sydney in the era of HAART. *AIDS*, 24(6):907–913, 2010. doi: 10.1097/QAD.0b013e3283372d90.

For peer review only

Timeline follow-back tool

The TLFB tool was in Swedish and included one module concerning (1) sociodemographics, sexual identity, previous STI/HIV testing and diagnosis, and (2) a TLFB sexual behaviour module.
The TLFB tool was answered on an interactive tablet device. Here we will give the translated questions (from Swedish to English) and describe the tool. Descriptions of the tool is given in italics.

Sociodemographics, sexual identity, previous STI/HIV testing and diagnosis module

First the participant enters a page where they can answer a set of questions concerning themselves.

1. What year were you born?

2. Were you born in Sweden?
Yes (If yes, go to 2b-c)
No

2b. What country were you born in?
List of countries
Don't want to answer

2c. How many years have you been living in Sweden?
Box for number
Don't want to answer

3. What is your highest level of completed education?
No education
Elementary Grades 1-6
Elementary Grades 7-9
High school or equivalent
Non-university-post-high school education
Higher education/University
Doctorate
Other (Box for text)
Don't want to answer

4. What is your main occupation?
Full/Part time employment
Student
Self-employed
Job-seeking
Long-term sick leave
Sickness/activity leave
Retired
Other
Don't want to answer

For peer review only

5. What is your average income per month?
Less than 15000SEK
15000-25000SEK
25000-35000SEK
35000-45000SEK
More than 45000SEK
Don't want to answer

6. I identify myself as
Male
Female
Other
Don't want to answer

7. Are you or have you been a transperson?
Yes (Go to 7b & c)
No (If no, go to 8)
Don't want to answer

7b. Do you identify yourself as one or several of (you can answer several of the alternatives)?
Transperson
Transsexual
Former Transsexual
Transvestite
Transgender
Intergender
Intersexual
Other (box for text)
Don't want to answer

7c. Have you done a gender reassignment surgery?
Yes; Hormonal and Surgical
Yes; Hormonal
Yes; Surgical (partly or fully)
No
Don't want to answer

8. Do you identify yourself as one or several of (you can answer several of the alternatives)
Homosexual
Bisexual
Heterosexual
Other (Box for text)
Don't want to answer

9. What is your HIV status?
Negative
Positive (Go to question 9b, not 10)
Unsure/ I do not know
Don't want to answer

For peer review only

9b. When did you find out that you have HIV?
Year (Box for number)
Don't want to answer

10. When did you last get an HIV test
Less than 6 months
6 to 12 months
1 to 5 years
More than 5 years
Never
Don't remember
Don't want to answer

11. Have you had any of the following sexually transmitted infections the last 12 months?
No
Chlamydia
Gonorrhoea
Syphilis
Herpes
Condyloma/HPV/genital warts
Lymfogranuloma venerum (LGV)
Mycoplasma
Chancroid
Hepatitis A
Hepatitis B
Other
Don't want to answer

12. How many sex partners have you had the last 12 months?

Then the participants enter the TLFB module.

TLFB sexual behaviour module

Per partner (12 months)
Here we ask of you to report information on your 10 most recent sex partners during the last 12 months on a timeline. Begin with your latest sex partner and choose the alternative that best match your relationship.
Type of sex partner: which relationship do you have to your sex partner?

1. Main sex partner: A loving (emotional) relationship (e.g. boyfriend/girlfriend).
2. Regular sex partner (e.g. 'friends with benefits').
3. Casual known sex partner
4. Casual unknown sex partner

Upon answering this question the participant is brought to the timeline (represented by a green line).

Begin with marking the length of the relationship with an approximate starting and ending date within the green area. Then continue with the questions.

For peer review only

If the participant labelled their partner as steady (partner type 1 or 2), a line was marked on the timeline between the dates. If a participant labelled their partner as casual (partner type 3 or 4), an symbol X represented the casual sexual contact.

1. Question concerning all types of sex partners (sex partner of type 1-4)

- Gender of sex partner

Question:
Person is:
Male
Female
Trans
Other: (box for text)

2a. Questions concerning casual sex partner (that was marked with an X)

Question:
What type of sex did you have?
Anal sex – gave
Anal sex – received
Vaginal sex
Gave oral sex
Received oral sex
Other:

Condom use was given by ticking in one of the boxes 'condom' or 'no condom'

2b. Questions concerning steady sex partner (marked with a line)

Anal sex – gave – Approximately how many times a month? *Box for number*
Anal sex – received – Approximately how many times a month? *Box for number*
Vaginal sex – Approximately how many times a month? *Box for number*
Gave oral sex – Approximately how many times a month? *Box for number*
Received oral sex – Approximately how many times a month? *Box for number*

For each of the sex types the following was asked
How often did you use condom?
Always (100%)
Often (75%)
Half of the times (50%)
Seldom (25%)
Never (0%)

For peer review only

STROBE Statement—Checklist of items that should be included in reports of ***cross-sectional studies***

	Item No	Recommendation	Reported on page #
**Title and abstract**	1	(*a*) Indicate the study's design with a commonly used term in the title or the abstract	1
		(*b*) Provide in the abstract an informative and balanced summary of what was done and what was found	2-3
**Introduction**			
Background/rationale	2	Explain the scientific background and rationale for the investigation being reported	4-5
Objectives	3	State specific objectives, including any prespecified hypotheses	5
**Methods**			
Study design	4	Present key elements of study design early in the paper	5-6
Setting	5	Describe the setting, locations, and relevant dates, including periods of recruitment, exposure, follow-up, and data collection	9
Participants	6	(*a*) Give the eligibility criteria, and the sources and methods of selection of participants	9
Variables	7	Clearly define all outcomes, exposures, predictors, potential confounders, and effect modifiers. Give diagnostic criteria, if applicable	Supplementary material S3
Data sources/ measurement	8*	For each variable of interest, give sources of data and details of methods of assessment (measurement). Describe comparability of assessment methods if there is more than one group	
Bias	9	Describe any efforts to address potential sources of bias	
Study size	10	Explain how the study size was arrived at	
Quantitative variables	11	Explain how quantitative variables were handled in the analyses. If applicable, describe which groupings were chosen and why	Supplementary material S3
Statistical methods	12	(*a*) Describe all statistical methods, including those used to control for confounding	
		(*b*) Describe any methods used to examine subgroups and interactions	
		(*c*) Explain how missing data were addressed	
		(*d*) If applicable, describe analytical methods taking account of sampling strategy	
		(*e*) Describe any sensitivity analyses	
**Results**			
Participants	13*	(a) Report numbers of individuals at each stage of study—eg numbers potentially eligible, examined for eligibility, confirmed eligible, included in the study, completing follow-up, and analysed	9
		(b) Give reasons for non-participation at each stage	
		(c) Consider use of a flow diagram	
Descriptive data	14*	(a) Give characteristics of study participants (eg demographic, clinical, social) and information on exposures and potential confounders	Separate paper
		(b) Indicate number of participants with missing data for each variable of interest	9
Outcome data	15*	Report numbers of outcome events or summary measures	11
Main results	16	(*a*) Give unadjusted estimates and, if applicable, confounder-adjusted	12-14

For peer review only - http://bmjopen.bmj.com/site/about/guidelines.xhtml

		estimates and their precision (eg, 95% confidence interval). Make clear which confounders were adjusted for and why they were included	
		(*b*) Report category boundaries when continuous variables were categorized	
		(*c*) If relevant, consider translating estimates of relative risk into absolute risk for a meaningful time period	
Other analyses	17	Report other analyses done—eg analyses of subgroups and interactions, and sensitivity analyses	Supplemental material S4
**Discussion**			
Key results	18	Summarise key results with reference to study objectives	15-16
Limitations	19	Discuss limitations of the study, taking into account sources of potential bias or imprecision. Discuss both direction and magnitude of any potential bias	17
Interpretation	20	Give a cautious overall interpretation of results considering objectives, limitations, multiplicity of analyses, results from similar studies, and other relevant evidence	16
Generalisability	21	Discuss the generalisability (external validity) of the study results	17
**Other information**			
Funding	22	Give the source of funding and the role of the funders for the present study and, if applicable, for the original study on which the present article is based	18

*Give information separately for exposed and unexposed groups.

Note: An Explanation and Elaboration article discusses each checklist item and gives methodological background and published examples of transparent reporting. The STROBE checklist is best used in conjunction with this article (freely available on the Web sites of PLoS Medicine at http://www.plosmedicine.org/, Annals of Internal Medicine at http://www.annals.org/, and Epidemiology at http://www.epidem.com/). Information on the STROBE Initiative is available at www.strobe-statement.org.

For peer review only

Introducing pre-exposure prophylaxis to prevent HIV acquisition among men who have sex with men in Sweden: insights from a mathematical pair-formation model

Journal:	*BMJ Open*
Manuscript ID	bmjopen-2019-033852.R1
Article Type:	Original research
Date Submitted by the Author:	11-Dec-2019
Complete List of Authors:	Hansson, Disa; Stockholms Universitet Matematiska institutionen, mathematics<br>Strömdahl, Susanne; Uppsala University, Department of Medical Sciences; Karolinska Institute, Department of Public Health Sciences<br>Leung, Ka Yin; Stockholm University, Department of mathematics<br>Britton, Tom; Stockholms Universitet Matematiska institutionen, Mathematics
**Primary Subject Heading**:	HIV/AIDS
Secondary Subject Heading:	Public health, Sexual health
Keywords:	HIV & AIDS < INFECTIOUS DISEASES, Pre-exposure prophylaxis, Mathematical models, Transmission dynamics, Sexual networks

I, the Submitting Author has the right to grant and does grant on behalf of all authors of the Work (as defined in the below author licence), an exclusive licence and/or a non-exclusive licence for contributions from authors who are: i) UK Crown employees; ii) where BMJ has agreed a CC-BY licence shall apply, and/or iii) in accordance with the terms applicable for US Federal Government officers or employees acting as part of their official duties; on a worldwide, perpetual, irrevocable, royalty-free basis to BMJ Publishing Group Ltd ("BMJ") its licensees and where the relevant Journal is co-owned by BMJ to the co-owners of the Journal, to publish the Work in this journal and any other BMJ products and to exploit all rights, as set out in our licence.

The Submitting Author accepts and understands that any supply made under these terms is made by BMJ to the Submitting Author unless you are acting as an employee on behalf of your employer or a postgraduate student of an affiliated institution which is paying any applicable article publishing charge ("APC") for Open Access articles. Where the Submitting Author wishes to make the Work available on an Open Access basis (and intends to pay the relevant APC), the terms of reuse of such Open Access shall be governed by a Creative Commons licence – details of these licences and which Creative Commons licence will apply to this Work are set out in our licence referred to above.

Other than as permitted in any relevant BMJ Author's Self Archiving Policies, I confirm this Work has not been accepted for publication elsewhere, is not being considered for publication elsewhere and does not duplicate material already published. I confirm all authors consent to publication of this Work and authorise the granting of this licence.

For peer review only

Introducing pre-exposure prophylaxis to prevent HIV acquisition among men who have sex with men in Sweden: insights from a mathematical pair-formation model

Disa Hansson*[a] Susanne Strömdahl[b] KaYin Leung[a] Tom Britton[a]

Number of words: 3822

* Corresponding author. E-mail address: disa@math.su.se (D. Hansson). Phone number: +46704029803
[a] Department of Mathematics, Stockholm University, SE-106 91 Stockholm, Sweden
[b] Department of Medical Sciences, Section of Infectious Diseases, Uppsala University & Department of Public Health Sciences, Karolinska Institutet

For peer review only

Abstract

Objectives Since 2017, the Public health Agency of Sweden recommend that pre-exposure prophylaxis for HIV (PrEP) should be offered to high-risk individuals, in particular to men who have sex with men (MSM). The objective of this study is to develop a mathematical model investigating the effect of introducing PrEP to MSM in Sweden.

Design A pair-formation model, including steady and casual sex partners, is developed to study the impact of introducing PrEP. Two groups are included in the model: sexually high-active MSM and sexually low-active MSM. Three mixing assumptions between the groups are considered.

Setting A gay-friendly MSM HIV/STI-testing clinic in Stockholm, Sweden. This clinic started offering PrEP to MSM in October 2018.

Participants The model is calibrated according to detailed sexual behaviour data gathered in 2015 among 403 MSM.

Results By targeting sexually high-active MSM, a PrEP coverage of 3.5% of the MSM population (10% of all high-actives) would result in the long-term HIV prevalence to drop considerably (close to 0%). While targeting only low-actives would require a PrEP coverage of 35% for a similar reduction. The main effect of PrEP is the reduced susceptibility, whereas the increased HIV-testing rate (every 3rd month) among PrEP users plays a lesser role.

For peer review only

Conclusions To create a multifaceted picture of the effects of interventions against HIV, we need models that include the different stages of HIV infection and real-world data on detailed sexual behaviour to calibrate the mathematical models. Our findings conclude that targeting HIV high-risk individuals, within HIV risk populations such as MSM, with PrEP programmes could greatly decrease the long-term HIV prevalence in Sweden. Therefore, risk stratification of individuals is of importance in PrEP implementation programmes, to ensure optimising the effect and cost-effectiveness of such programmes.

Strengths and limitations of this study

- Using a mathematical pair-formation model we study the effect of introducing PrEP among MSM in Sweden, a group at high risk of HIV acquisition.
- The model divides the population into sexually high-active MSM and low-active MSM, where high-actives are offered to use PrEP.
- The model is calibrated to detailed sexual behavioural data gathered among the MSM population now being offered PrEP in Sweden.
- Limitations of this study include that the data only makes it possible to include two activity-groups, and that we do not allow for more than one steady sex partner at a time.

For peer review only

INTRODUCTION

In Sweden, the HIV prevalence was estimated to 0.07% in the general population in 2015,[1] whereas the self-reported HIV prevalence among men who have sex with men (MSM) has been estimated between 2% to 6%.[2] Sweden was the first country to report having achieved the UNAIDS/WHO 90-90-90 goal in 2016,[1] with at least 90% of people living with HIV being aware of their HIV status, 90% of HIV diagnosed individuals being on antiretroviral therapy (ART), and with 90% of those on ART being under viral suppression.[3] Viral suppression means achieving continuously undetectable HIV viral load that diminishes onward transmission to close to zero.[4,5]

As a result of ART's effectiveness in viral suppression it can be viewed as an effective preventive measure for further HIV transmission. However, on its own it does not seem to reduce HIV prevalence enough in risk-groups, such as the MSM population, but needs to be combined with additional preventive strategies.[6–8] One such preventive intervention is oral pre-exposure prophylaxis for HIV (PrEP), i.e. that the antiviral drugs tenofovir-emtricitabine are taken by individuals with negative HIV serostatus to prevent HIV acquisition.[9,10] PrEP effectiveness is dependent on adherence to PrEP to ensure that protective concentrations of the drugs are present at exposure to prevent transmission.[11] Two different studies report that PrEP reduces the HIV incidence by 86% among MSM (95% CI 40–98 and 90% CI 64–96 respectively).[9,10] Due to the effectiveness of PrEP, the World Health Organisation (WHO) recommend PrEP to be offered to individuals at substantial risk of HIV acquisition, defined as an HIV incidence of 3 or above per 100 person-years.[12]

For peer review only

The use of PrEP in Sweden was approved in 2016 by the Swedish Medical Products Agency.[13] Since 2017, the Public health Agency of Sweden recommend that PrEP should be offered to high-risk MSM. However, very few clinics started offering PrEP at this time due to logistical and funding concerns. Since June 2018, the New Therapies Council recommend Swedish counties to implement PrEP programmes for MSM and offer subsidised PrEP.[14] The larger gay-friendly sexual health clinics in the major three urban areas of Sweden have since then started to implement PrEP. As of July 2019, approximately 315 individuals have initiated PrEP in Stockholm (personal communication with Dr. FinnFilén responsible for PrEP at Venhälsan, Södersjukhuset).

The objective of this study is to investigate the effect of introducing PrEP to MSM in Sweden. First, we incorporate the use of PrEP into pair-formation models developed to study HIV transmission.[15] This model separates individuals depending on sexual activity-degree, high-active or low-active. The model is then fitted to sexual behaviour data from a gay-friendly HIV/STI-testing clinic in Stockholm, Sweden. Finally, the effect of PrEP on HIV transmission is studied by risk-stratifying MSM for PrEP, to explore the level of PrEP coverage needed to substantially reduce the long-term HIV prevalence.

METHODS

To study the introduction of PrEP, we develop a pair-formation model that includes steady (long-term) partnerships and casual (one-off/occasional) sex partners. We categorise individuals as sexually high-active or low-active, with different sex partner mixing patterns, different HIV-diagnosis rates, and allowing for high-actives to use PrEP. We incorporate two stages of HIV infectiousness: the early acute (primary) stage and the subsequent chronic

For peer review only

(asymptomatic) stage.[16] The model is then calibrated to detailed sexual behaviour data and the observed HIV prevalence. We earlier developed a model to disentangle the roles of casual and steady partnerships on HIV transmission, which we further elaborate in this work.[15] We begin by describing the model for sexual contacts and continue with how transmission of HIV-infection is modelled. Then we apply sexual behaviour data gathered among MSM. The full description of the model can be found in the Supplementary material S1.

Dynamic pair-formation model

Consider a sexually active same-sex population where new individuals enter the sexually active population without having a steady sex partner. Individuals can have at most one steady partner at a time, which can end by separation or death of either partner. Individuals can also have casual sex partners during steady partnerships as well as during periods without steady partnerships; the rate at which this occurs depends on the steady partnership status of the individuals under consideration. Based on data, we allow singles to have a higher rate of casual sex than individuals in a steady partnership. Not only partnership status affects the rate of finding new casual sex partners, we additionally allow for individuals to be either high-active or low-active regarding the frequency of having casual sex partners.

Letting the rate of finding a new casual sex partner depend on the partnership status and the activity-degree of both potential members in the sexual act, yields 16 different casual sex partnership combinations. We let α_{ij}^{rq} denote the rate at which an individual with activity-degree r and i steady partners try to find a casual sex partner with activity-degree q and j steady partners, where $r,q \in \{h = \text{high},\ l = \text{low}\}$ and $i,j \in \{0,1\}$. These rates play an

For peer review only - http://bmjopen.bmj.com/site/about/guidelines.xhtml

important role in our modelling and are described in detail in Supplementary material S1 and Supplementary material S2.

Casual sex and mixing patterns

Creating the groups high-active and low-active makes it necessary to formulate mixing between the groups. Three activity-degree mixing assumptions are considered: proportionate mixing, complete assortativity, and mixing fitted to responses of a proxy question. Common to all three models is that high-actives have casual contacts at a fixed rate being larger than that of low-actives. Proportionate mixing implies that an individual chooses a casual partner at random among the potential casual sex attempts in the population, i.e. the probability of having a high-active casual partner is the same for low-active and high-active individuals. Complete assortativity means that high-actives only have high-active casual partners and low-actives only have low-active casual partners.

We estimate the assortativity according to the testing-clinic participants answer to one question on partners' sexual activity, as described in Supplementary material S3. This question is referred to as a proxy question for partners activity-degree. The proxy for partners activity-degree and knowing whether an individual is high-active or low-active, specifies the amount of assortativity in the data. The estimated assortativity can take values between 0 and 1, where 0 corresponds to proportionate mixing and 1 to complete assortativity.

Since we do not know whether or not participants' casual sex partners are in steady partnerships, the mixing between singles and individuals in a partnership is assumed to be random (proportionate mixing).

For peer review only

Model of infection

To model the spread of infection we use a compartmental model, much like the SIR model (Susceptible → Infectious → Recovered),[17] but with two stages of infectiousness: acute followed by chronic. The probability of transmission of HIV depends on the stage of infection when no antiretroviral treatment is used.[18] In the early acute stage the probability of transmission is much higher due to higher viral load than in the chronic stage. The probability of transmission in one unprotected sex act (in our case anal intercourse) is denoted p_A when in the acute stage and p_C when in the chronic stage. The model allows for different levels of condom use with steady and casual sex partners, and it is incorporated by reducing the transmission probability accordingly. The time until diagnosis and the beginning of ART depends on the degree of sexual activity. Further, individuals on ART are assumed to be virally suppressed and thereby to no longer transmit infection. The compartmental model therefore divides the population into susceptible, infectious in the acute stage, infectious in the chronic stage, and being on ART-treatment.

The aim with defining this model is to introduce the possibility for high-actives to take PrEP, which, when taken correctly, decreases the probability of getting infected with HIV by approximately 86%.[9,10] Moreover, individuals accepting PrEP need to test themselves every third month. The model is illustrated in Supplementary material S1.

For peer review only

Data and calibration

The data was gathered at a gay-friendly HIV/STI-testing clinic (Venhälsan) in Stockholm, Sweden, during 2015.[19] MSM visiting testing-clinics might be more sexually active than other MSM, e.g. sexually inactive or MSM with one sex partner might not visit testing-clinics as often. 403 MSM participants answered a structured timeline follow-back questionnaire and reported their total number of sex partners during the last 12 months. Detailed sexual behaviour data was collected on participants' last ten sex partners during the last 12 months, including: type of sex partner (casual or steady); frequency of sex acts; condom use with each partner; the duration of each sexual relationship; and the answer to the proxy question on partners' sexual activity-degree. All 403 participants were included in the study by Hansson et al.[15] However, inclusion in this study requires that participants have reported their total number of sex partners during a year to determine their activity-degree. Moreover, for a partner to be included, the proxy question regarding the partner's sexual activity-degree must be answered. Of the 403 participants, four participants reported having zero sex partners the previous 12 months and 28 participants did not answer that particular question. Of the remaining 371 participants, detailed information on 1991 different sex partners (510 steady and 1481 casual) were given. We have an answer to the proxy question for 1424 of the 1481 casual sex partners. When removing the 57 casual sex partners with no answer to the proxy question, 368 participants (and 1903 partners) remain and was included in the analysis presented here.

The participants demonstrate a considerable difference in yearly number of sexual partners, with a range from 1 to 250 sexual partners. We choose the mean (15) as our cut-off for

For peer review only

defining high-active individuals, resulting in that 33.7% are high-actives. The mean number of sex partners for high-actives is 33.2 (sd=32) and for low-actives it is 6.0 (sd=3.2).

Partnership and epidemic parameters

The parameters and their values used in the analysis are given in Table 1. Some parameters are estimated from the testing-clinic data (part 1 - 4 in Table 1), some parameters are varied (part 2 in Table 1), and some are taken from the available literature (part 5 in Table 1). In the analysis, the standard errors of all estimates from the testing-clinic data can be included to obtain a 95% credibility interval (using Monte Carlo simulation) of the prevalence estimated from the model (see Supplementary material S3).

The mean times to ART, denoted γ_h for a high-active and γ_l for a low-active, are calibrated to fit the observed prevalence. From the data we estimate that $\gamma_h = 2.35\gamma_l$ (Supplementary material S3), this relationship will be kept throughout the analysis, such that only one of the parameters need to vary.

Estimated rates of meeting a new casual sex partner

Table 1 shows the estimated rates of meeting a new casual sex partner. In the third part of Table 1 we have not utilised the proxy question on partners activity-degree, these estimates are enough when assuming proportionate mixing or complete assortativity. The proxy question is used to get the estimates in the fourth part of Table 1. From this table and a given choice of mixing pattern, one can estimate the rates of looking for a new casual sex partner α_{ij}^{rq}. The final values of α_{ij}^{rq} can be found in Supplementary material S3.

For peer review only

Table 1: *Estimates of partnership and epidemic parameters. Abbreviations used: AI - anal intercourse, URAI - unprotected receptive anal intercourse, UIAI - unprotected insertive anal intercourse.*

**1. Partnership parameters**			
**Parameter**	**Value**	**Definition**	**Source**
$1/(\sigma + 2\mu)$	271.5 days	Mean duration steady partnership	MSM data
$1/\rho P_0$	152.8 days	Mean time being single	MSM data
$1/\lambda$	12.3 days	Mean time between AI within steady partnership	MSM data
P_0	0.360	Fraction without a steady partner	MSM data
P_1	0.640	Fraction with a steady partner	MSM data
π_h	0.337	Fraction high-actives	MSM data
π_l	0.663	Fraction low-actives	MSM data
**2. Parameters for condom use, PrEP, and time to treatment**			**Source**
q_s	54.1%	Mean condom use steady partner	MSM data
q_c	62.9%	Mean condom use casual partner	MSM data
ξ		Rate for a high-active to start taking PrEP (calibrated to achieve different % PrEP coverage)	Varied
		**Mean time from infection to successful antiretroviral treatment for a**	
$1/\gamma_P$	0.25 years	high-active on PrEP	[20]
$1/\gamma_h$	1.5 – 3 years	high-active not on PrEP	Varied
$1/\gamma_l$	3.5 – 7 years	low-active	$1/\gamma_l = 2.35\ /\gamma_h$
**3. Casual sex partner parameters from data not using proxy**			**Source**
		**Mean time until new casual sex partner for a**	
$1/\alpha_0^h$:	10.7 days	high-active when single	MSM data
$1/\alpha_1^h$:	12.5 days	high-active when in partnership	MSM data
$1/\alpha_0^l$.	66.5 days	low-active when single	MSM data
$1/\alpha_1^h$:	97.9 days	low-active when in partnership	MSM data
**4. Casual sex partner parameters from data using proxy**			**Source**
		**Mean time until new casual partner for a high-active with a**	
$1/\alpha_0^{hh}$.	14.1 days	high-active when single	MSM data
$1/\alpha_0^{hl}$.	43.7 days	low-active when single	MSM data
$1/\alpha_1^{hh}$.	15.4 days	high-active when in partnership	MSM data
$1/\alpha_1^{hl}$.	66.4 days	low-active when in partnership	MSM data
		**Mean time until new casual partner for a low-active with a**	
$1/\alpha_0^{lh}$.	109.3 days	high-active when single	MSM data
$1/\alpha_0^{ll}$.	169.6 days	low-active when single	MSM data
$1/\alpha_1^{lh}$.	136.1 days	high-active when in partnership	MSM data
$1/\alpha_1^{ll}$.	348.9 days	low-active when in partnership	MSM data
**5. Parameters from the literature**			
**Parameter**	**Value**	**Definition**	**Source**
	70%	Condom efficiency	[21]
$1/\delta_a$	0.24 years	Mean time in acute infection stage	[18]
$1/\mu$	60 years	Sexually active life-span	[22,23]
		**Per-act transmission probability**	
	0.1835	Acute stage URAI	[16]
	0.0138	Chronic stage URAI	[16]
	1.48%	Overall URAI	[24]
	0.62%	Overall UIAI	[24]
	2.39	Of URAI in comparison to UIAI	[24]
p_A	0.1301	Acute stage combined URAI-UIAI	[16,24]
p_C	0.0098	Chronic stage combined URAI-UIAI	[16,24]

For peer review only

Patient and public involvement

There was no patient and/or public involvement in the planning of this study.

ANALYSIS AND RESULTS

The assortativity regarding activity-degree is measured to 0.14, meaning that the studied population choose casual partners with a moderate assortativity. The HIV prevalence among the 368 participants is 5%, which is in line with national levels among MSM,[2] and this prevalence will be used as a baseline when studying the effect of PrEP. Specifically, the baseline model is the model where no one uses PrEP ($\xi = 0$) and that is calibrated to achieve a 5% equilibrium prevalence. The only parameter not being able to be estimated from the HIV/STI-testing clinic data, or that can be taken from the literature, is the mean time to successful ART-treatment. Hence, to calibrate the model to the observed 5% prevalence, we find the mean time to treatment that corresponds to this prevalence. To study the effect of PrEP, we use the same parameter set-ups as for the baseline model but additionally allow for sexually high-actives to use PrEP ($\xi > 0$), and then find the new equilibrium prevalence. We use ‘long-term prevalence’ and ‘equilibrium prevalence’ interchangeably. The set of equations used to find the equilibrium prevalence can be found in Supplementary material S4. We begin by presenting the results of the model where PrEP has not yet been introduced, then we move to the model where high-actives are offered PrEP.

Prior to the introduction of PrEP

For peer review only

In dividing the population into two activity-degrees and using fitted assortativity, we find that the prevalence of 5% (95% CRI 2.3-7.6%) is obtained when the mean time to ART is $\gamma_h^{-1} = 1.77$ years for high-actives, (4.15 years for low-actives). In doing this calibration to the observed 5% prevalence, the estimated percent of individuals with positive HIV serostatus that are on ART-treatment is 95.8%.

Disregarding the proxy question, we would not know how the population mix regarding activity-degree. We could then use the other two mixing patterns. Assuming proportionate mixing, the prevalence of 5% (95% CRI 2.1-7.8%) is obtained when $\gamma_h^{-1} = 1.79$ years. This set-up yields that the estimated percent of individuals with HIV that are on ART-treatment is 95.7%. For complete assortativity, the prevalence of 5% (95% CRI 2.7-7.0%) is obtained when $\gamma_h^{-1} = 1.63$ years, and then the estimated percent of individuals with HIV that are on ART-treatment is 96.4%. Figure 1a) depicts the prevalence for varied values of the mean time to ART-treatment and Figure 1b) shows the 95% credibility intervals.

[INSERT FIGURE 1 HERE]

Using the fitted assortativity, a prevalence of 5% was found when the mean time to ART was 1.77 years for high-actives, while the same time to ART for the proportionate mixing assumption yields a prevalence of 4.6% (95% CRI 1.7-7.3%), and complete assortativity yields a prevalence of 6.9% (95% CRI 4.6-8.8%). This shows that higher assortativity regarding activity-degree leads to higher prevalence and easier allows for HIV being endemic. With increased assortativity, the allocation of the infected individuals becomes different, as seen in Table 2, with more high-actives being infected. Interesting to note, from Figure 1a, is that the difference between the mixing assumption decreases with an increased time to ART.

For peer review only

Additionally, from Table 2 we note that approximately 35% of HIV transmissions occur within a steady partnership.

Table 2: For the three mixing assumptions, we show the estimated mean time to ART corresponding to a prevalence of 5%. For this prevalence and for each of the three mixing assumptions, we also show the route of transmissions and HIV prevalence for the two respective activity-degree groups. The shown values for the time to ART-treatment are for high-active individuals, the time to ART-treatment for low-actives is 2.35 times larger. For the allocation of the 5% infected we show the percentage of high-actives and low-actives, respectively, that are HIV-positive.

	**Overall HIV prevalence of 5%**		
	Prop. mixing	Fitted Assort.	Compl. Assort.
**Time to ART (years)**	1.79	1.77	1.63
**Route of transmission**			
Steady partner	35%	35%	32%
Casual sex when in partnership	38%	39%	41%
Casual sex when single	26%	26%	27%
**HIV prevalence in the group**			
High-actives	9.05%	9.23%	10.79%
Low-actives	2.94%	2.85%	2.06%

Effect of introducing PrEP

We now present the effect of introducing PrEP. This is done by starting at a prevalence of 5% and then increasing the PrEP coverage. That is, we use the parameter values from the model without PrEP that achieved a 5% equilibrium prevalence, but now allow high-actives to take PrEP and find the new equilibrium prevalence. We stress that the results are the long-term effect of certain levels of PrEP coverages, even with no more infections it will naturally take a long time to eliminate HIV completely from the MSM community, i.e. for the HIV prevalence to reach 0%.

For peer review only

If the population is not divided according to activity-degree, the PrEP coverage would need to be 5.2% of the total population to reduce the long-term prevalence from the observed 5% to close to 0% (Supplementary material S5). Dividing the population according to activity-degree and only targeting high-actives for PrEP, Figure 2a shows the combined effect of PrEP and an increased testing rate: reaching a coverage of 1% of the population (3.0% of high-actives) will reduce the long-term prevalence from 5% to 3.6%; reaching a coverage of 3.5% of the population (10.4% of high-actives) will reduce the long-term prevalence to 0%.

[INSERT FIGURE 2 HERE]

Being able to target risk groups for PrEP makes a big difference: targeting low-actives instead would result in a needed PrEP coverage of 34.4% of the population to eventually eliminate HIV from the community (Supplementary material S5).

To ascertain the respective effects of PrEP, the decreased susceptibility by 86% and the more frequent HIV-testing rate (every third month), we do two additional analyses. If being on PrEP is not combined with an increased testing rate, but only a reduced susceptibility, reaching a coverage of 3.5% of the population will reduce the prevalence to 0.5% (Figure 2b). If being on PrEP does not give any reduced susceptibility, but only an increased testing rate, reaching a coverage of 3.5% of the population will reduce the prevalence to 1.9%.

For results concerning the more short-term effect of PrEP, see Supplementary material S5. We can for example note that using the lowest PrEP-initiation rate that corresponds to HIV elimination (the 3.5% PrEP coverage) would after 50 years have reduced the prevalence from 5% to 4% and the percentage infectious and undiagnosed HIV cases by half (from 0.21% to 0.09%). Using a higher PrEP-initiation rate would speed up the decrease in new HIV cases, for

For peer review only

example by reaching a PrEP coverage of 7% after 50 years would result in that the percentage of infectious undiagnosed HIV cases would be as low as 0.01%.

DISCUSSION

Our results suggest that a PrEP coverage of at least 3.5% of the MSM population, when sexually high-actives are targeted, is needed to eventually eliminate HIV from the MSM population in Sweden. This can be compared to a 34.4% PrEP coverage needed if only low-active MSM were targeted for PrEP. These results emphasise the need for risk stratification among MSM, to ensure that those in need of PrEP receive the intervention. To reach high-risk MSM, out-reach programmes and peer education programmes have been found to be effective, scale up of these may increase the effect of PrEP implementation on the HIV epidemic among MSM in Sweden.[25]

We find that the greatest effect of the combined PrEP intervention follows from the decreased susceptibility to HIV, not the increased HIV-testing rate. This result would be hidden in a model not taking the different stages of infection into account (Supplementary material S5). Hence, to make a correct assessment of a PrEP programme's effect, the complexities of HIV transmission, the different stages of infection, need to be accounted for.

The benefit of targeting high-risk individuals for PrEP has been found by other studies.[8,26–29] Our analyses adds to these findings by including additional parameters. Punyacharoensin et al.[8] investigate the effect of different HIV interventions, including PrEP, among MSM in

For peer review only

the UK. They define low-actives as MSM with one or fewer new sexual partners a year, while our definition of high-active MSM (at least 15 partners a year) is to address a group with very high HIV risk. Secondly, they address mixing through a different method using an odds ratio among male heterosexuals, while we use data from the MSM population under study. Rozhnova et al.[29] use four risk-groups, however, they do not estimate mixing between the groups but assume intermediate mixing.

Our model has four strengths worth mentioning. First, our model design is strengthened by that it is calibrated to fit detailed data of MSM who visited an STI/HIV-testing clinic in Sweden. For example, when the model is calibrated to data and the observed 5% HIV prevalence, the estimated percentage of individuals with HIV that are on ART-treatment, 95.8%, are very close to the observed value of 95.1%.[1] In addition, the very same clinic where the data was gathered is the largest implementer of PrEP in Sweden, prescribing PrEP to 315 MSM since October 2018. Secondly, we can measure the assortativity with respect to activity-degree of the study participants. The mixing between high-actives and low-actives is estimated to be more assortative than proportionate mixing (0.14 vs 0), and by using the estimated assortativity we get more reliable results with narrower 95% CRIs than for proportionate mixing (Supplementary material S5). For realistic mean times to ART, the mixing assumption has an impact on the estimated prevalence, making it an important factor to include. Thirdly, an important model choice is to include steady partnerships, not only casual contacts, since HIV transmission occurs to a large extent within a steady partnership (Table 2). Finally, the result concerning PrEP coverage is robust to variations in the parameter set-ups (Supplementary material S5).

For peer review only

Our model includes limitations. First, the proxy question used to fit the assortativity can only define two activity-degree groups and not more. The real-life scenario is probably more heterogeneous than accounted for in our model; even with a high PrEP coverage, the prevalence would likely stay above 0% due to some sub-groups of MSM taking larger HIV risks than the high-actives within our model. Additionally, some individuals are probably even more low-active (such as MSM in monogamous steady partnerships) than we allow for. Secondly, many possible changes in sexual behaviour are not included. Our model assumes no change in sexual behaviour when being on ART and we do not assume any increased HIV-testing rate for a sex partner to someone living with HIV. Individuals on PrEP are assumed to stay on PrEP, except if they get diagnosed with HIV and are put on ART-treatment. Moreover, individuals are assumed to belong to one of the sexual activity-groups during their whole life. For the studied MSM population we do not have data on how individuals change between the two activity-groups, due to that the timeline data was restricted to 12 months. To study the potential effect of behavioural changes of activity-degree we allowed low-active MSM to become high-active and for high-active MSM to become low-active. We looked at several different magnitudes of switching between low-active and high-active and these analyses can be found in Supplementary material S5. In this extension of the model we also allowed for PrEP-users to stop taking PrEP. We find the same tendency as Rozhnova et al.[30]—that a slightly less PrEP coverage is needed to reach a long-term HIV prevalence of 0% when allowing individuals to change activity degree group. When individuals are fixed to one group the PrEP coverage needed to eliminate HIV was 3.5% while when allowing for switching the PrEP coverage needed varies between 2.5% - 3%. Thirdly, the data is collected among a convenience sample of MSM visiting an HIV/STI-testing clinic, thereby it is not representative of all MSM in Sweden. Fourthly, the model does not consider imperfect PrEP adherence which

For peer review only

could overestimate PrEP's potential effect in reducing the HIV prevalence. However, in Supplementary material S5 we consider different values of PrEP effectiveness. Finally, our model does not incorporate concurrent steady partnerships. This is a common assumption for compartmental models,[8,15,31] and inclusion would possibly strengthen the model. However, our model does consider casual sex partners concurrent to steady partners.

In future work, risk compensation could be studied more thoroughly, e.g. changed behaviour of individuals on PrEP. Other risk behaviours for HIV than sexual activity-degree could be considered to define the risk-group offered PrEP, such as taking part in group sex, consistent drug use, and transactional sex. Another possible extension is to stratify our model by age, letting also activity-degree vary between the age groups to capture that certain age groups could be more sexually active.

We conclude by stating the result emerged from the heterogeneous activity-degrees: heterogeneity in sexual activity does increase the prevalence, however, it also makes targeted interventions much more effective.

Acknowledgements The authors thank the study participants for their contribution and acknowledge the work of staff at Venhälsan STI/HIV clinic in Stockholm, Sweden.

Contributions DH, SS, KYL, and TB conceived the study; SS designed and managed the gathering of data; DH, TB and KYL defined the model. DH, SS and TB drafted the manuscript. All authors approved the manuscript before submission for publication.

Funding The data collection was financially supported by the Swedish State Grant for HIV/STI prevention (HSN 1309-1016) and an unrestricted grant from Gilead Sciences Nordic

For peer review only

Fellowship Programme 2014. The funders had no role in study design, data collection, analysis or write up of results. TB and KYL are supported by The Swedish Research Council, grant no. 2015-05015.

Competing interests None to declare.

Patient consent for publication Written informed consent was provided by all participants.

Ethics approval Ethical approval for the study data collection was obtained from the Regional Ethical Committee in Stockholm, Sweden (Dnr. 2014/1729-31/5).

Data availability statement The full data set will be shared upon reasonable request to SS in order to protect participant confidentiality. This is motivated by that the data set contains sensitive information on sexual behaviour on a rather small sample of a stigmatised population (MSM), and sociodemographic data might theoretically make the data traceable.

REFERENCES

1 Gisslén M, Svedhem V, Lindborg L, *et al.* Sweden, the first country to achieve the Joint United Nations Programme on HIV/AIDS (UNAIDS)/World Health Organization (WHO) 90-90-90 continuum of HIV care targets. *HIV Med* 2017;**18**:305–7. doi:10.1111/hiv.12431

2 The Public Health Agency of Sweden. MSM2013, A study about sex, HIV, and health among men who have sex with men in Sweden. https://www.folkhalsomyndigheten.se/contentassets/4133e13e29204433a459d5ebbf0fcebe/msm2013-studie-sex-hiv-halsa-15139.pdf

3 Ministry of Health and Social A airs GO ces of S. Nationell strategi mot hiv/aids och vissa andra smittsamma sjukdomar.

For peer review only

2017.https://www.regeringen.se/informationsmaterial/2017/12/nationell-strategi-mot-hivaids-och-andra-smittsamma-sjukdomar/ (accessed 6 May 2018).

4 Cohen MS, Chen YQ, McCauley M, *et al.* Prevention of HIV-1 Infection with Early Antiretroviral Therapy. *N Engl J Med* 2011;**365**:493–505. doi:10.1056/NEJMoa1105243

5 HIV TL. U=U taking off in 2017. *Lancet HIV* 2017;**4**:e475. doi:10.1016/S2352-3018(17)30183-2

6 Cremin I, Alsallaq R, Dybul M, *et al.* The new role of antiretrovirals in combination HIV prevention: a mathematical modelling analysis. *AIDS* 2013;**27**.https://journals.lww.com/aidsonline/Fulltext/2013/01280/The_new_role_of_antiretrovirals_in_combination_HIV.15.aspx

7 Beyrer C, Sullivan P, Sanchez J, *et al.* The increase in global HIV epidemics in MSM. *AIDS* 2013;**27**.https://journals.lww.com/aidsonline/Fulltext/2013/11130/The_increase_in_global_HIV_epidemics_in_MSM.1.aspx

8 Punyacharoensin N, Edmunds WJ, De Angelis D, *et al.* Effect of pre-exposure prophylaxis and combination HIV prevention for men who have sex with men in the UK: a mathematical modelling study. *Lancet HIV* 2016;**3**:e94–104. doi:10.1016/S2352-3018(15)00056-9

9 Molina J-M, Capitant C, Spire B, *et al.* On-Demand Preexposure Prophylaxis in Men at High Risk for HIV-1 Infection. *N Engl J Med* 2015;**373**:2237–46. doi:10.1056/NEJMoa1506273

For peer review only

10 McCormack S, Dunn DT, Desai M, *et al.* Pre-exposure prophylaxis to prevent the acquisition of HIV-1 infection (PROUD): effectiveness results from the pilot phase of a pragmatic open-label randomised trial. *Lancet (London, England)* 2016;**387**:53–60. doi:10.1016/S0140-6736(15)00056-2

11 Sidebottom D, Ekström AM, Strömdahl S. A systematic review of adherence to oral pre-exposure prophylaxis for HIV - how can we improve uptake and adherence? *BMC Infect Dis* 2018;**18**:581. doi:10.1186/s12879-018-3463-4

12 World Health Organization. *Consolidated guidelines on the use of antiretroviral drugs for treating and preventing HIV infection. Recommendations for a public health approach - Second edition*. 2016. https://www.who.int/hiv/pub/arv/arv-2016/en/

13 The Public Health Agency of Sweden. Preexpositionsprofylax för att minska risken för infektion med hiv – en kunskapsöversikt. 2017.https://www.folkhalsomyndigheten.se/publicerat-material/publikationsarkiv/p/preexpositionsprofylax-for-att-minska-risken-for-infektion-med-hiv-en-kunskapsoversikt/ (accessed 13 Sep 2018).

14 NT-rådet. Emtricitabin tenofovirdisoproxil som preexpositionsprofylax (PrEP) för att minska risken för hiv-infektion. https://janusinfo.se/download/18.11b119de1639e38ca5fb2e4a/1535626547383/Emtricitabin-tenofovirdisoproxil-(PrEP)-180611.pdf

15 Hansson D, Leung KY, Britton T, *et al.* A dynamic network model to disentangle the roles of steady and casual partners for HIV transmission among MSM. *Epidemics* Published Online First: 2019. doi:https://doi.org/10.1016/j.epidem.2019.02.001

For peer review only

16 Leynaert B, M Downs A, de Vincenzi I. *Heterosexual transmission of human immunodeficiency virus: variability of infectivity throughout the course of infection. European Study Group on Heterosexual Transmission of HIV*. 1998. doi:10.1093/oxfordjournals.aje.a009564

17 Ogilvy KW, G. MA, Thomas WG. A contribution to the mathematical theory of epidemics. *Proc R Soc London Ser A, Contain Pap a Math Phys Character* 1927;**115**:700–21. doi:10.1098/rspa.1927.0118

18 Hollingsworth TD, Anderson RM, Fraser C. HIV-1 Transmission, by Stage of Infection. *J Infect Dis* 2008;**198**:687–93.http://dx.doi.org/10.1086/590501

19 Sidebottom D, Hansson D, Liljeros F, *et al.* Comprehensive measurement of HIV/STI risk behaviour among MSM using a tablet-based timeline follow-back tool. *Submitted*

20 (RAV) TSRG for AT. HIV preexpositionsprofylax (PrEP) – Rekommendation om praktisk handläggning 2017. https://www.sls.se/globalassets/rav/rekommendationer/rav_hiv_prep_2017_v170824.pdf

21 Smith DK, Herbst JH, Zhang X, *et al.* Condom Effectiveness for HIV Prevention by Consistency of Use Among Men Who Have Sex With Men in the United States. *JAIDS J Acquir Immune Defic Syndr* 2015;**68**.https://journals.lww.com/jaids/Fulltext/2015/03010/Condom_Effectiveness_for_HIV_Prevention_by.14.aspx

22 The Public Health Agency of Sweden. Sexualitet och hälsa bland unga i Sverige – UngKAB15 – en studie om kunskap, attityder och beteende bland unga 16–29 år.

For peer review only

2017. https://www.folkhalsomyndigheten.se/publicerat-material/publikationsarkiv/s/sexualitet-och-halsa-bland-unga-i-sverige-ungkab15-en-studie-om-kunskap-attityder-och-beteende-bland-unga-1629-ar/

23 World Bank. Life expectancy at birth 2015. https://data.worldbank.org/indicator/SP.DYN.LE00.MA.IN?locations=SE (accessed 12 Jul 2017).

24 Jin F, Jansson J, Law M, *et al.* Per-contact probability of HIV transmission in homosexual men in Sydney in the era of HAART. *AIDS* 2010;**24**.https://journals.lww.com/aidsonline/Fulltext/2010/03270/Per_contact_probability_of_HIV_transmission_in.14.aspx

25 Strömdahl S, Hoijer J, Eriksen J. Uptake of peer-led venue-based HIV testing sites in Sweden aimed at men who have sex with men (MSM) and trans persons: a cross-sectional survey. *Sex Transm Infect* 2019;:sextrans-2019-054007. doi:10.1136/sextrans-2019-054007

26 Pretorius C, Stover J, Bollinger L, *et al.* Evaluating the cost-effectiveness of pre-exposure prophylaxis (PrEP) and its impact on HIV-1 transmission in South Africa. *PLoS One* 2010;**5**:e13646–e13646. doi:10.1371/journal.pone.0013646

27 Abbas UL, Anderson RM, Mellors JW. Potential Impact of Antiretroviral Chemoprophylaxis on HIV-1 Transmission in Resource-Limited Settings. *PLoS One* 2007;**2**:e875.https://doi.org/10.1371/journal.pone.0000875

28 Vissers DCJ, Voeten HACM, Nagelkerke NJD, *et al.* The Impact of Pre-Exposure Prophylaxis (PrEP) on HIV Epidemics in Africa and India: A Simulation Study. *PLoS One*

For peer review only

2008;**3**:e2077.https://doi.org/10.1371/journal.pone.0002077

29 Rozhnova G, Heijne J, Bezemer D, *et al.* Elimination prospects of the Dutch HIV epidemic among men who have sex with men in the era of preexposure prophylaxis. *AIDS* 2018;**32**:2615–23. doi:10.1097/QAD.0000000000002050

30 Rozhnova G, Heijne JCM, Basten M, *et al.* Impact of sexual trajectories of men who have sex with men on the reduction in HIV transmission by pre-exposure prophylaxis. *Epidemics* 2019;**28**:100337. doi:https://doi.org/10.1016/j.epidem.2019.03.003

31 A. van WD, Chantal den D, E. KME, *et al.* Double trouble: modelling the impact of low risk perception and high-risk sexual behaviour on chlamydia transmission. *J R Soc Interface* 2018;**15**:20170847. doi:10.1098/rsif.2017.0847

For peer review only

FIGURE TITLES

Figure 1: (a) Estimated prevalence of HIV (*y*-axis) for the three mixing assumptions and different mean time to ART-treatment (*x*-axis). The presented time to ART-treatment is for high-active individuals, for low-actives it is a factor 2.35 higher. (b) The same estimated prevalence as in (a), but now showing the prevalence separately and including the 95% credibility interval for the three mixing assumptions. In one simulation, each partnership parameter (estimated from data) was drawn from its distribution. With that set-up of drawn parameters, we calculated the prevalence. This was repeated 1000 times to obtain the credibility interval.

Figure 2: The effect of introducing PrEP to sexually high-actives. In (a) the *x*-axis shows different PrEP coverage levels and the *y*-axis the corresponding HIV prevalence. The three lines show: the HIV prevalence in the total population (black solid line), the HIV prevalence among high-actives (lighter short-dashed line), and the HIV prevalence among low-actives (darker long-dashed line). (b) depicts the effect of PrEP by looking at: (i) solely the reduction of susceptibility and no increased testing rate; and (ii) solely the increased testing rate and no reduced susceptibility.

For peer review only

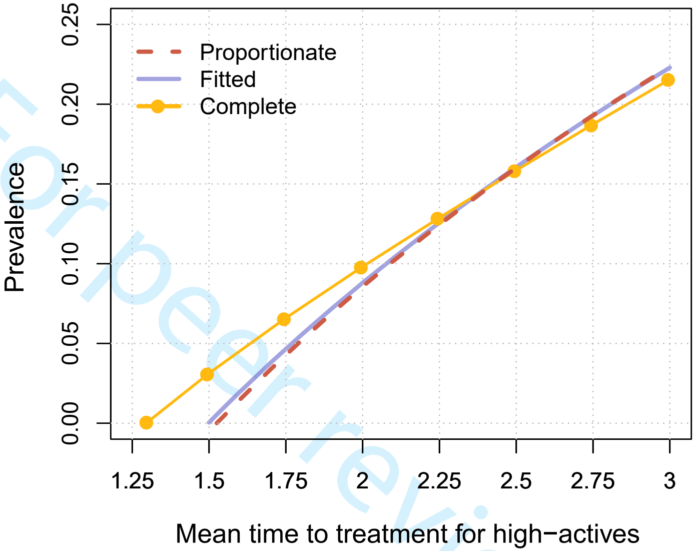

(a)

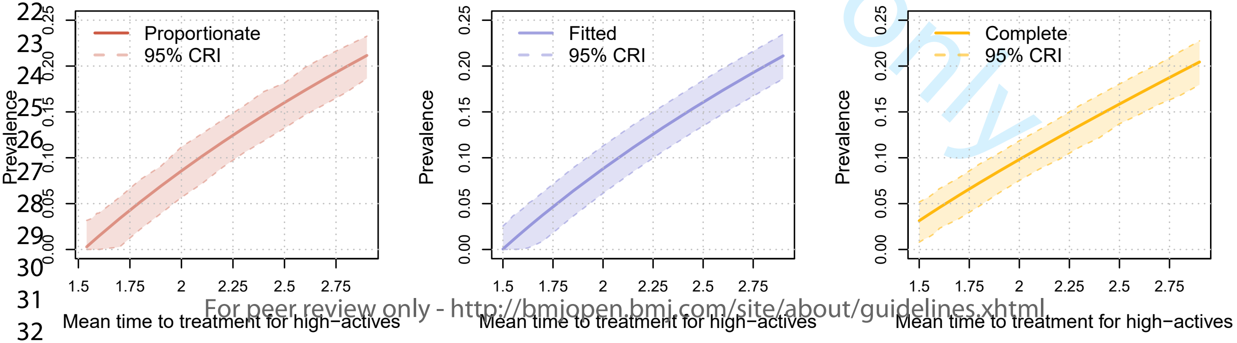

(b)

For peer review only

For peer review only - http://bmjopen.bmj.com/site/about/guidelines.xhtml

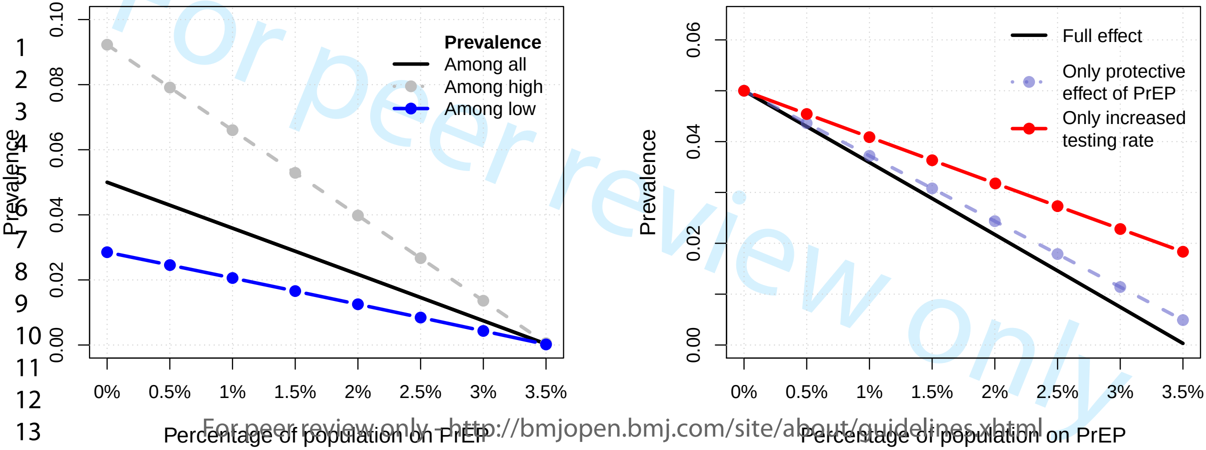

(a) (b)

For peer review only - http://bmjopen.bmj.com/site/about/guidelines.xhtml

Online Supplementary Material: Introducing pre-exposure prophylaxis to prevent HIV acquisition among men who have sex with men in Sweden: insights from a mathematical pair-formation model

Disa Hansson, Susanne Strömdahl, KaYin Leung and Tom Britton

For peer review only

Contents

S1 Formulation of the model **2**
S1.1 Pair-formation model . . . 2
S1.2 Model of infection . . . 2

S2 Rate of new casual sex partners **5**
S2.1 Proportionate Mixing with respect to activity degree . . . 7
S2.2 Complete assortativity . . . 7
S2.3 Mixing determined by the proxy question . . . 8

S3 Data and parameter estimates **8**
S3.1 Data description . . . 8
S3.2 Scaling of the rate of finding a new casual sex partner . . . 8
S3.3 Time since last HIV-test . . . 9
S3.4 Distribution of parameters . . . 9
S3.5 Proxy of partners' activity degree . . . 12
S3.6 Final estimates of the rates of acquiring new casual sex partners . . . 13

S4 Finding the endemic prevalence, a deterministic approximation **15**
S4.1 Single states . . . 17
S4.2 Partnership states . . . 18
S4.2.1 Susceptible not on PrEP . . . 18
S4.2.2 Acute infectious individuals not on PrEP . . . 20
S4.2.3 Chronic infectious not on PrEP . . . 21
S4.2.4 Treated individual . . . 22
S4.2.5 Individuals on PrEP . . . 22

S5 Additional results **24**
S5.1 Not distinguishing individuals according to activity degree . . . 24
S5.2 Not including the different infectious stages acute and chronic . . . 24
S5.3 Range of 95% CRI . . . 25
S5.4 Effect of different PrEP effectiveness in reducing susceptibility . . . 25
S5.5 Effect of giving PrEP to low-actives instead of high-actives . . . 25
S5.6 PrEP coverage for alternative transmission probabilities . . . 28
S5.7 Short-term effect of different PrEP strategies . . . 29
S5.8 Allowing individuals to change activity-group . . . 31

For peer review only - http://bmjopen.bmj.com/site/about/guidelines.xhtml

S1 Formulation of the model

S1.1 Pair-formation model

We consider a sexually active same-sex population, where new individuals enter the sexually active population according to an exponential distribution with rate μn and each individual leaves the sexually active population at rate μ. The size of the sexually active population will therefore fluctuate around the value n, which is assumed to be large. Individuals enter the sexually active population without a steady partner. The rate at which an individual who is single enters into a partnership is ρP_0, where P_0 is the fraction of single individuals in the population. This means that the higher the fraction of single individuals, the higher the pair-formation rate. Individuals can have at most one steady partner at a time and the separation rate for each partnership is denoted σ. Therefore, a partnership lasts for an exponential time with mean duration $1/(\sigma + 2\mu)$.

The partnership network is assumed to be stable, i.e. the proportion of singles remains at P_0 for all t. We can then express P_0 (and the proportion $P_1 = 1 - P_0$ of individuals with a partner) in terms of model parameters [S1]:

$$P_0 = \frac{\sqrt{(\sigma + 2\mu)(4\rho + \sigma + 2\mu)} - (\sigma + 2\mu)}{2\rho}. \tag{S1.1}$$

The rate of sexual acts within a partnership is denoted λ. Beside steady partners, individuals may have casual sex partners during steady partnerships as well as during single periods; the rate at which this occurs depends on the partnership status of the individual under consideration.

Up to this point, the pair-formation model described is the same as in [S2]. The first extension of the model from [S2] is to allow for individuals to be either low-active or high-active with regards to the number of casual sex partners. In our application, an individual is assumed to be sexually high-active if they have 15 or more sex partners per year. The fractions of sexually high-active and sexually low-active in the population are denoted π_h and π_l, respectively ($\pi_h + \pi_l = 1$).

Let α_{ij}^{rq} be the rate an individual with activity degree $r \in \{l = \text{low}, h = \text{high}\}$ and $i \in \{0, 1\}$ steady partners tries to find a casual sex partner with activity degree $q \in \{l = \text{low}, h = \text{high}\}$ and $j \in \{0, 1\}$ steady partners. For this attempt to succeed the individual must actually meet an individual with activity degree q and j steady partners, and therefore, the rate of actual casual sex is $\alpha_{ij}^{rq} P_j \pi_q$. For example, a single who is low-active has casual sex with another low-active single at rate $\alpha_{00}^{ll} P_0 \pi_l$, and with a high-active individual in a steady partnership at rate $\alpha_{01}^{lh} P_1 \pi_h$.

S1.2 Model of infection

As explained in the main text, to model an infection on the network we use a so-called SIR compartmental model (for a survey on stochastic SIR models see [S3]). Individuals can either be susceptible (S), infectious in the acute stage (A), infectious in the chronic stage (C), or on ART-treatment (T). The second extension of the model in [S2] is to allow for these two infectious stages. Once an individual becomes aware of their infection and starts ART-treatment they are interpreted as immune and can no longer transmit infection. The average duration of the acute infection stage is 2.9 months (= 0.24 years) [S4]. Hence, an individual goes from A to C at rate $\delta_a = 1/0.24$ years^{-1}.

Given an unprotected sexual contact (in our case anal intercourse) between an infectious and susceptible individual, there is a probability of transmission depending on stage of infection: p_A when in the acute stage and p_C when in the chronic stage. Therefore, the transmission rate for an infectious individual in the acute stage in a steady partnership with a susceptible individual is $p_A \lambda$, and the transmission rate in a casual sexual encounter is $p_A \alpha_{ij}^{rq}$. Note that the probabilities p_A and p_C of transmission are for the unprotected case, in reality some of the intercourses are with condom. Condom use may also differ with steady and casual sex partners.

The third extension to [S2] is that the time until diagnosis and the beginning of successful ART-treatment may depend on the degree of sexual activity. A sexually high-active individual is put on ART-treatment at rate γ_h and a sexually low-active at rate γ_l.

For peer review only - http://bmjopen.bmj.com/site/about/guidelines.xhtml

Table S1: **Summary of model parameters.** The partnership formation model parameters are given in the first part of the table and the parameters connected to the epidemic in the second part.

**Partnership parameters**	
n	average population size
μ	rate of leaving the sexually active population
ρ	partnership formation rate
σ	separation rate
λ	rate of sex acts within a steady partnership
π_h	fraction of high-active individuals
π_l	fraction of low-active individuals
ξ	rate for high-actives to start taking PrEP
α_{ij}^{rq}	rate for a r-active individual with i steady partners to try to have casual sex with a q-active with j steady partners
**Epidemic parameters**	
p_A	probability of infection in one unprotected anal intercourse during the acute infectious stage
p_C	probability of infection in one unprotected anal intercourse during the chronic infectious stage
γ_h	ART-treatment rate for high-actives
γ_l	ART-treatment rate for low-actives
γ_P	ART-treatment rate for high-actives on PrEP
δ_a	rate of going from acute infection stage to the chronic stage

The fourth and main extension is to introduce the possibility for a high-active to take PrEP, which dramatically decreases the probability of getting infected with HIV. The rate a high-active initiate PrEP is denoted ξ, and in our model the use of PrEP reduces the per-act probability of infection by 86% [S5]. Note that the rate ξ does not include imperfect PrEP adherence; ξ is the rate for high-actives to initiate PrEP and to achieve its protective effect. A high-active on PrEP is tested and, if HIV-positive, put on ART-treatment at a rate $\gamma_P = 1/0.24$ years^{-1}.

To summarise, the model is captured by 30 parameters (20 free parameters): n, μ, ρ, σ, λ, p_A, p_C, δ_a, ξ; the fraction high-actives π_h and the fraction and low-actives $\pi_l = 1 - \pi_h$; the 16 parameters α_{ij}^{rq} (8 free), where $r, q \in \{l, h\}$ and $i, j \in \{0, 1\}$; and the three γ_P, γ_h and γ_l, where $\gamma_h = 2.349\gamma_l$ as described later in Section S3.3. We provide an overview of the notation in Table S1 and an illustration of the model in Figure S1. Note that, we could instead allow for low-actives, instead of high-actives, to be offered PrEP. This possibility will be briefly explored to be compared to the effect of targeting HIV high-risk individuals for a PrEP intervention programme.

For peer review only - http://bmjopen.bmj.com/site/about/guidelines.xhtml

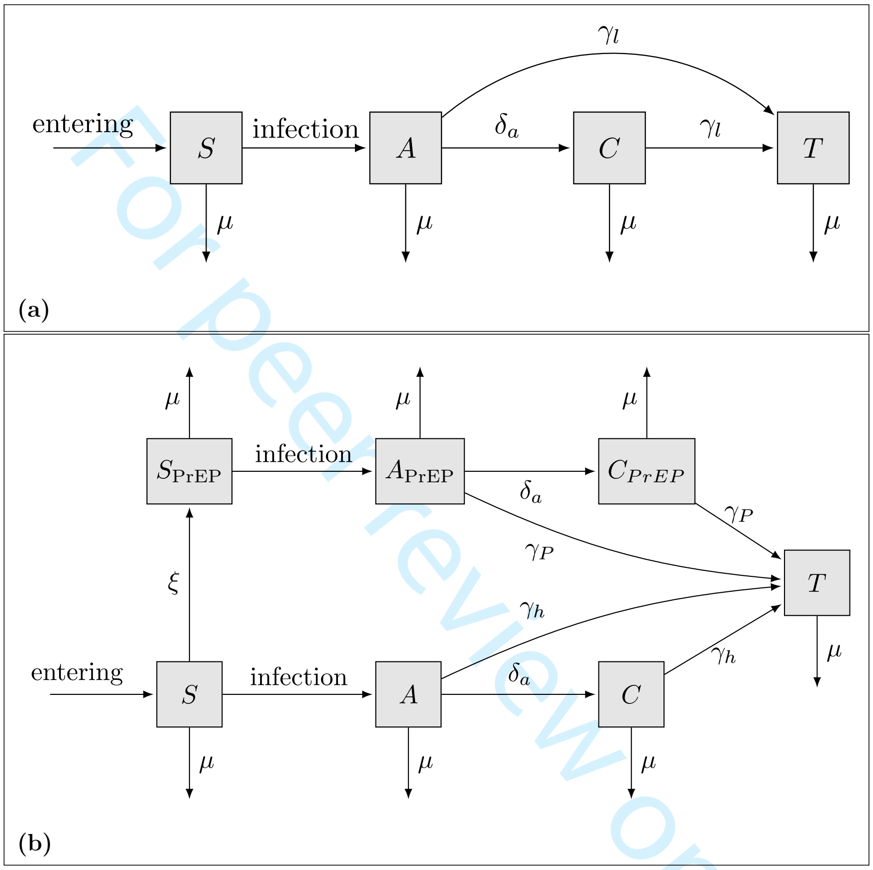

Figure S1: Representation of possible states a low-active (a) and a high-active (b) can be in. Individuals enter the MSM population as singles into the S compartment. High-actives move to S_{PrEP} at a rate ξ whereas low-actives can never start to use PrEP. Susceptible individuals who acquire infection move to the A compartment (acute infection). Individuals in the A compartment can move to the C compartment (chronic infection) at rate δ_a or to the T compartment (ART-treatment). The rate an individual moves to the T compartment is γ_P for a high-active on PrEP, γ_h for a high-active not on PrEP, and γ_l for a low-active. Individuals in the T compartment stay there until they leave the sexually active population. The rate of leaving the sexually active population is denoted μ.

For peer review only - http://bmjopen.bmj.com/site/about/guidelines.xhtml

S2 Rate of new casual sex partners

From our egocentric data on the sexual behaviour, we can determine if a participant is low-active or high-active and if he was single or in a steady partnership while having casual sex. It is therefore possible to obtain an estimate of the rate that an $r-$active individual with i steady partners finds new casual sex partners, let us denote this by $\alpha_{i\cdot}^{r\cdot}$, where $r \in \{h = \text{high}, l = \text{low}\}$ and $i \in \{0, 1\}$. These rates $(\alpha_{i\cdot}^{r\cdot})$ can be used to express the different α_{ij}^{qr} for different mixing assumptions.

Before we give the equations of the observable $\alpha_{i\cdot}^{r\cdot}$ in terms of the sought α_{ij}^{qr}, we will present some symmetry arguments which reduces the number of free parameters. To begin with, we have 16 different parameters α_{ij}^{rq}. For symmetry reasons, when disregarding the activity degrees, the total rate in the population at which singles have casual sex with individuals in a partnership needs to equal the rate at which individuals in partnership have casual sex with singles. Assume that we have a population of size $n = n_0+n_1$, where $n_0 = nP_0$ is the number of individuals without a steady partner and $n_1 = nP_1$ is the number with a steady partner. A similar consistency criterion as for the rate of casual contacts disregarding heterogeneity in activity degree must also hold for the model with two activity degrees: the rate low-active singles ($n_0\pi_l$ in the population) have casual sex with high-active singles must equal the rate high-active singles ($n_0\pi_h$ in the population) have casual sex with low-active singles, i.e.

$$n_0\pi_l\alpha_{00}^{lh}P_0\pi_h = n_0\pi_h\alpha_{00}^{hl}P_0\pi_l,$$

which simplifies to

$$\alpha_{00}^{lh} = \alpha_{00}^{hl}.$$

Consistency hence require that

$$\alpha_{11}^{lh} = \alpha_{11}^{hl}, \quad \alpha_{10}^{lh} = \alpha_{01}^{hl}, \quad \alpha_{01}^{lh} = \alpha_{10}^{hl},$$

and

$$\alpha_{10}^{ll} = \alpha_{01}^{ll}, \quad \alpha_{10}^{hh} = \alpha_{01}^{hh},$$

or in one equation:

$$\alpha_{ij}^{rq} = \alpha_{ji}^{qr}. \tag{S2.1}$$

This reduces the number of free parameters to 10.

Let us now express the rate that an $r-$active individual with i steady partners finds new casual sex partners, $\alpha_{i\cdot}^{r\cdot}$, in terms of α_{ij}^{rq}

$$\begin{cases} \alpha_{0\cdot}^{h\cdot} = (\alpha_{00}^{hl}P_0 + \alpha_{01}^{hl}P_1)\pi_l + (\alpha_{00}^{hh}P_0 + \alpha_{01}^{hh}P_1)\pi_h \\ \alpha_{0\cdot}^{l\cdot} = (\alpha_{00}^{ll}P_0 + \alpha_{01}^{ll}P_1)\pi_l + (\alpha_{00}^{lh}P_0 + \alpha_{01}^{lh}P_1)\pi_h \\ \alpha_{1\cdot}^{h\cdot} = (\alpha_{10}^{hl}P_0 + \alpha_{11}^{hl}P_1)\pi_l + (\alpha_{10}^{hh}P_0 + \alpha_{11}^{hh}P_1)\pi_h \\ \alpha_{1\cdot}^{l\cdot} = (\alpha_{10}^{ll}P_0 + \alpha_{11}^{ll}P_1)\pi_l + (\alpha_{10}^{lh}P_0 + \alpha_{11}^{lh}P_1)\pi_h. \end{cases} \tag{S2.2}$$

This system of equations can later together with a proportionate mixing or complete assortativity assumption be solved, the solutions can be found in Section S2.1 and section S2.1, respectively.

For the case of an assortativity between the proportionate mixing and complete assortativity, we need further information than the $\alpha_{i\cdot}^{r\cdot}$ provides. With the help of a proxy question on participants' partners sexual behaviour (explained in Section S3.5), we can additionally estimate more detailed rates than $\alpha_{i\cdot}^{r\cdot}$: the rate

For peer review only - http://bmjopen.bmj.com/site/about/guidelines.xhtml

that an $r-$active individual with i steady partners finds new casual sex partners that are $q-$active, $\alpha_{i\cdot}^{rq}$. The following hold for these rates

$$\begin{cases} \alpha_{0\cdot}^{hh} = (\alpha_{00}^{hh} P_0 + \alpha_{01}^{hh} P_1)\pi_h \\ \alpha_{0\cdot}^{hl} = (\alpha_{00}^{hl} P_0 + \alpha_{01}^{hl} P_1)\pi_l \\ \alpha_{0\cdot}^{lh} = (\alpha_{00}^{lh} P_0 + \alpha_{01}^{lh} P_1)\pi_h \\ \alpha_{0\cdot}^{ll} = (\alpha_{00}^{ll} P_0 + \alpha_{01}^{ll} P_1)\pi_l \\ \alpha_{1\cdot}^{hh} = (\alpha_{10}^{hh} P_0 + \alpha_{11}^{hh} P_1)\pi_h \\ \alpha_{1\cdot}^{hl} = (\alpha_{10}^{hl} P_0 + \alpha_{11}^{hl} P_1)\pi_l \\ \alpha_{1\cdot}^{lh} = (\alpha_{10}^{lh} P_0 + \alpha_{11}^{lh} P_1)\pi_h \\ \alpha_{1\cdot}^{ll} = (\alpha_{10}^{ll} P_0 + \alpha_{11}^{ll} P_1)\pi_l. \end{cases} \tag{S2.3}$$

This gives us 8 equations with 10 unknowns. The data does not provide information on whether a casual sex partner is single or in a steady partnership. Therefore, we need to make further assumptions concerning the relation (ratio) between the rate of finding a casual sex partner that are single and the rate of finding a casual sex partner that are in a steady partnership. Let us consider one high-active individual, we assume that the rate of casual contact with a high-active in a partnership compared to the rate with a high-active single, is the same regardless if the considered individual is in a partnership or not, i.e.

$$\frac{\alpha_{00}^{hh}}{\alpha_{01}^{hh}} = \frac{\alpha_{10}^{hh}}{\alpha_{11}^{hh}}, \tag{S2.4}$$

and similarly, for a low-active individual (finding another low-active)

$$\frac{\alpha_{00}^{ll}}{\alpha_{01}^{ll}} = \frac{\alpha_{10}^{ll}}{\alpha_{11}^{ll}}. \tag{S2.5}$$

However, it turns out that the two equations are linearly dependent, we therefore need one more equation to be able to solve the system of equations (S2.3). We make the same kind of assumption but for the case when a high-active meets a low-active: for a high-active individual, the rate of casual contact with a low-active in a partnership compared to the rate with a low-active being single is the same regardless if the high-active individual is in a partnership or not

$$\frac{\alpha_{00}^{hl}}{\alpha_{01}^{hl}} = \frac{\alpha_{10}^{hl}}{\alpha_{11}^{hl}}, \tag{S2.6}$$

which further implies that $\frac{\alpha_{00}^{lh}}{\alpha_{01}^{lh}} = \frac{\alpha_{10}^{lh}}{\alpha_{11}^{lh}}$.

From the consistency criteria given in Equation (S2.1), Equation (S2.4)-(S2.6) the system of Equations (S2.3) can therefore be solved. Let $D_{qr} = \pi_q(\alpha_{0\cdot}^{qr} P_0 + \alpha_{1\cdot}^{qr} P_1)$, then we can express the rate for a $q-$active to try to find a r-active as:

$$\alpha_{ij}^{qr} = \frac{\alpha_{i\cdot}^{qr}\alpha_{j\cdot}^{rq}}{D_{qr}}. \tag{S2.7}$$

Note that, by consistency

$$D_{hl} = \pi_h(\alpha_{0\cdot}^{hl} P_0 + \alpha_{1\cdot}^{hl} P_1) = \pi_l(\alpha_{0\cdot}^{lh} P_0 + \alpha_{1\cdot}^{lh} P_1) = D_{lh}. \tag{S2.8}$$

To conclude, the consistency criteria given in Equation (S2.1), that $D_{hl} = D_{lh}$, together with the assumption given in Equation (S2.4)-(S2.6) implies that we can write α_{ij}^{qr} as a product:

$$\alpha_{ij}^{qr} = \omega_i^{qr}\omega_j^{rq}, \tag{S2.9}$$

where

$$\omega_i^{qr} = \frac{\alpha_{i\cdot}^{qr}}{\sqrt{D_{qr}}}.$$

For peer review only - http://bmjopen.bmj.com/site/about/guidelines.xhtml

S2.1 Proportionate Mixing with respect to activity degree

Proportionate mixing with respect to activity degree means that an individual has no preference regarding which type, high or low-active, it has casual sex with. An individual chooses at random of the potential casual sex attempts in the population. The fraction of potential high-active and low-active casual sex partners will not only depend on the sizes of the two groups, but also the rates at which they try to find new casual sex partners. If the sizes of the groups would be equal, someone trying to find a new casual sex partner would by chance meet a high-active more often since the high-active try to find a new casual sex partner more often than the low-active. Proportionate mixing then implies that the rate a low-active single has casual sex with a high-active single will be

$$\alpha_{00}^{lh} P_0 \pi_h = \alpha_{0\cdot}^{l\cdot} \times \frac{\alpha_{0\cdot}^{h\cdot} P_0 \pi_h}{\alpha_{0\cdot}^{l\cdot} P_0 \pi_l + \alpha_{0\cdot}^{h\cdot} P_0 \pi_h + \alpha_{1\cdot}^{l\cdot} P_1 \pi_l + \alpha_{1\cdot}^{h\cdot} P_1 \pi_h}$$

i.e. the rate a low-active single has casual sex, times the proportion of all casual sex partners that are from high-active singles. In terms of α_{ij}^{qr} we have that

$$\alpha_{ij}^{qr} = \alpha_{i\cdot}^{q\cdot} \times \frac{\alpha_{j\cdot}^{r\cdot}}{\alpha_{0\cdot}^{l\cdot} P_0 \pi_l + \alpha_{0\cdot}^{h\cdot} P_0 \pi_h + \alpha_{1\cdot}^{l\cdot} P_1 \pi_l + \alpha_{1\cdot}^{h\cdot} P_1 \pi_h}$$

The expression for α_{ij}^{qr} can also be found by using that proportionate mixing implies that $\omega_i^{lh} = \omega_i^{ll}$ and $\omega_i^{hh} = \omega_i^{hl}$. By dropping the second superscript and simply write ω_i^l and ω_i^h, yields that

$$\alpha_{ij}^{qr} = \omega_i^q \omega_j^r.$$

This together with the system of equations (S2.2) gives the above solution for α_{ij}^{rq}.

S2.2 Complete assortativity

Complete assortativity in whom you choose to have casual sex with regarding activity-degree implies that no casual sex occurs between high and low active: $\alpha_{ij}^{hl} = 0$. With Equation (S2.9) the system of Equations in (S2.2) can be written as

$$\begin{cases} \alpha_{0\cdot}^{h\cdot} = (\alpha_{00}^{hh} P_0 + \alpha_{01}^{hh} P_1)\pi_h \\ \alpha_{0\cdot}^{l\cdot} = (\alpha_{00}^{ll} P_0 + \alpha_{01}^{ll} P_1)\pi_l \\ \alpha_{1\cdot}^{h\cdot} = (\alpha_{10}^{hh} P_0 + \alpha_{11}^{hh} P_1)\pi_h \\ \alpha_{1\cdot}^{l\cdot} = (\alpha_{10}^{ll} P_0 + \alpha_{11}^{ll} P_1)\pi_l \end{cases} \overset{S2.9}{\iff} \begin{cases} \alpha_{0\cdot}^{h\cdot} = \omega_0^{hh}(\omega_0^{hh} P_0 + \omega_1^{hh} P_1)\pi_h \\ \alpha_{0\cdot}^{l\cdot} = \omega_0^{ll}(\omega_0^{ll} P_0 + \omega_1^{ll} P_1)\pi_l \\ \alpha_{1\cdot}^{h\cdot} = \omega_1^{hh}(\omega_0^{hh} P_0 + \omega_1^{hh} P_1)\pi_h \\ \alpha_{1\cdot}^{l\cdot} = \omega_1^{ll}(\omega_0^{ll} P_0 + \omega_1^{ll} P_1)\pi_l \end{cases}$$

with the solution

$$\omega_i^{rr} = \frac{\alpha_{i\cdot}^{r\cdot}}{\sqrt{\alpha_{0\cdot}^{r\cdot} P_0 \pi_r + \alpha_{1\cdot}^{r\cdot} P_1 \pi_r}}.$$

For example, the rate a high-active single finds new casual sex partners that also are high-active, but in a partnership, becomes

$$\alpha_{01}^{hh} P_1 \pi_h = \omega_0^{hh} \omega_1^{hh} P_1 \pi_h = \alpha_{0\cdot}^{h\cdot} \times \frac{\alpha_{1\cdot}^{h\cdot} P_1 \pi_h}{\alpha_{0\cdot}^{h\cdot} P_0 \pi_h + \alpha_{1\cdot}^{h\cdot} P_1 \pi_h} = \alpha_{0\cdot}^{h\cdot} \times \frac{\alpha_{1\cdot}^{h\cdot} P_1}{\alpha_{0\cdot}^{h\cdot} P_0 + \alpha_{1\cdot}^{h\cdot} P_1}.$$

For peer review only - http://bmjopen.bmj.com/site/about/guidelines.xhtml

S2.3 Mixing determined by the proxy question

To obtain the different α_{ij}^{qr} for the case of an assortativity between the proportionate mixing case and complete assortativity, we use the 8 different $\alpha_{i\cdot}^{qr}$ estimated from data via the proxy question (see Table S3) and Equation S2.9 to get the 16 α_{ij}^{qr}, i.e.

$$\alpha_{ij}^{qr} = \omega_i^{qr}\omega_j^{rq} = \frac{\alpha_{i\cdot}^{qr}\alpha_{j\cdot}^{rq}}{D_{qr}}.$$

S3 Data and parameter estimates

We will here describe the data gathering, calibration of the model and the parameter estimates obtained from the STI-clinic.

S3.1 Data description

The data used in this study was gathered at a gay-friendly STI-testing clinic in Stockholm, Sweden. Collection of data took place between February 2 and December 15, 2015. Participants first reported demographic information and the total number of sex partners during the last 12 months, then the participants were asked to fill in an app-based timeline follow-back (TLFB) questionnaire.

In the TLFB questionnaire participants were asked to mark up to 10 of their most recent sex partners on a 12-month timeline. Participants did themselves label their partners into one of four partnership types: 1) casual unknown sex partner, 2) casual known sex partner, 3) regular sex partner (regular sex partner but not a 'love' relationship), and 4) main sex partner (a loving relationship, e.g. boyfriend/husband). For casual sex partners, a partner was represented by a single point on the interactive timeline, and a steady sex partner was represented by marking the start and end date of the relationship. For each sex partner on the timeline the participants could report: the partnership type 1) to 4); age of partner; frequency of each sex type (oral/anal; receptive/insertive); frequency of condom use; if the sex took place in Sweden or abroad; drug use and transactions in connection to sex with each partner; and if the participant believed the sex partner had other sex partners concurrently. This last question on concurrency is by us here referred to as the proxy question (for activity-degree assortativity).

In total 403 participants completed the TLFB questionnaire, giving detailed information on 2112 different sex partners. However, for a participant to be included in this study the total number of sex partners and the proxy question need to be answered, as explained in the main manuscript, yielding the data-set of this study consisting of 368 participants and 1903 partners.

S3.2 Scaling of the rate of finding a new casual sex partner

Participants reported their total number of sex partners during a year. The maximum number of sex partners of a participant was 250. When dividing the population into a category of high-active (≥ 15 sex partners a year) and one category low-active (< 15 sex partners a year), 124 (33.7%) participants are defined as high-active and 244 (66.3%) are defined as low-active. The mean number of sex partners of high-actives is 33.21 (median 25, sd 32), and the mean number of low-actives is 5.96 (median 5, sd 3.2). The assumption that only the number of casual sex partners is affected by activity-degree is supported by our data: the mean number of steady partners for high-actives and low-actives is 1.37 and 1.39 respectively.

Additionally, the participants gave detailed information on their (up to) 10 most recent of these sex partners. Participants reported what type of partner these 10 most recent sex partners were, either casual or steady, and the timings of these partners on a timeline. As mentioned, a casual sex partner was reported as a cross on the timeline representing the date of sex and a steady sex partner was given by the start date and end date of the relationship. This timeline data is used to estimate, for example, the time until a new casual sex partner. However, the timeline data only considers data on up to 10 casual sex partners, when we in fact know that many participants have more than this. We therefore need to scale the rates of finding a new casual sex partner according to the total number of partners reported by the participants.

For peer review only - http://bmjopen.bmj.com/site/about/guidelines.xhtml
For peer review only

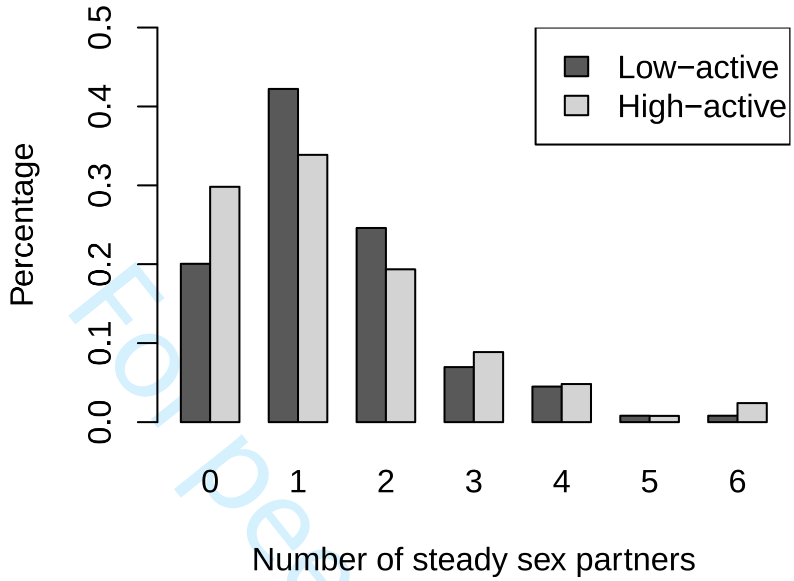

Figure S2: Distribution of steady sex partners of high-actives and low-actives, respectively.

The distribution of the number of steady sex partners of high-actives and low-actives are shown in Figure S2, as seen the distributions are similar. The mean number of steady sex partners per year of a high-active is 1.37 (sd 1.38), from which we estimate that the mean number of casual sex partners is $\mu_h = 31.84$ (total number - number of steady). The mean number of steady sex partners of a low-active is 1.39 (sd 1.15) and the mean number of casual sex partners is therefore $\mu_l = 4.57$. We use μ_h and μ_l to scale the rates of finding casual sex partners, since the detailed data from where timings of casual sex partners was given only include (up to) the 10 most recent sexual partners. In the detailed data, the mean number of casual sex partners is 5.35 for high-active individuals and 3.12 for low-active individuals. Hence the scaling factor will be $\mu_h/5.35$ for high-active individuals and $\mu_l/3.12$ for low-active individuals.

Finally, participants reported to have (a mean value of) 1.4 sex acts with each casual sex partner.

S3.3 Time since last HIV-test

In Table S2 the time since the last HIV-test are shown, indicating that sexually high-active participants test themselves more often than sexually low-active participants. The rate to successful ART-treatment is denoted γ_h and γ_l for high-actives and low-actives, respectively. Calculating ML-estimates of the time since the last HIV-test, assuming an exponential distribution, we find that the testing rate of a high-active is 2.349 higher than the testing rate of a low-active. We will further assume that the same relationship holds for the time to successful ART-treatment, i.e. the rate of initiating successful ART-treatment for an infected high-active is 2.349 times higher than the rate of initiating ART-treatment for an infected low-active. Therefore, we fix $\gamma_h = 2.349\gamma_l$ such that we only need to vary one of the parameters.

S3.4 Distribution of parameters

The time durations in the model, such as the time until finding a new steady sex partner, are assumed to be exponential and are hence specified by their rates. With this assumption we calculate a point estimate and its standard error. In general, if we are looking at something occurring at an exponential rate, α say, then

For peer review only - http://bmjopen.bmj.com/site/about/guidelines.xhtml

Table S2: **Time since last HIV-test separated by degree of activity.**

	High	(%)	Low	(%)	Σ
< 6 mths ago	85	(68.5)	121	(49.6)	206
6 to 12 mths ago	20	(16.1)	54	(22.1)	74
1 to 5 yrs ago	9	(7.3)	35	(14.3)	44
> 5 yrs ago	0	(0)	11	(4.5)	11
No answer	10	(8.1)	14	(5.7)	24
Do not remember	0	(0)	2	(0.1)	2
Σ	124	(100)	244	(100)	368

the number of events N occurring during a time interval of length t is Poisson distributed with parameter αt ($N \sim Poisson(\alpha t)$). Observing n events during a time t leads to the ML-estimate $\hat{\alpha} = n/t$. The variance of the estimate equal $Var(\hat{\alpha}) = Var(N/t) = Var(N)/t^2 = \alpha/t$, which leads to a standard error of the estimate of $\sqrt{\hat{\alpha}/t}$.

For example, in calculating the estimate of the rate for a high-active in a steady partnership to find a high-active casual sex partner, we do as follows: find the total time high-active individuals are in a steady partnership (T_1^h), then find the number of casual sex partners that occur during that time that is with someone that also is high-active (N_1^{hh}) and multiply it with the scaling factor from Section S3.2 ($N_1^{hh} \times \frac{\mu_h}{5.35}$). The point estimate is given by $\hat{\alpha}_{1\cdot}^{hh} = \left(N_1^{hh} \times \frac{\mu_h}{5.35}\right)/T_1^h$ and its standard error by $s.e.(\hat{\alpha}_{1\cdot}^{hh}) = \sqrt{\hat{\alpha}_{1\cdot}^{hh}/T_1^h}$.

For the number of occurrences of anal intercourses (AIs) in a steady partnership, the participants reported the number of acts during a 1-month period. Let m be the number of steady partners among all participants and let a_i denote the number of occurrences of AI with partner $i = 1, \ldots, m$. The estimated rate of AI, in units of months, is

$$\hat{\lambda} = \frac{\sum_{i=1}^{m} a_i}{\sum_{i=1}^{m} 1} = \frac{\sum_{i=1}^{m} a_i}{m},$$

and the standard error is

$$s.e(\hat{\lambda}) = \sqrt{\frac{\hat{\lambda}}{m}}.$$

In the data for casual sex partners, it is recorded if a condom was used (1) or not used (0) during receptive anal intercourse (RAI) and during insertive anal intercourse (IAI). To estimate the condom use in casual contacts we use a Bernoulli assumption and calculate the mean condom use during RAI and IAI, respectively. With a Bernoulli assumption we mean that, in each new casual sex act a condom is used with a probability, p_c say, independently of previous sex acts. Then the estimate $\hat{p}_c$ is given by the mean number times a condom was used. The standard error is given by

$$s.e(\hat{p}_c) = \sqrt{\frac{\hat{p}_c(1-\hat{p}_c)}{n}},$$

where n here is the number of observations, i.e. the number of casual sex partners where a binary response on condom use was given.

For condom use with a steady sex partner, participants could choose from a five-degree scale on how often a condom was used during RAI and during IAI: always (100%), often (75%), half of the times (50%), seldom (25%), and never (0%). Here, the participants did themselves, in a sense, give the mean number of times they used condom with a partner. Assume the data consist of m such steady partners with corresponding responses $(y_1, \ldots, y_m)$. The estimated condom use in steady partnerships, $\hat{p}_s$, is then the mean of the m reported values on the five-degree scale, and the distribution of $\hat{p}_s$ is approximated by the normal distribution

$$\hat{p}_s = \frac{1}{m}\sum_{i=1}^{m} y_i \approx N(p_s, \tau^2/m).$$

For peer review only - http://bmjopen.bmj.com/site/about/guidelines.xhtml

Table S3: **Estimates of partnership and epidemic parameters.**

**Partnership parameters from data**			
Parameter	Estimate	S.E.	Definition
$\sigma + 2\mu$	1.344/year	0.070	Rate of ending steady partnership
ρP_0	2.389/year	0.164	Rate of acquiring new steady partner
λ^*	29.793/year	0.837	Rate of sex acts (AI) within steady partnership
$\hat{\alpha}^h_{0\cdot}$	34.169/year	0.722	
$\hat{\alpha}^h_{1\cdot}$	29.227/year	0.707	
$\hat{\alpha}^l_{0\cdot}$	5.490/year	0.221	
$\hat{\alpha}^l_{1\cdot}$	3.729/year	0.168	
$\hat{\alpha}^{hh}_{0\cdot}$	25.809/year	0.628	
$\hat{\alpha}^{hl}_{0\cdot}$	8.360/year	0.357	
$\hat{\alpha}^{hh}_{1\cdot}$	23.728/year	0.637	
$\hat{\alpha}^{hl}_{1\cdot}$	5.499/year	0.307	
$\hat{\alpha}^{lh}_{0\cdot}$	3.338/year	0.172	
$\hat{\alpha}^{ll}_{0\cdot}$	2.152/year	0.138	
$\hat{\alpha}^{lh}_{1\cdot}$	2.682/year	0.143	
$\hat{\alpha}^{ll}_{1\cdot}$	1.046/year	0.089	
q^{RAI}_s	51.9%	2.4%	Condom use steady partner RAI
q^{IAI}_s	56.2%	2.3%	Condom use steady partner IAI
$\%RAI_s$	49%	-	Percentage of steady RAI and IAI acts that are RAI
q^{RAI}_c	62.8%	2.5%	Condom use casual partner RAI
q^{IAI}_c	63.1%	2.6%	Condom use casual partner IAI
$\%RAI_c$	52%	-	Percentage of casual RAI and IAI partners that are RAI

Note AI: anal intercourse; RAI: receptive anal intercourse; IAI: insertive anal intercourse

Where p_s is the true expected value of the condom use in steady partnerships and τ is the standard deviation of the condom use. The estimate of τ^2 is $\frac{1}{m-1}\sum_{i=1}^{m}(y_i - \hat{p}_s)^2$. The standard error of the estimated condom use with steady sex partners is then given by

$$s.e.(\hat{p}_s) = \hat{\tau}/\sqrt{m} = \sqrt{\frac{\frac{1}{m-1}\sum_{i=1}^{m}(y_i - \hat{p}_s)^2}{m}}.$$

With the estimated condom use during RAI and during IAI, we calculated the overall mean condom use by taking the weighted average of these two estimates. See Table S3 for condom use estimates and the proportion (weights) of sex acts that are RAI and IAI, from these values we can calculate the overall condom use for steady partners

$$q_s = q^{RAI}_s \times \%RAI_s + q^{IAI}_s \times \%IAI_s = 0.519 \cdot 0.49 + 0.562 \cdot 0.51 = 0.541,$$

and for casual sex partners

$$q_c = q^{RAI}_c \times \%RAI_c + q^{IAI}_c \times \%IAI_c = 0.628 \cdot 0.52 + 0.631 \cdot 0.48 = 0.629.$$

For peer review only - http://bmjopen.bmj.com/site/about/guidelines.xhtml

S3.5 Proxy of partners' activity degree

For the 10 most recent sex partners the participants responded to detailed questions. One of the detailed questions was what we referred to as the proxy question: 'Do you think that your sex partner had other sex partners than you during the same time frame that he/she met you?', with the possible answers

1. I know this person had sex with others
2. I think this person had sex with others
3. No, this person only had sex with me
4. I don't know

The percentages of the answers to the proxy question are given in Table S4.

Table S4: **Distribution of proxy variable on partners' activity degree.** One of the questions participants answered concerning their 10 most recent sex partners was whether they believed their partners had other concurrent sex partners. We use this as a proxy for partners activity-degree, as shown in the fourth and last column. With the help of a consistency criterion, Equation (S2.8), we assigned the partners of answer 4 as low-active.

Answer	Of all partners ($n = 1903$)	Of all casual partners ($n = 1424$)	Proxy for partner being
1. Yes	33.58%	32.65%	High-active
2. Think so	33.63%	38.76%	High-active
3. No	9.98%	3.30%	Low-active
4. Don't know	22.81%	25.28%	Low-active

If a participant answered either 1 or 2 for a partner, we will take this as a proxy for that the partner is high-active. If a participant answered 3, we will use this as a proxy for that the partner is low-active. We now need to decide what to do with the 25% of the partners that participants labelled as No. 4 on the proxy question, the partners that participants did not know whether they had other sex partners.

In the total population, consistency requires that the the number of casual sex acts high-actives have with low-actives needs to equal the number of casual sex acts low-actives have with high-actives. This criterion can be written, in terms of rates, as (Equation (S2.8))

$$D_{hl} = D_{lh}.$$

With this consistency we get help in determining how to assign the partners labelled as No. 4 on the proxy question. If we simply remove these partners entirely, the left-hand side of Equation (S2.8) becomes 0.18 and the right-hand side 1.93, very far from each other. Hence, to remove the partners of which the participants do not know (No. 4) yields a too big inconsistency. If we instead assign all these partners as low-active, we get that the left-hand side of Equation (S2.8) becomes 2.19 the right-hand side 1.93. This suggests that many of the partners participants labelled as No. 4 should be categorised as low-active.

In Table S5 we show (for the two choices of actions of answer No. 4 "I don't know"), the proportion of high-active individuals' casual sex partners that will be with low-actives and with high-actives, respectively; and the proportion of low-active individuals' casual sex partners that will be with low-actives and with high-actives, respectively. For example, if we assign all partners that participants labelled as No. 4 on the proxy question as low-active, we find that 34.3% of low-active individuals' casual sex partners will be with low-actives, and 65.7% will be with high-actives.

For peer review only - http://bmjopen.bmj.com/site/about/guidelines.xhtml

Table S5: **Consequence of the two assignments of the partners of which participants do not know if the partner did have other partners.** The column named *Removed* means that the partners of participants of which we do not know (answer 4 on the proxy question) were removed, and the column *As low-active* means we assigned those partners as low-active. First, for the two assignments, we show the proportion of low-active and high-active partners of participants who are low-active, then the same kind of proportion but for participants who are high-active. Then we show the values of the right-hand side (D_{lh}) and left-hand side D_{hl} of the consistency criterion Equation (S2.8)

	**Action on "Don't know"**	
Proportion	Removed	As low-active
low-low	0.067	0.343
low-high	0.933	0.657
high-low	0.021	0.220
high-high	0.979	0.780
Consistency		
D_{lh}	1.9345	1.9345
D_{hl}	0.1825	2.19

S3.6 Final estimates of the rates of acquiring new casual sex partners

We will now show the final estimates of the rates of finding new casual sex partners, using the three different mixing assumptions with respect to activity degree.

We found that the estimates for α_{ij}^{qr} that utilises the proxy question could be written as Equation S2.9

$$\alpha_{ij}^{qr} = \omega_i^{qr}\omega_j^{rq} = \frac{\alpha_{i\cdot}^{qr}\alpha_{j\cdot}^{rq}}{D_{qr}} = \frac{\alpha_{i\cdot}^{qr}\alpha_{j\cdot}^{rq}}{\pi_q(\alpha_{0\cdot}^{qr}P_0 + \alpha_{1\cdot}^{qr}P_1)},$$

where consistency requires that $D_{hl} = D_{lh}$ (Equation (S2.8)) needs to be fulfilled. Utilising Table S5, we found that assigning all partners that participants answered "I don't know" (answer No. 4) as low-active on the proxy question yielded a value of 2.19 for the left-hand side and a value of 1.93 of the right-hand side of Equation (S2.8), i.e. similar but not equal. We choose to work with the left-hand side, setting $D_{hl} = D_{lh} = 2.19$ when estimating the different α_{ij}^{qr}, which can be seen in Table S6. To make the comparison between the different mixing assumptions, we also use that $D_{hl} = D_{lh} = 2.19$ for all mixing assumptions. In Table S6 we show the estimates for proportionate and complete mixing under the assumption that $D_{hl} = D_{lh}$, where the values in parenthesis are the ones not requiring that $D_{hl} = D_{lh}$.

To quantify the degree of assortativity (with respect to activity degree) as a value θ between 0 and 1, where $\theta = 0$ means proportionate mixing and $\theta = 1$ means complete assortativity, we write

$$\alpha_{proxy}^{qr} = (1-\theta)\alpha_P^{qr} + \theta\alpha_C^{qr}.$$

Where α_P^{qr} is the rate, disregarding partnership status, a q-active tries to find an r-active under proportionate mixing and α_C^{qr} under complete assortativity. These α_{proxy}^{qr}, α_P^{qr}, and α_C^{qr} can be found by calculating the following quantity where α_{ij}^{qr}, $i, j \in 0, 1$ is taken from the corresponding mixing assumption,

$$\alpha_X^{qr} = \alpha_{11}^{qr}P_1^2 + (\alpha_{01}^{qr} + \alpha_{10}^{qr})P_0P_1 + \alpha_{00}^{qr}P_0^2.$$

Where P_0 is the proportion of the population without a steady sex partner and P_1 the proportion with a steady sex partner. As examples, using table S6 and the estimated value for $P_0 = 0.36$ and $P_1 = 0.64$, we get

$$\begin{aligned}
\alpha_P^{hh} &= 62.20 \cdot P_1^2 + (72.44 + 72.44)P_0P_1 + 84.10 \cdot P_0^2 = 69.8,\\
\alpha_C^{hh} &= 80.13 \cdot P_1^2 + (93.02 + 93.02)P_0P_1 + 108.00 \cdot P_0^2 = 89.7,\\
\alpha_{proxy}^{hh} &= 68.26 \cdot P_1^2 + (74.25 + 74.25)P_0P_1 + 80.76 \cdot P_0^2 = 72.6.
\end{aligned}$$

For peer review only - http://bmjopen.bmj.com/site/about/guidelines.xhtml

Table S6: **Estimates of rate parameters of finding new casual sex partner (years).** For the two extreme cases, proportionate mixing and complete assortativity, we give in parenthesis the casual sex rate estimates not requiring that $D_{hl} = D_{lh}$. Note that $\hat{\alpha}_{01}^{qr} = \hat{\alpha}_{10}^{rq}$ and is therefore not explicitly presented.

Parameter	Proxy	Prop	Complete
$\hat{\alpha}_{11}^{hh}$	68.26	62.20 (64.03)	80.13 (81.76)
$\hat{\alpha}_{11}^{hl}$	6.70	8.15 (8.17)	-
$\hat{\alpha}_{11}^{lh}$	6.70	8.15 (8.17)	-
$\hat{\alpha}_{11}^{ll}$	1.14	1.06 (1.04)	4.81 (4.81)
$\hat{\alpha}_{10}^{hh}$	74.25	72.44 (74.85)	93.02 (95.58)
$\hat{\alpha}_{10}^{hl}$	8.34	11.99 (12.03)	-
$\hat{\alpha}_{10}^{lh}$	10.19	9.46 (9.55)	-
$\hat{\alpha}_{10}^{ll}$	2.35	1.57 (1.53)	7.08 (7.08)
$\hat{\alpha}_{00}^{hh}$	80.76	84.10 (87.52)	108.00 (111.75)
$\hat{\alpha}_{00}^{hl}$	12.69	13.92 (14.06)	-
$\hat{\alpha}_{00}^{lh}$	12.69	13.92 (14.06)	-
$\hat{\alpha}_{00}^{ll}$	4.83	2.30 (2.26)	10.42 (10.42)

The value of θ that corresponds to these values is 0.141. Doing the same kind of calculations but for α^{lh} and α^{ll} yields the same θ.

Note that, there could exist disassortative with respect to activity-degree, however, we disregard from this since it seems unlikely in our application.

For peer review only

For peer review only - http://bmjopen.bmj.com/site/about/guidelines.xhtml

S4 Finding the endemic prevalence, a deterministic approximation

Both the basic reproduction number and the endemic level can be obtained by using a deterministic approximation of the stochastic model. Assuming that the population is large, it is then enough to consider expected values of the fraction of individuals that are susceptible, infectious, or recovered to obtain these quantities. In the following section, we explain how we construct the compartments and give the differential equations governed by the possible transitions within the network model. To find the endemic (or equilibrium) prevalence one needs to find the non-trivial steady state of the system of differential equations. The trivial solution is that everyone is susceptible.

In Figure S1 we showed the different infectious states an individual can be in; the four different infectious states are susceptible S, acute infectious A, chronic infectious C, and treated (on ART-treatment) T. We further divide the population into different types, these types specify: if an individual is single or in a partnership, if the individual is low-active or high-active in having casual sex partners, the infectious state of the individual, and the partner's infectious state. We will study the fraction of the population belonging to each type, each individual will therefore contribute with $1/n$ to the type it belongs to.

The fraction of all individuals that are susceptible, single and r-active is denoted by S_0^r; the fraction of all individuals that are single, r-active, and infectious in the acute stage is denoted by A_0^r; the fraction of all individuals that are single, r-active, and infectious in the chronic stage is denoted by C_0^r; and recovered singles (on ART-treatment) that are r-active is denoted by T_0^r.

Let $\mathcal{X} = \{S, A, C, T\}$ be the set of possible states not including PrEP, let $\mathcal{X_P} = \{SP, AP, CP\}$ be the possible states when being on PrEP, and let $\mathcal{D} = \{l, h\}$ be the set of possible activity degrees with regards to the casual contacts. Furthermore, let X_Y^{rq} denote the fraction of individuals that are r-active of type $X \in \mathcal{X} \cup \mathcal{X_P}$, with a q-active partner of type $Y \in \mathcal{X} \cup \mathcal{X_P}$. Note that, this counts each individual in the fraction X_Y^{rq}, not each pair. E.g. S_S^{rq} is the fraction of all individuals that are susceptible r-active and in a partnership with a q-active susceptible. The reason for taking this individual-based perspective is that the data is individual based. Moreover, the individual-based perspective makes it simpler to extend the model by allowing for more than one steady partner at a time in future work.

Disregarding the use of PrEP, there are in total 8 different single types and 64 types of partnerships (hence 72 equations). Some of these types' fraction must by consistency be equal, namely

- the 4 equations $X_X^{hl} = X_X^{lh}$ where $X \in \mathcal{X}$
- the 12 equations $X_S^{qr} = S_X^{rq}$ where $X \in \{A, C, T\}$ and $q, r \in \mathcal{D}$,
- the 8 equations $X_A^{qr} = A_X^{rq}$ where $X \in \{C, T\}$ and $q, r \in \mathcal{D}$,
- the 4 equations $T_C^{qr} = C_T^{rq}$ where $q, r \in \mathcal{D}$,

which reduces the number of equations to 44.

Including the use of PrEP for high-actives creates: 3 more single types (denoted SP_0^h, AP_0^h, and CP_0^h); 48 partnership types between one participant on PrEP and one not on PrEP; and 9 partnership types where both participants in the steady partnership are on PrEP. Hence, introducing PrEP increases the number of types by 60; however, some of the PrEP types' fraction must also by consistency be equal

- the 8 equations $X_{SP}^{qh} = SP_X^{hq}$ where $X \in \mathcal{X}$ and $q \in \mathcal{D}$,
- the 8 equations $X_{AP}^{qh} = AP_X^{hq}$ where $X \in \mathcal{X}$ and $q \in \mathcal{D}$,
- the 8 equations $X_{CP}^{qh} = CP_X^{hq}$ where $X \in \mathcal{X}$ and $q \in \mathcal{D}$,
- $SP_{AP}^{hh} = AP_{SP}^{hh}$,
- $SP_{CP}^{hh} = CP_{SP}^{hh}$,
- $AP_{CP}^{hh} = CP_{AP}^{hh}$.

For peer review only - http://bmjopen.bmj.com/site/about/guidelines.xhtml

Introducing PrEP increases the number of equations needed to be specified by 33, from 44 to 77.

There are some additional facts that will reduce the number of equations. Recall that the fraction without a steady partner is denoted P_0, the fraction with a steady partner is denoted P_1, and the fraction high-active individuals and low-active individuals in the population are denoted π_h and π_l, respectively. The following two constrains concerning singles must hold:

$$\begin{aligned}\pi_l P_0 &= S_0^l + A_0^l + C_0^l + T_0^l,\\ \pi_h P_0 &= S_0^h + SP_0^h + A_0^h + AP_0^h + C_0^h + CP_0^h + T_0^h.\end{aligned} \tag{S4.1}$$

The following three constrains for individuals in steady partnerships must hold. (I) The fraction of the population that is low-active in a steady partnership with a low-active is

$$\begin{aligned}\pi_l^2 P_1 &= S_S^{ll} + S_A^{ll} + S_C^{ll} + S_T^{ll}\\ &+ A_S^{ll} + A_A^{ll} + A_C^{ll} + A_T^{ll}\\ &+ C_S^{ll} + C_A^{ll} + C_C^{ll} + C_T^{ll}\\ &+ T_S^{ll} + T_A^{ll} + T_C^{ll} + T_T^{ll}\\ &= \sum_{X\in\mathcal{X}}\sum_{Y\in\mathcal{X}} X_Y^{ll}.\end{aligned}$$

That is, we sum over all possible states $X \in \mathcal{X}$, the first low-active individual in the relationship can have, and over all possible states that $Y \in \mathcal{X}$, the second low-active individual in the relationship can have. (II) The fraction of the population that is low-active in a steady partnership with a high-active is

$$\begin{aligned}\pi_l\pi_h P_1 &= S_S^{lh} + S_A^{lh} + S_C^{lh} + S_T^{lh} + S_{SP}^{lh} + S_{AP}^{lh} + S_{CP}^{lh}\\ &+ A_S^{lh} + A_A^{lh} + A_C^{lh} + A_T^{lh} + A_{SP}^{lh} + A_{AP}^{lh} + A_{CP}^{lh}\\ &+ C_S^{lh} + C_A^{lh} + C_C^{lh} + C_T^{lh} + C_{SP}^{lh} + C_{AP}^{lh} + C_{CP}^{lh}\\ &+ T_S^{lh} + T_A^{lh} + T_C^{lh} + T_T^{lh} + T_{SP}^{lh} + T_{AP}^{lh} + T_{CP}^{lh}\\ &= \sum_{X\in\mathcal{X}}\left(\sum_{Y\in\mathcal{X}\cup\mathcal{X}_\mathcal{P}} X_Y^{lh}\right),\end{aligned}$$

here we sum over all possible states $X \in \mathcal{X}$, the first low-active individual in the relationship can have, and over all possible states that $Y \in \mathcal{X} \cup \mathcal{X}_\mathcal{P}$, the second high-active individual in the relationship can have. The difference from the previous sum is that a high-active individual can be on PrEP. The fraction of the population that is high-active in a steady partnership with a low-active (which is the same as fraction low with high above) is

$$\pi_h\pi_l P_1 = \sum_{X\in\mathcal{X}\cup\mathcal{X}_\mathcal{P}}\left(\sum_{Y\in\mathcal{X}} X_Y^{hl}\right).$$

(III) The fraction of the population that is high-active in a steady partnership with a high-active is

$$\pi_h^2 P_1 = \sum_{X\in\mathcal{X}\cup\mathcal{X}_\mathcal{P}}\left(\sum_{Y\in\mathcal{X}\cup\mathcal{X}_\mathcal{P}} X_Y^{hh}\right).$$

This leads to that we can reduce the number of equations further, from 77 to 72.

Before we show the system of differential equations that describe our model, we define some quantities that will improve readability. Let us write the fraction of the population that is r-active susceptible in a partnership as S_1^r (where 1 refer to having a steady partner),

$$\begin{aligned}S_1^r &= S_{SP}^{rh} + S_{AP}^{rh} + S_{CP}^{rh} + S_S^{rh} + S_A^{rh} + S_C^{rh} + S_T^{rh} + S_S^{rl} + S_A^{rl} + S_C^{rl} + S_T^{rl}\\ &= S_{SP}^{rh} + S_{AP}^{rh} + S_{CP}^{rh} + \sum_{q\in\mathcal{D}} S_S^{rq} + S_A^{rq} + S_C^{rq} + S_T^{rq}\\ &= \sum_{X\in\mathcal{X}_\mathcal{P}} S_X^{rh} + \sum_{X\in\mathcal{X}}\sum_{q\in\mathcal{D}} S_X^{rq}.\end{aligned}$$

For peer review only - http://bmjopen.bmj.com/site/about/guidelines.xhtml

Similarly, we write the fraction r-active acute infectious individuals in a partnership as A_1^r,

$$A_1^r = \sum_{X\in\mathcal{X}_\mathcal{P}} A_X^{rh} + \sum_{X\in\mathcal{X}}\sum_{q\in\mathcal{D}} A_X^{rq};$$

the fraction r-active chronic infectious individuals in a partnership as C_1^r,

$$C_1^r = \sum_{X\in\mathcal{X}_\mathcal{P}} C_X^{rh} + \sum_{X\in\mathcal{X}}\sum_{q\in\mathcal{D}} C_X^{rq};$$

and the fraction r-active diagnosed and on ART-treatment in a partnership as T_1^r,

$$T_1^r = \sum_{X\in\mathcal{X}_\mathcal{P}} T_X^{rh} + \sum_{X\in\mathcal{X}}\sum_{q\in\mathcal{D}} T_X^{rq}.$$

For the PrEP states we have

$$\begin{aligned}
SP_1^h &= \sum_{X\in\mathcal{X}_\mathcal{P}} SP_X^{hh} + \sum_{X\in\mathcal{X}}\sum_{q\in\mathcal{D}} SP_X^{hq},\\
AP_1^h &= \sum_{X\in\mathcal{X}_\mathcal{P}} AP_X^{hh} + \sum_{X\in\mathcal{X}}\sum_{q\in\mathcal{D}} AP_X^{hq},\\
CP_1^h &= \sum_{X\in\mathcal{X}_\mathcal{P}} CP_X^{hh} + \sum_{X\in\mathcal{X}}\sum_{q\in\mathcal{D}} CP_X^{hq}.
\end{aligned}$$

Remember that the transmission probability in one sex act in the acute stage is denoted by p_A and in the chronic stage by p_C. An r-active individual who is single, susceptible, and not on PrEP acquire infection at rate

$$\begin{aligned}
\beta_0^r = \alpha_{00}^{rh} &\left(p_A(A_0^h + AP_0^h) + p_C(C_0^h + CP_0^h)\right)\\
&+\alpha_{01}^{rh} \left(p_A(A_1^h + AP_1^h) + p_C(C_1^h + CP_1^h)\right)\\
&+\alpha_{00}^{rl}(p_A A_0^l + p_C C_0^l)\\
&+\alpha_{01}^{rl}(p_A A_1^l + p_C C_1^l).
\end{aligned}$$

Similarly, an r-active susceptible not on PrEP with a steady partner acquire infection via casual sex at rate

$$\begin{aligned}
\beta_1^r = \alpha_{10}^{rh} &\left(p_A(A_0^h + AP_0^h) + p_C(C_0^h + CP_0^h)\right)\\
&+\alpha_{11}^{rh} \left(p_A(A_1^h + AP_1^h) + p_C(C_1^h + CP_1^h)\right)\\
&+\alpha_{10}^{rl}(p_A A_0^l + p_C C_0^l)\\
&+\alpha_{11}^{rl}(p_A A_1^l + p_C C_1^l).
\end{aligned}$$

Introducing PrEP will make some susceptible less likely to acquire infection, let us denote the reduction by ζ. Assuming a susceptible is protected by 86% by PrEP yields a value of $\zeta = 1 - 0.86 = 0.14$. A high-active susceptible that is single and on PrEP will acquire infection via casual sex at rate $\zeta\beta_0^h$.

The model can now be described by a set of 72 differential equations. We will begin by specifying all single state equations, followed by the different partnership state equations. We remind the reader of the parameter definitions that can be found in Table S1.

S4.1 Single states

For high-active susceptible singles not on PrEP we have that

$$\frac{dS_0^h}{dt} = \overbrace{\mu\pi_h}^{\substack{\text{Birth of}\\ \text{high-active}}} + \underbrace{(\sigma+\mu)S_1^h}_{\substack{\text{Separation of high-active}\\ \text{from a partner}}} - \overbrace{(\xi+\mu+\rho P_0)S_0^h}^{\substack{\text{High-active single starts PrEP,}\\ \text{dies or enter partnership}}} - \underbrace{\beta_0^h S_0^h}_{\substack{\text{High-active single}\\ \text{acquire infection}}}.$$

For peer review only - http://bmjopen.bmj.com/site/about/guidelines.xhtml

For the other single states, we have

$$\frac{dSP_0^h}{dt} = \xi S_0^h + (\sigma+\mu)SP_1^h - (\mu+\rho P_0)SP_0^h - \zeta\beta_0^h SP_0^h,$$
$$\frac{dA_0^h}{dt} = (\sigma+\mu)A_1^h - (\mu+\gamma_h+\delta_a+\rho P_0)A_0^h + \beta_0^h S_0^h,$$
$$\frac{dAP_0^h}{dt} = (\sigma+\mu)AP_1^h - (\mu+\gamma_P+\delta_a+\rho P_0)AP_0^h + \zeta\beta_0^h SP_0^h,$$
$$\frac{dC_0^h}{dt} = (\sigma+\mu)C_1^h + \delta_a A_0^h - (\mu+\gamma_h+\rho P_0)C_0^h,$$
$$\frac{dCP_0^h}{dt} = (\sigma+\mu)CP_1^h + \delta_a AP_0^h - (\mu+\gamma_P+\rho P_0)CP_0^h.$$

Due to constrain (S4.1), the fraction of the population that is high-active on ART-treatment is equal to

$$T_0^h = \pi_h P_0 - \left(S_0^h + SP_0^h + A_0^h + AP_0^h + C_0^h + CP_0^h\right).$$

In a similar way we get the equations for a low-active single. Note that a low-active never starts to use PrEP; however, we could switch which activity-group is targeted for the intervention.

$$\frac{dS_0^l}{dt} = \mu\pi_l + (\sigma+\mu)S_1^l - (\mu+\rho P_0)S_0^l - \beta_0^l S_0^l,$$
$$\frac{dA_0^l}{dt} = (\sigma+\mu)A_1^l - (\mu+\gamma_h+\delta_a+\rho P_0)A_0^l + \beta_0^l S_0^l,$$
$$\frac{dC_0^l}{dt} = (\sigma+\mu)C_1^l + \delta_a A_0^l - (\mu+\gamma_h+\rho P_0)C_0^l.$$

And the fraction low-active on ART-treatment is equal to $T_0^l = \pi_l P_0 - \left(S_0^l + A_0^l + C_0^l\right)$.

S4.2 Partnership states

We will specify the partnership state equations in the following order: first all possible combinations of a susceptible not on PrEP, S_S, S_A, S_C, S_T, S_{SP}, S_{AP}, S_{CP}; then all possible combinations of an acute infectious individual not on PrEP that has not previously been specified, A_A, A_C, A_T, A_{SP}, A_{AP}, A_{CP}; then all possible combinations of a chronic infectious individual not on PrEP that has not previously been specified, C_C, C_T, C_{SP}, C_{AP}, C_{CP}; then the treated individuals not previously specified, T_T, T_{SP}, T_{AP}, T_{CP}. Then we specify the partnership type equations of individuals on PrEP: for a susceptible on PrEP, SP_{SP}, SP_{AP}, SP_{CP}; for an acute infectious on PrEP, AP_{AP}, AP_{CP}; and finally for a chronic infectious on PrEP, CP_{CP}.

S4.2.1 Susceptible not on PrEP

For susceptible individuals not on PrEP in a partnership with another susceptible not on PrEP we have that

$$\frac{dS_S^{hh}}{dt} = \rho\left(S_0^h\right)^2 - (2\xi+\sigma+2\mu)S_S^{hh} - 2\beta_1^h S_S^{hh},$$
$$\frac{dS_S^{hl}}{dt} = \rho S_0^h S_0^l - (\xi+\sigma+2\mu)S_S^{hl} - (\beta_1^h+\beta_1^l)S_S^{hl},$$
$$\frac{dS_S^{ll}}{dt} = \rho\left(S_0^l\right)^2 - (\sigma+2\mu)S_S^{ll} - 2\beta_1^l S_S^{ll},$$

where $S_S^{lh} = S_S^{hl}$.

For peer review only - http://bmjopen.bmj.com/site/about/guidelines.xhtml

For susceptible individuals not on PrEP in a partnership with an acute infectious individual not on PrEP

$$\frac{dS_A^{hh}}{dt} = \rho S_0^h A_0^h + \beta_1^h S_S^{hh} - (\xi + \lambda p_A + \delta_a + \gamma_h + \sigma + 2\mu) S_A^{hh} - \beta_1^h S_A^{hh},$$
$$\frac{dS_A^{hl}}{dt} = \rho S_0^h A_0^l + \beta_1^l S_S^{hl} - (\xi + \lambda p_A + \delta_a + \gamma_l + \sigma + 2\mu) S_A^{hl} - \beta_1^h S_A^{hl},$$
$$\frac{dS_A^{lh}}{dt} = \rho S_0^l A_0^h + \beta_1^h S_S^{lh} - (\lambda p_A + \delta_a + \gamma_h + \sigma + 2\mu) S_A^{lh} - \beta_1^l S_A^{lh},$$
$$\frac{dS_A^{ll}}{dt} = \rho S_0^l A_0^l + \beta_1^l S_S^{ll} - (\lambda p_A + \delta_a + \gamma_l + \sigma + 2\mu) S_A^{ll} - \beta_1^l S_A^{ll}.$$

Note that $A_S^{rq} = S_A^{qr}$.

For susceptible individuals not on PrEP in a partnership with a chronic infectious individual not on PrEP

$$\frac{dS_C^{hh}}{dt} = \rho S_0^h C_0^h + \delta_a S_A^{hh} - (\xi + \lambda p_C + \gamma_h + \sigma + 2\mu) S_C^{hh} - \beta_1^h S_C^{hh}$$
$$\frac{dS_C^{hl}}{dt} = \rho S_0^h C_0^l + \delta_a S_A^{hl} - (\xi + \lambda p_C + \gamma_l + \sigma + 2\mu) S_C^{hl} - \beta_1^h S_C^{hl},$$
$$\frac{dS_C^{lh}}{dt} = \rho S_0^l C_0^h + \delta_a S_A^{lh} - (\lambda p_C + \gamma_h + \sigma + 2\mu) S_C^{lh} - \beta_1^l S_C^{lh},$$
$$\frac{dS_C^{ll}}{dt} = \rho S_0^l C_0^l + \delta_a S_A^{ll} - (\lambda p_C + \gamma_l + \sigma + 2\mu) S_C^{ll} - \beta_1^l S_C^{ll}.$$

And additionally, $C_S^{rq} = S_C^{qr}$.

For susceptible individuals not on PrEP in a partnership with an individual on ART-treatment

$$\frac{dS_T^{hh}}{dt} = \rho S_0^h T_0^h + \gamma_h (S_A^{hh} + S_C^{hh}) + \gamma_P (S_{AP}^{hh} + S_{CP}^{hh}) - (\xi + \sigma + 2\mu) S_T^{hh} - \beta_1^h S_T^{hh},$$
$$\frac{dS_T^{hl}}{dt} = \rho S_0^h T_0^l + \gamma_l (S_A^{hl} + S_C^{hl}) - (\xi + \sigma + 2\mu) S_T^{hl} - \beta_1^h S_T^{hl},$$
$$\frac{dS_T^{lh}}{dt} = \rho S_0^l T_0^h + \gamma_h (S_A^{lh} + S_C^{lh}) + \gamma_P (S_{AP}^{lh} + S_{CP}^{lh}) - (\sigma + 2\mu) S_T^{lh} - \beta_1^l S_T^{lh},$$
$$\frac{dS_T^{ll}}{dt} = \rho S_0^l T_0^l + \gamma_l (S_A^{ll} + S_C^{ll}) - (\sigma + 2\mu) S_T^{ll} - \beta_1^l S_T^{ll}.$$

We also have that $T_S^{rq} = S_T^{qr}$.

For susceptible individuals not on PrEP with a steady susceptible partner on PrEP we have

$$\frac{dS_{SP}^{hh}}{dt} = \rho SP_0^h S_0^h + \xi S_S^{hh} - (\xi + \sigma + 2\mu) S_{SP}^{hh} - (\beta_1^h + \zeta \beta_1^h) S_{SP}^{hh},$$
$$\frac{dS_{SP}^{lh}}{dt} = \rho SP_0^h S_0^l + \xi S_S^{lh} - (\sigma + 2\mu) S_{SP}^{lh} - (\beta_1^l + \zeta \beta_1^h) S_{SP}^{lh}.$$

Note that $SP_S^{hh} = S_{SP}^{hh}$ and $SP_S^{hl} = S_{SP}^{lh}$.

For susceptible individuals not on PrEP in a partnership with an acute infectious individual on PrEP

$$\frac{dS_{AP}^{hh}}{dt} = \rho S_0^h AP_0^h + \zeta \beta_1^h S_{SP}^{hh} - (\xi + \lambda p_A + \delta_a + \gamma_P + \sigma + 2\mu) S_{AP}^{hh} - \beta_1^h S_{AP}^{hh},$$
$$\frac{dS_{AP}^{lh}}{dt} = \rho S_0^l AP_0^h + \zeta \beta_1^h S_{SP}^{lh} - (\lambda p_A + \delta_a + \gamma_P + \sigma + 2\mu) S_{AP}^{lh} - \beta_1^l S_{AP}^{lh}.$$

Note that $AP_S^{hh} = S_{AP}^{hh}$ and $AP_S^{hl} = S_{AP}^{lh}$.

For susceptible individuals not on PrEP in a partnership with a chronic infectious individual on PrEP

$$\frac{dS_{CP}^{hh}}{dt} = \rho S_0^h CP_0^h + \delta_a S_{AP}^{hh} - (\xi + \lambda p_C + \gamma_P + \sigma + 2\mu) S_{CP}^{hh} - \beta_1^h S_{CP}^{hh}$$
$$\frac{dS_{CP}^{lh}}{dt} = \rho S_0^l CP_0^h + \delta_a S_{AP}^{lh} - (\lambda p_C + \gamma_P + \sigma + 2\mu) S_{CP}^{lh} - \beta_1^l S_{CP}^{lh}.$$

For peer review only - http://bmjopen.bmj.com/site/about/guidelines.xhtml

For peer review only

Note that $CP_S^{hh} = S_{CP}^{hh}$ and $CP_S^{hl} = S_{CP}^{lh}$.

S4.2.2 Acute infectious individuals not on PrEP

For acute infectious individuals not on PrEP in a partnership with another acute infectious not on PrEP we have

$$\frac{dA_A^{hh}}{dt} = \rho\left(A_0^h\right)^2 - (\sigma + 2\mu + 2\gamma_h + 2\delta_a)A_A^{hh} + 2\left(\lambda p_A + \beta_1^h\right)S_A^{hh},$$
$$\frac{dA_A^{hl}}{dt} = \rho A_0^h A_0^l - (\sigma + 2\mu + \gamma_h + \gamma_l + 2\delta_a)A_A^{hl} + (\lambda p_A + \beta_1^h)S_A^{hl} + (\lambda p_A + \beta_1^l)A_S^{hl},$$
$$\frac{dA_A^{ll}}{dt} = \rho\left(A_0^l\right)^2 - (\sigma + 2\mu + 2\gamma_l + 2\delta_a)A_A^{ll} + 2\left(\lambda p_A + \beta_1^l\right)S_A^{ll}.$$

where additionally $A_A^{lh} = A_A^{hl}$.

For acute infectious individuals in a partnership with a chronic infectious individual

$$\frac{dA_C^{hh}}{dt} = \rho A_0^h C_0^h + \delta_a A_A^{hh} - (\sigma + 2\mu + 2\gamma_h + \delta_a)A_C^{hh} + \left(\lambda p_C + \beta_1^h\right)S_C^{hh},$$
$$\frac{dA_C^{hl}}{dt} = \rho A_0^h C_0^l + \delta_a A_A^{hl} - (\sigma + 2\mu + \gamma_h + \gamma_l + \delta_a)A_C^{hl} + \left(\lambda p_C + \beta_1^h\right)S_C^{hl},$$
$$\frac{dA_C^{lh}}{dt} = \rho A_0^l C_0^h + \delta_a A_A^{lh} - (\sigma + 2\mu + \gamma_h + \gamma_l + \delta_a)A_C^{lh} + \left(\lambda p_C + \beta_1^l\right)S_C^{lh},$$
$$\frac{dA_C^{ll}}{dt} = \rho A_0^l C_0^l + \delta_a A_A^{ll} - (\sigma + 2\mu + 2\gamma_l + \delta_a)A_C^{ll} + \left(\lambda p_C + \beta_1^l\right)S_C^{ll}.$$

Note that $C_A^{rq} = A_C^{qr}$.

For acute infectious individuals in a partnership with an individual on ART-treatment

$$\frac{dA_T^{hh}}{dt} = \rho A_0^h T_0^h + \gamma_h(A_C^{hh} + A_A^{hh}) + \gamma_P(A_{CP}^{hh} + A_{AP}^{hh}) - (\gamma_h + \sigma + 2\mu + \delta_a)A_T^{hh} + \beta_1^h S_T^{hh}$$
$$\frac{dA_T^{hl}}{dt} = \rho A_0^h T_0^l + \gamma_l(A_C^{hl} + A_A^{hl}) - (\gamma_h + \sigma + 2\mu + \delta_a)A_T^{hl} + \beta_1^h S_T^{hl},$$
$$\frac{dA_T^{lh}}{dt} = \rho A_0^l T_0^h + \gamma_h(A_C^{lh} + A_A^{lh}) + \gamma_P(A_{CP}^{lh} + A_{AP}^{lh}) - (\gamma_l + \sigma + 2\mu + \delta_a)A_T^{lh} + \beta_1^l S_T^{lh},$$
$$\frac{dA_T^{ll}}{dt} = \rho A_0^l T_0^l + \gamma_l(A_C^{ll} + A_A^{ll}) - (\gamma_l + \sigma + 2\mu + \delta_a)A_T^{ll} + \beta_1^l S_T^{ll}.$$

Also, $T_A^{rq} = A_T^{qr}$.

For acute infectious individuals not on PrEP in a partnership with a susceptible individual on PrEP

$$\frac{dA_{SP}^{hh}}{dt} = \rho A_0^h SP_0^h + \xi A_S^{hh} + \beta_1^h S_{SP}^{hh} - (\zeta\lambda p_A + \delta_a + \gamma_h + \sigma + 2\mu)A_{SP}^{hh} - \zeta\beta_1^h A_{SP}^{hh},$$
$$\frac{dA_{SP}^{lh}}{dt} = \rho A_0^l SP_0^h + \xi A_S^{lh} + \beta_1^l S_{SP}^{lh} - (\zeta\lambda p_A + \delta_a + \gamma_l + \sigma + 2\mu)A_{SP}^{lh} - \zeta\beta_1^h A_{SP}^{lh}.$$

Note that $SP_A^{hh} = A_{SP}^{hh}$ and $SP_A^{hl} = A_{SP}^{lh}$.

For acute infectious individuals not on PrEP in a partnership with an acute infectious individual on PrEP

$$\frac{dA_{AP}^{hh}}{dt} = \rho A_0^h AP_0^h - (\sigma + 2\mu + \gamma_h + \gamma_P + 2\delta_a)A_{AP}^{hh} + (\lambda p_A + \beta_1^h)S_{AP}^{hh} + (\zeta\lambda p_A + \zeta\beta_1^h)A_{SP}^{hh},$$
$$\frac{dA_{AP}^{lh}}{dt} = \rho A_0^l AP_0^h - (\sigma + 2\mu + \gamma_l + \gamma_P + 2\delta_a)A_{AP}^{lh} + (\lambda p_A + \beta_1^l)S_{AP}^{lh} + (\zeta\lambda p_A + \zeta\beta_1^h)A_{SP}^{lh}.$$

For peer review only - http://bmjopen.bmj.com/site/about/guidelines.xhtml

Note that $AP_A^{hh} = A_{AP}^{hh}$ and $AP_A^{hl} = A_{AP}^{lh}$.

For acute infectious individuals not on PrEP in a partnership with a chronic infectious individual on PrEP

$$\frac{dA_{CP}^{hh}}{dt} = \rho A_0^h CP_0^h + \delta_a A_{AP}^{hh} - (\sigma + 2\mu + \gamma_h + \gamma_P + \delta_a)A_{CP}^{hh} + (\lambda p_C + \beta_1^h)S_{CP}^{hh},$$
$$\frac{dA_{CP}^{lh}}{dt} = \rho A_0^l CP_0^h + \delta_a A_{AP}^{lh} - (\sigma + 2\mu + \gamma_l + \gamma_P + \delta_a)A_{CP}^{lh} + (\lambda p_C + \beta_1^l)S_{CP}^{lh}.$$

Note that $CP_A^{hh} = A_{CP}^{hh}$ and $CP_A^{hl} = A_{CP}^{lh}$.

S4.2.3 Chronic infectious not on PrEP

For an individual with a chronic infection not on PrEP in a steady partnership with another individual with a chronic infection

$$\frac{dC_C^{hh}}{dt} = \rho \left(C_0^h\right)^2 + \delta_a(A_C^{hh} + C_A^{hh}) - (\sigma + 2\mu + 2\gamma_h)C_C^{hh},$$
$$\frac{dC_C^{hl}}{dt} = \rho C_0^h C_0^l + \delta_a(A_C^{hl} + C_A^{hl}) - (\sigma + 2\mu + \gamma_h + \gamma_l)C_C^{hl}$$
$$\frac{dC_C^{ll}}{dt} = \rho \left(C_0^l\right)^2 + \delta_a(A_C^{ll} + C_A^{ll}) - (\sigma + 2\mu + 2\gamma_l)C_C^{ll}.$$

where $C_C^{lh} = C_C^{hl}$.

For an individual with a chronic infection not on PrEP in a steady partnership with an individual under ART-treatment

$$\frac{dC_T^{hh}}{dt} = \rho C_0^h T_0^h + \delta_a A_T^{hh} + \gamma_h(C_A^{hh} + C_C^{hh}) + \gamma_P(C_{AP}^{hh} + C_{CP}^{hh}) - (\sigma + 2\mu + \gamma_h)C_T^{hh},$$
$$\frac{dC_T^{hl}}{dt} = \rho C_0^h T_0^l + \delta_a A_T^{hl} + \gamma_l(C_A^{hl} + C_C^{hl}) - (\sigma + 2\mu + \gamma_h)C_T^{hl},$$
$$\frac{dC_T^{lh}}{dt} = \rho C_0^l T_0^h + \delta_a A_T^{lh} + \gamma_h(C_A^{lh} + C_C^{lh}) + \gamma_P(C_{AP}^{lh} + C_{CP}^{lh}) - (\sigma + 2\mu + \gamma_l)C_T^{lh},$$
$$\frac{dC_T^{ll}}{dt} = \rho C_0^l T_0^l + \delta_a A_T^{ll} + \gamma_l(C_A^{ll} + C_C^{ll}) - (\sigma + 2\mu + \gamma_l)C_T^{ll}.$$

where $T_C^{rq} = C_T^{qr}$.

For an individual with a chronic infection not on PrEP in a partnership with a susceptible individual on PrEP

$$\frac{dC_{SP}^{hh}}{dt} = \rho C_0^h SP_0^h + \xi C_S^{hh} + \delta_a A_{SP}^{hh} - (\zeta\lambda p_C + \gamma_h + \sigma + 2\mu)C_{SP}^{hh} - \zeta\beta_1^h C_{SP}^{hh}$$
$$\frac{dC_{SP}^{lh}}{dt} = \rho C_0^l SP_0^h + \xi C_S^{lh} + \delta_a A_{SP}^{lh} - (\zeta\lambda p_C + \gamma_l + \sigma + 2\mu)C_{SP}^{lh} - \zeta\beta_1^h C_{SP}^{lh}.$$

And additionally $SP_C^{hh} = C_{SP}^{hh}$ and $SP_C^{hl} = C_{SP}^{lh}$.

For an individual with a chronic infection not on PrEP in a partnership with an acute infectious individual on PrEP

$$\frac{dC_{AP}^{hh}}{dt} = \rho C_0^h AP_0^h + \delta_a A_{AP}^{hh} - (\sigma + 2\mu + \gamma_h + \gamma_P + \delta_a)C_{AP}^{hh} + (\zeta\lambda p_C + \zeta\beta_1^h)C_{SP}^{hh},$$
$$\frac{dC_{AP}^{lh}}{dt} = \rho C_0^l AP_0^h + \delta_a A_{AP}^{lh} - (\sigma + 2\mu + \gamma_l + \gamma_P + \delta_a)C_{AP}^{lh} + (\zeta\lambda p_C + \zeta\beta_1^h)C_{SP}^{lh}.$$

Note that $AP_C^{hh} = C_{AP}^{hh}$ and $AP_C^{hl} = C_{AP}^{lh}$.

For peer review only - http://bmjopen.bmj.com/site/about/guidelines.xhtml
For peer review only

For an individual with a chronic infection not on PrEP in a partnership with a chronic infectious individual on PrEP

$$\frac{dC^{hh}_{CP}}{dt} = \rho C^h_0 CP^h_0 + \delta_a(A^{hh}_{CP} + C^{hh}_{AP}) - (\sigma + 2\mu + \gamma_h + \gamma_P)C^{hh}_{CP},$$
$$\frac{dC^{lh}_{CP}}{dt} = \rho C^l_0 CP^h_0 + \delta_a(A^{lh}_{CP} + C^{lh}_{AP}) - (\sigma + 2\mu + \gamma_l + \gamma_P)C^{lh}_{CP}.$$

where $CP^{hh}_C = C^{hh}_{CP}$ and $CP^{hl}_C = C^{lh}_{CP}$.

S4.2.4 Treated individual

For an individual under ART-treatment in a steady partnership with another individual under ART-treatment we have

$$\frac{dT^{hh}_T}{dt} = \rho(T^h_0)^2 + \gamma_h(A^{hh}_T + C^{hh}_T + T^{hh}_A + T^{hh}_C) + \gamma_P(AP^{hh}_T + CP^{hh}_T + T^{hh}_{AP} + T^{hh}_{CP}) - (\sigma + 2\mu)T^{hh}_T,$$
$$\frac{dT^{hl}_T}{dt} = \rho T^h_0 T^l_0 + \gamma_h(A^{hl}_T + C^{hl}_T) + \gamma_l(T^{hl}_A + T^{hl}_C) + \gamma_P(AP^{hl}_T + CP^{hl}_T) - (\sigma + 2\mu)T^{hl}_T$$
$$\frac{dT^{ll}_T}{dt} = \rho(T^l_0)^2 + \gamma_l(A^{ll}_T + C^{ll}_T) + \gamma_l(T^{ll}_A + T^{ll}_C) - (\sigma + 2\mu)T^{ll}_T.$$

where $T^{lh}_T = T^{hl}_T$.

For treated individuals in a partnership with a susceptible individual on PrEP

$$\frac{dT^{hh}_{SP}}{dt} = \rho T^h_0 SP^h_0 + \xi T^{hh}_S + \gamma_h(A^{hh}_{SP} + C^{hh}_{SP}) + \gamma_P(AP^{hh}_{SP} + CP^{hh}_{SP}) - (\sigma + 2\mu)T^{hh}_{SP} - \zeta\beta^h_1 T^{hh}_{SP},$$
$$\frac{dT^{lh}_{SP}}{dt} = \rho T^l_0 SP^h_0 + \xi T^{lh}_S + \gamma_l(A^{lh}_{SP} + C^{lh}_{SP}) - (\sigma + 2\mu)T^{lh}_{SP} - \zeta\beta^h_1 T^{lh}_{SP}.$$

We also have that $SP^{hh}_T = T^{hh}_{SP}$ and $SP^{hl}_T = T^{lh}_{SP}$.

For treated individuals in a partnership with an acute infectious individual on PrEP

$$\frac{dT^{hh}_{AP}}{dt} = \rho T^h_0 AP^h_0 + \gamma_h(C^{hh}_{AP} + A^{hh}_{AP}) + \gamma_P(AP^{hh}_{AP} + CP^{hh}_{AP}) - (\gamma_P + \sigma + 2\mu + \delta_a)T^{hh}_{AP} + \zeta\beta^h_1 T^{hh}_{SP}$$
$$\frac{dT^{lh}_{AP}}{dt} = \rho T^l_0 AP^h_0 + \gamma_l(C^{lh}_{AP} + A^{lh}_{AP}) - (\gamma_P + \sigma + 2\mu + \delta_a)T^{lh}_{AP} + \zeta\beta^h_1 T^{lh}_{SP}.$$

Also, $AP^{hh}_T = T^{hh}_{AP}$ and $AP^{hl}_T = T^{lh}_{AP}$.

For treated individuals in a partnership with a chronic infectious individual on PrEP

$$\frac{dT^{hh}_{CP}}{dt} = \rho T^h_0 CP^h_0 + \delta_a T^{hh}_{AP} + \gamma_h(A^{hh}_{CP} + C^{hh}_{CP}) + \gamma_P(AP^{hh}_{CP} + CP^{hh}_{CP}) - (\sigma + 2\mu + \gamma_P)T^{hh}_{CP},$$
$$\frac{dT^{lh}_{CP}}{dt} = \rho T^l_0 CP^h_0 + \delta_a T^{lh}_{AP} + \gamma_l(A^{lh}_{CP} + C^{lh}_{CP}) - (\sigma + 2\mu + \gamma_P)T^{lh}_{CP}.$$

where $CP^{hh}_T = T^{hh}_{CP}$ and $CP^{hl}_T = T^{lh}_{CP}$.

S4.2.5 Individuals on PrEP

For susceptible individuals on PrEP with a steady partner on PrEP we have

$$\frac{dSP^{hh}_{SP}}{dt} = \rho(SP^h_0)^2 + 2\xi SP^{hh}_S - (\sigma + 2\mu)SP^{hh}_{SP} - 2\zeta\beta^h_1 SP^{hh}_{SP},$$
$$\frac{dSP^{hh}_{AP}}{dt} = \rho SP^h_0 AP^h_0 + \xi S^{hh}_{AP} + \zeta\beta^h_1 SP^{hh}_{SP} - (\zeta\lambda p_A + \delta_a + \gamma_P + \sigma + 2\mu)SP^{hh}_{AP} - \zeta\beta^h_1 SP^{hh}_{AP},$$
$$\frac{dSP^{hh}_{CP}}{dt} = \rho SP^h_0 CP^h_0 + \xi S^{hh}_{CP} + \delta_a SP^{hh}_{AP} - (\zeta\lambda p_C + \gamma_P + \sigma + 2\mu)SP^{hh}_{CP} - \zeta\beta^h_1 SP^{hh}_{CP}.$$

For peer review only - http://bmjopen.bmj.com/site/about/guidelines.xhtml

Note that $AP^{hh}_{SP} = SP^{hh}_{AP}$ and that $CP^{hh}_{SP} = SP^{hh}_{CP}$.

For acute infectious individuals on PrEP in a steady partnership with an individual on PrEP

$$\frac{dAP^{hh}_{AP}}{dt} = \rho(AP^h_0)^2 - (\sigma + 2\mu + 2\gamma_P + 2\delta_a)AP^{hh}_{AP} + 2(\zeta\lambda p_A + \zeta\beta^h_1)AP^{hh}_{SP},$$
$$\frac{dAP^{hh}_{CP}}{dt} = \rho AP^h_0 CP^h_0 + \delta_a AP^{hh}_{AP} - (\sigma + 2\mu + 2\gamma_P + \delta_a)AP^{hh}_{CP} + (\zeta\lambda p_C + \zeta\beta^h_1)SP^{hh}_{CP}.$$

Note that $CP^{hh}_{AP} = AP^{hh}_{CP}$.

And very much finally, for an individual with a chronic infection on PrEP in a partnership with a chronic infectious individual on PrEP we have

$$\frac{dCP^{hh}_{CP}}{dt} = \rho(CP^h_0)^2 + \delta_a(AP^{hh}_{CP} + CP^{hh}_{AP}) - (\sigma + 2\mu + 2\gamma_P)CP^{hh}_{CP}.$$

For peer review only

S5 Additional results

We will in this Section go through some additional results mentioned in the main manuscript.

S5.1 Not distinguishing individuals according to activity degree

Here we examine what happens if we do not divide the population according to active-degree but assume that everyone behaves in the same way regarding the number of casual sex partners and regarding the rate to ART-treatment. For the case when no one yet is on PrEP, we find that $R_0 = 1$ when the mean time to successful ART-treatment is 3.28 years. A prevalence of 5% is obtained for a mean time to ART-treatment of 3.57 years. In Figure S3 we show the effect of introducing PrEP in this model without high-actives and low-actives. We see that the PrEP coverage in such a population would need to exceed 5% to reach a prevalence close to 0 (in contrast to 3.5% as in the model where we have two activity degrees).

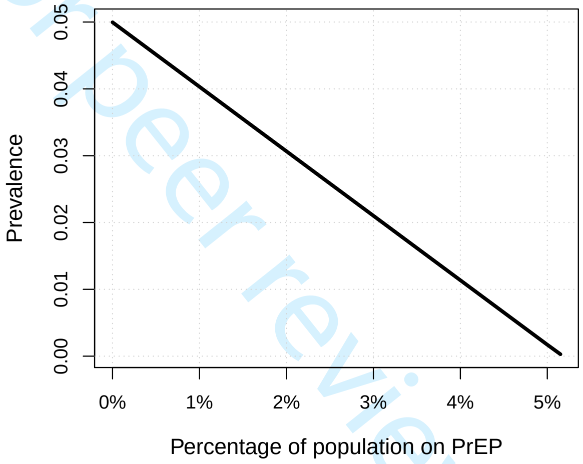

Figure S3: Effect of introducing PrEP in a population that is not separated according to activity degree, but where everyone is assumed to behave the same with regards of finding new casual sex partners.

S5.2 Not including the different infectious stages acute and chronic

In Figure 2b) in the main text, we saw that the reduction in susceptibility due to PrEP had a larger effect in reducing the prevalence than the increased testing rate of those on PrEP. The transmission probability of HIV is much higher in the acute infectious phase, the first 3 months following infection, than in the chronic phase. The reason for the lesser effect of an increased testing rate could be that it misses a large proportion of the acute stage.

To help verify that this is the case, we modified the model to not make a distinction between the acute and chronic stage; to only include one transmission probability during the whole infectious lifetime of an infected individual. We calibrate this transmission probability so that when no one is on PrEP, and the mean time to successful ART-treatment is 1.77 years for high-actives, the prevalence is equal to 5%. This is done to match the set-up of the analysis in Figure 2b). The transmission probability is then 0.0208 for the whole infectious time, instead of 0.1301 for the acute stage and 0.0098 for the chronic stage.

In Figure S4 it is seen that when only one infectious stage is included, the increased testing and diagnosis rate has as equally big impact on the reduction of the prevalence as the reduced susceptibility of PrEP. This implies that the lesser effect of the increased testing rate, that we found in Figure 2b) in the main manuscript, can be assigned to it missing the 3 month long acute stage. Because, when we in this analysis distributed the increased transmission probability of the acute stage over an infected individual's lifetime,

For peer review only - http://bmjopen.bmj.com/site/about/guidelines.xhtml

an increased testing rate got a bigger effect in reducing the prevalence than when we separated the infectious stages.

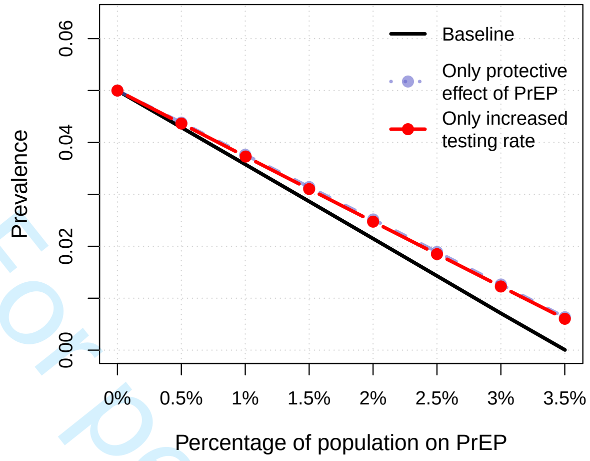

Figure S4: Same set up as corresponding figure in main text, Figure 2b), but with no distinction between the acute and chronic stage but only one infectious stage with one transmission probability.

S5.3 Range of 95% CRI

In Figure 1b) in the main manuscript we show the 95% credibility intervals of the estimated prevalence for all three mixing assumptions. This was done for different mean times to ART-treatment to obtain the credibility bands as in Figure S5a. Since it can be somewhat hard to compare the widths of said intervals in Figure 1b) and Figure S5a, we additionally calculated the range of each credibility intervals and show the boxplot of these values in Figure S5b. From Figure S5b it is seen that using the complete assortativity assumption regarding activity-degree has most narrow 95% credibility intervals. Moreover, the fitted assortativity has more narrow credibility intervals than the proportionate mixing assumption.

S5.4 Effect of different PrEP effectiveness in reducing susceptibility

In the introduction of the main manuscript we gave the estimated reduction of susceptibility of PrEP: 86% with a 95% confidence interval of 40 - 98% [S5]. To investigate the effect of this variability, we analysed the effect of introducing PrEP among high-active individuals with a 40% PrEP effectiveness, and then with a 98% PrEP effectiveness. The results can be seen in Figure S6. We see that, using the much less effective value of 40% instead of 86%, to reach an endemic prevalence close to 0 increases the needed PrEP coverage from 3.5% of the population (10.4% of all high-actives) to 4.4% of the population (13.1% of all high-actives).

S5.5 Effect of giving PrEP to low-actives instead of high-actives

In our analysis we mainly focus on the effect of high-active individuals accepting PrEP. If we instead want to determine the effect of targeting low-actives for PrEP, we could just reverse which activity-group is allowed to start taking PrEP. Here, we also show the results for when only low-actives are offered PrEP. As we can see from Figure S7, a much higher coverage (35%) is needed to reach the same long-term prevalence reduction compared to if high-actives were offered PrEP (3.5%) (Figure 2a in the main manuscript).

For peer review only - http://bmjopen.bmj.com/site/about/guidelines.xhtml

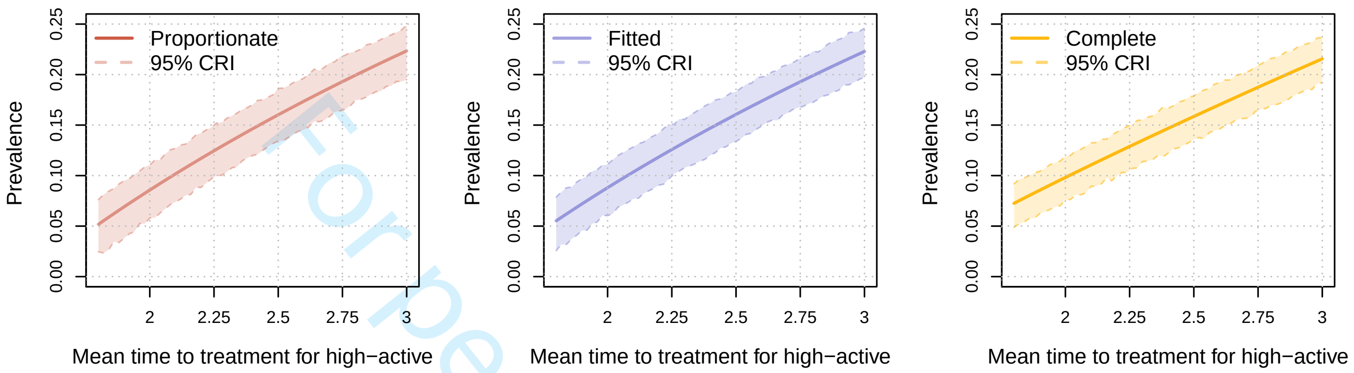

(a)

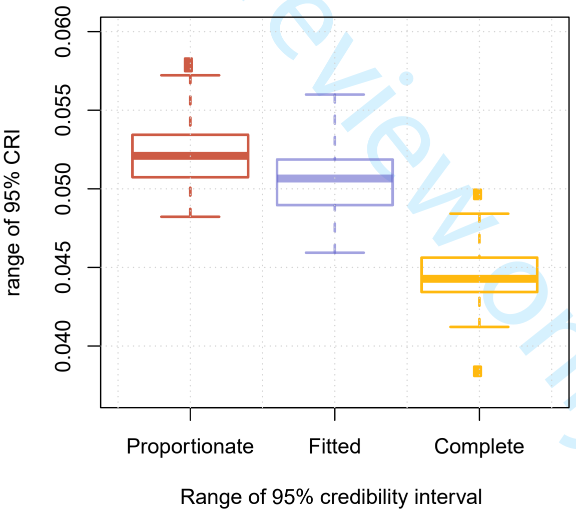

(b)

Figure S5: The width of the estimated 95% credibility intervals. (a) depicts the credibility bands from which we calculated the range of the credibility intervals, summarised as boxplots in (b).

For peer review only - http://bmjopen.bmj.com/site/about/guidelines.xhtml

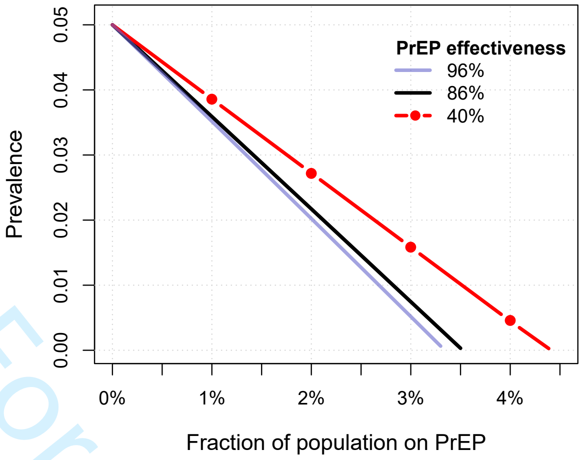

Figure S6: Endemic prevalence for different PrEP coverages and effectiveness.

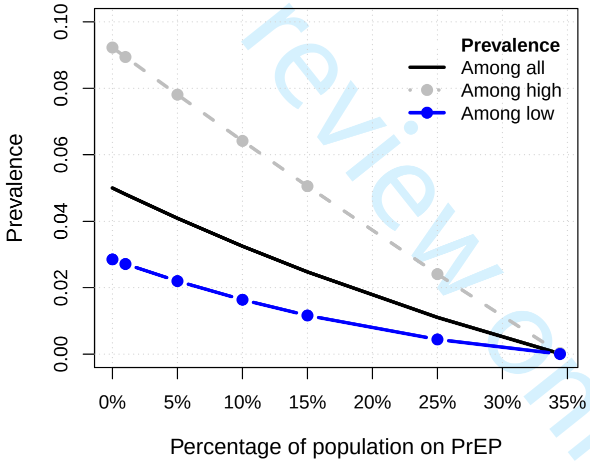

Figure S7: Effect of giving PrEP to low-actives instead of high-actives.

For peer review only - http://bmjopen.bmj.com/site/about/guidelines.xhtml

S5.6 PrEP coverage for alternative transmission probabilities

The transmission probabilities for unprotected receptive anal intercourse (URAI) during the acute and chronic stage are taken from the literature [S6] (0.1835 and 0.0138, respectively). To get the transmission probabilities during unprotected insertive anal intercourse (UIAI), we use an estimate of the relationship between the transmission probability between URAI and UIAI. The transmission probability for URAI is 2.39 times larger than the transmission probability for UIAI [S7]. Assuming equally many insertive as receptive acts, the transmission probability during the acute stage was set to

$$p_A = 0.1835 \times 0.5 + 0.1835 \times 0.5/2.39 = 0.1301,$$

and during the chronic stage

$$p_C = 0.0138 \times 0.5 + 0.0138 \times 0.5/2.39 = 0.0098.$$

We now want to study how robust our conclusion concerning PrEP coverage, to achieve an endemic prevalence close to 0%, is. We do this by altering the two transmission probabilities by setting them to $50\% - 150\%$ of their estimated values. For example: with 50% of the transmission probabilities, we have that $p_A = 0.0651$ and $p_C = 0.0049$; with 150% of the transmission probabilities, we have that $p_A = 0.1952$ and $p_C = 0.0147$. With given transmission probabilities p_A and p_C, we find the mean time to ART-treatment corresponding to a prevalence of 5%. The given mean time to ART-treatment are for high-actives, for low-actives it is 2.35 times larger. With the different set-ups generating a prevalence of 5%, PrEP is introduced to high-active individuals. In Table S7 we show the different set-ups and the PrEP coverage needed to get an endemic prevalence close to 0. Additionally, we alter the two transmission probabilities one at a time in Table S8 and Table S9. In Table S8 we vary p_A but let p_C stay fixed at 0.0098. In Table S9 we vary p_C but let p_A stay fixed at 0.1301. We conclude by noting that the results are almost invariant to which set-up is used.

Table S7: **Robustness of the PrEP coverage to obtain a long-term prevalence close to 0: alteration of p_A and p_C.** Assuming no one is on PrEP, we first find other combinations than the one used in the main manuscript of the transmission probabilities and mean time to ART-treatment for high-actives that generates a prevalence of 5%. With these different scenarios that generates a prevalence of 5%, we then study the needed PrEP coverage to obtain a long-term prevalence close to 0.

	**% of transmission probabilities $p_A = 0.1301$ and $p_C = 0.0098$**										
	50%	60%	70%	80%	90%	100%	110%	120%	130%	140%	150%
Time to ART-treatment	5.38	4.10	3.23	2.60	2.13	1.77	1.48	1.25	1.07	0.92	0.80
PrEP coverage for 0 prevalence	3.42%	3.43%	3.44%	3.47%	3.49%	3.52%	3.55%	3.58%	3.61%	3.65%	3.68%

Table S8: **Robustness of the PrEP coverage to obtain a long-term prevalence close to 0: alteration of p_A.** Same procedure as in Table S7, but only the transmission probability in the acute stage is altered. The transmission probability in the chronic stage is remained fixed at $p_C = 0.0098$.

	**% of transmission probabilities $p_A = 0.1301$.**										
	50%	60%	70%	80%	90%	100%	110%	120%	130%	140%	150%
Time to ART-treatment	2.76	2.55	2.35	2.15	1.96	1.77	1.59	1.42	1.26	1.12	0.98
PrEP coverage for 0 prevalence	3.44%	3.45%	3.46%	3.48%	3.50%	3.52%	3.55%	3.58%	3.61%	3.64%	3.67%

For peer review only

For peer review only - http://bmjopen.bmj.com/site/about/guidelines.xhtml

Table S9: **Robustness of the PrEP coverage to obtain a long-term prevalence close to 0: alteration of p_C.** Same procedure as in Table S7, but only the transmission probability in the chronic stage is altered. The transmission probability in the acute stage is remained fixed at $p_A = 0.1301$.

	**% of transmission probabilities $p_C = 0.0098$.**										
	50%	60%	70%	80%	90%	100%	110%	120%	130%	140%	150%
Time to ART-treatment	3.14	2.68	2.36	2.11	1.92	1.77	1.64	1.54	1.45	1.37	1.30
PrEP coverage for 0 prevalence	3.5%	3.5%	3.51%	3.51%	3.52%	3.52%	3.53%	3.53%	3.53%	3.54%	3.54%

S5.7 Short-term effect of different PrEP strategies

In our model, we assume that a sexually high-active start using PrEP at rate ξ. We then determine the lowest possible rate ξ that yields an equilibrium prevalence of 0% and calculate which PrEP coverage this corresponds to. The lowest PrEP coverage that *eventually* results in a 0% HIV prevalence is 3.5% of the population. This 'eventually' is a very long time in the future—it would take centuries. If no new HIV cases would occur, it would still take many years before the prevalence reaches 0%; the HIV prevalence would not reach 0% until the last person with HIV dies. However, HIV will effectively disappear when no new infections occur. Remember that, in our model we assume that diagnosis and the beginning of ART-treatment is the same as being uninfectious, and consequently, only individuals with undiagnosed HIV can transmit the infection. Hence, we will here study not only the prevalence but also the percentage undiagnosed HIV cases for different PrEP initiation rates, ξ.

In what follows, we will look at different rates ξ, where all rates result in an HIV prevalence of 0% in the equilibrium steady state. The lowest ξ we look at will therefore corresponds to an equilibrium PrEP coverage of 3.5% of the population ($\approx$ 10% of high-actives). As a starting point, before any high-active accepts PrEP, the prevalence is set to 5% and the percentage undiagnosed HIV cases to 0.21% (the model with $\xi = 0$ calibrated to data). In the left panel of Figure S8, we show the HIV prevalence (%) at different PrEP initiation rates, ξ, for 50 years after the beginning of a PrEP implementation programme. In the middle panel of Figure S8, we show the percentage of individuals that are infectious and undiagnosed. In the right panel of S8, we show the corresponding percentages of the population that are on PrEP for 50 years after the beginning of the PrEP programme. For the lowest rate ξ that results in an equilibrium HIV prevalence of 0%, we see that after 50 years the PrEP coverage has only had time to reach 2%, but the percentage undiagnosed has more than halved, and the prevalence has dropped from 5% to almost 4%. This can be compared to the scenario with a ξ that results in an equilibrium PrEP coverage of 11%. Then the PrEP coverage has reached a bit over 7% after 50 years, and the percentage undiagnosed HIV cases is only 1/20 of its value before the initiation of a PrEP programme (from 0.21% to 0.01%).

In Figure S9 we study, in more detail, the effects of different PrEP scenarios 10 and 20 years after their initiation. We look at both the prevalence and the percentage undiagnosed HIV cases. After 10 years, if the PrEP coverage has reached 5% (15% of all high-actives), the percentage undiagnosed HIV cases is reduced from 0.21% to 0.14%. Looking at 20 years after a PrEP programmes initiation and where the PrEP coverage has reached 5%, the percentage undiagnosed HIV cases is reduced to the low level of 0.04%. If the PrEP coverage on the other hand has reached almost all high-actives after 10 years, that is 30% of the population, then the percentage undiagnosed HIV cases is reduced to 0.03%. The same percentage of coverage after 20 years yields a percentage of 0.004% infectious and undiagnosed HIV cases; that is, almost no new HIV infections occur.

For peer review only - http://bmjopen.bmj.com/site/about/guidelines.xhtml

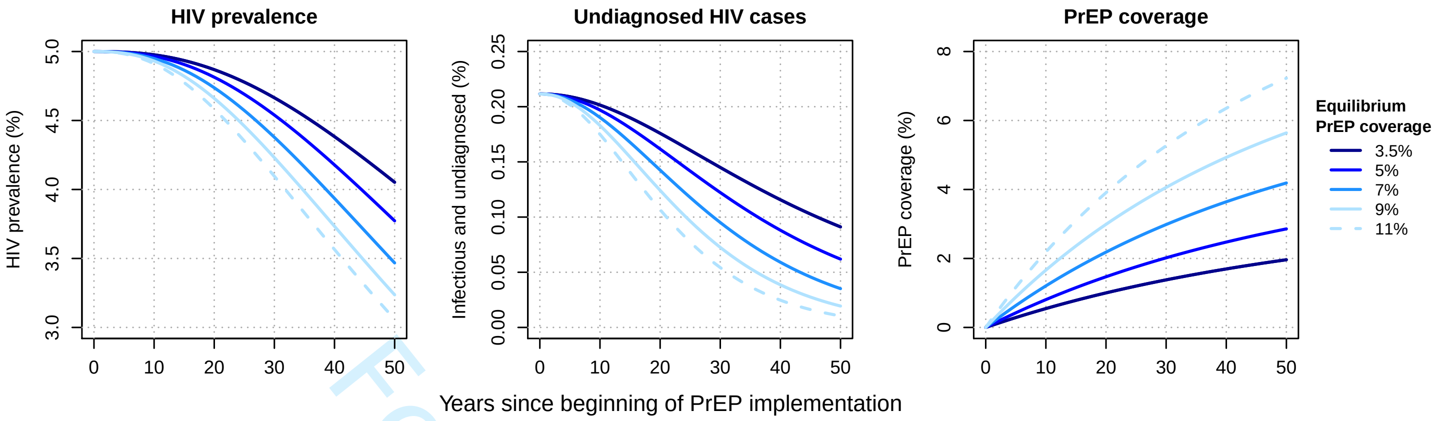

Figure S8: Effect of different PrEP scenarios on the HIV prevalence and new HIV cases (infectious and undiagnosed).

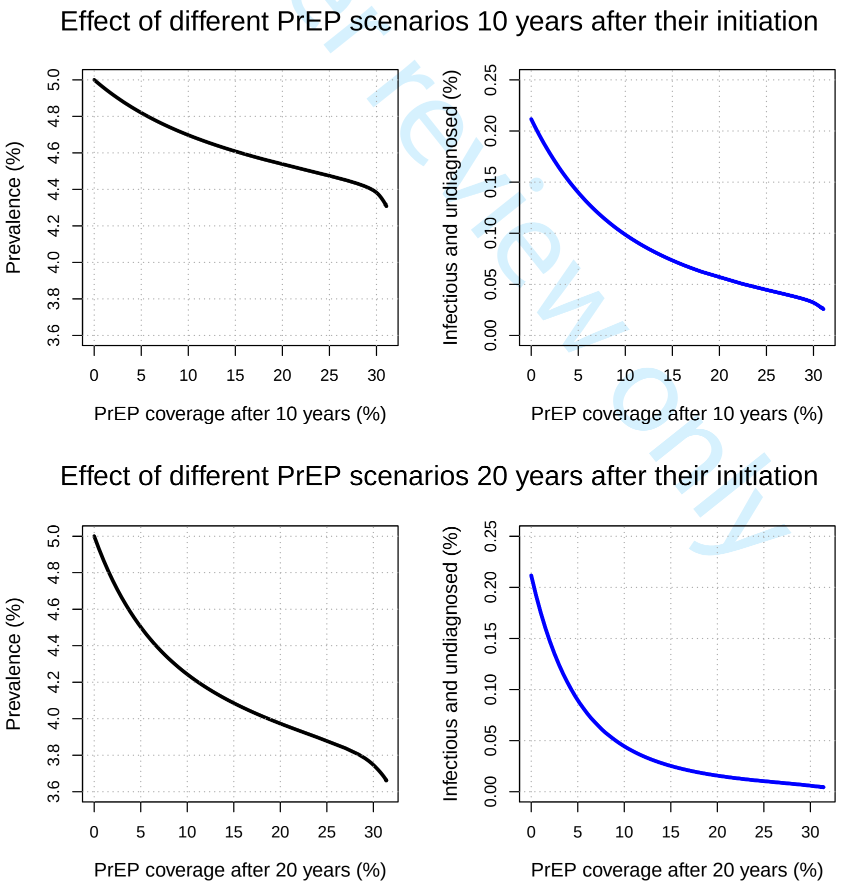

Figure S9: Effect of different PrEP scenarios 10 and 20 years after their initiation.

For peer review only - http://bmjopen.bmj.com/site/about/guidelines.xhtml

S5.8 Allowing individuals to change activity-group

The conclusions of the PrEP coverage needed to eventually eliminate HIV from the community (an equilibrium HIV prevalence of 0%) does not vary much if participants are allowed to switch activity-group.

We further extended our main model to ascertain the effect of allowing high-active individuals to become low-active and allowing low-active individuals to become high-active. The fraction high-active and the fraction low-active in the population were held constant during this analysis. This was achieved by introducing a parameter ν governing the switching, then letting $\pi_h \nu$ be the rate for one low-active to change to being high-active and letting $\pi_l \nu$ be the rate for one high-active to change to being low-active. The number of low-active MSM in the population is $n\pi_l$ and the number of high-active MSM is $n\pi_h$. Therefore, the total rate for low-actives to switch will be $n\pi_l \pi_h \nu$ and the total rate for high-actives to switch will be $n\pi_h \pi_l \nu$. Hence, the fractions being high-active and low-active will fluctuate around the values π_h and π_l. If a high-active on PrEP switch to low-active we additionally assume that this individual stops taking PrEP.

We tested different switching rates $\nu = 0$, 0.01, 0.1, 0.2, 0.5, 0.75, 1. If, for example, $\nu = 0.1$ then one randomly chosen individual will switch on average every tenth year and if $\nu = 0.5$ then one randomly chosen individual will switch on average every second year. The case when $\nu = 0$ corresponds to no switching. For each scenario we calibrate the mean time until ART-treatment so that without anyone on PrEP the prevalence is 5%. As before, the given mean time to ART-treatment is for high-actives, for low-actives it is 2.35 times larger. The results can be seen in Table S10, from there we see that the PrEP coverage needed to eventually eliminate HIV from the community is not that different between the scenarios, it varies between $\approx 2.5\%$ to $\approx 3.5\%$ of the total population. However, when people on PrEP are allowed to become low-active and thereby stop taking PrEP a higher PrEP-initiation rate is needed to obtain a certain PrEP coverage.

In Table S11 we instead show the PrEP coverage needed when low-actives are targeted. We see the same tendency in which scenarios that easiest eliminate HIV as in Table S10. We also find the same kind of conclusion as in the main text—targeting high-actives for PrEP is much more effective than targeting low-actives.

Table S10: **HIV prevalence (%) for different switching scenarios and PrEP coverages.**

	$\nu = 0$	$\nu = 0.01$	$\nu = 0.1$	$\nu = 0.2$	$\nu = 0.5$	$\nu = 0.75$	$\nu = 1$
Time to ART	1.770	1.731	1.697	1.707	1.744	1.770	1.793
PrEP coverage							
1%	3.589	3.364	2.917	2.859	2.856	2.880	2.904
2%	2.172	1.718	0.836	0.716	0.715	0.761	0.806
2.5%	1.459	0.891	0.0002	0.0007	0.0007	0	0.0002
3%	0.745	0.067	0	0	0	0	0
3.5%	0	0	0	0	0	0	0

Table S11: **HIV prevalence (%) for different switching scenarios and PrEP coverages when low-actives are being targeted.**

	$\nu = 0$	$\nu = 0.01$	$\nu = 0.1$	$\nu = 0.2$	$\nu = 0.5$	$\nu = 0.75$	$\nu = 1$
Time to ART	1.770	1.731	1.697	1.707	1.744	1.770	1.793
PrEP coverage							
10%	3.249	3.076	2.562	2.351	2.020	1.852	1.726
20%	1.761	1.387	0.274	0.0002	0.0003	0	0
30%	0.503	0.0007	0	0	0	0	0
35%	0	0	0	0	0	0	0

For peer review only - http://bmjopen.bmj.com/site/about/guidelines.xhtml

References

[S1] Leung KY, Kretzschmar M, and Diekmann O. SI infection on a dynamic partnership network: characterization of R_0. *Journal of Mathematical Biology*, 71(1):1–56, 2015.

[S2] D. Hansson, K.Y. Leung, T. Britton, and S. Strömdahl. A dynamic network model to disentangle the roles of steady and casual partners for HIV transmission among MSM. *Epidemics*, 2019. ISSN 1755-4365. doi: https://doi.org/10.1016/j.epidem.2019.02.001. URL http://www.sciencedirect.com/science/article/pii/S1755436518300963.

[S3] Tom Britton. Stochastic epidemic models: A survey. *Mathematical Biosciences*, 225(1):24 – 35, 2010. ISSN 0025-5564. doi: https://doi.org/10.1016/j.mbs.2010.01.006. URL http://www.sciencedirect.com/science/article/pii/S0025556410000143.

[S4] Hollingsworth TD, Anderson RM., and Fraser C. HIV-1 transmission, by stage of infection. *The Journal of Infectious Diseases*, 198(5):687, 2008. doi: 10.1086/590501.

[S5] Molina JM, Capitant C, Spire B, et al. On-Demand Preexposure Prophylaxis in Men at High Risk for HIV-1 Infection. *New England Journal of Medicine*, 373(23):2237–2246, 2015. doi: 10.1056/NEJMoa1506273. PMID: 26624850.

[S6] Leynaert B, Downs AM, de Vincenzi I., et al. Heterosexual transmission of human immunodeficiency virus variability of infectivity throughout the course of infection. *American journal of epidemiology*, 148 (1):88–96, 1998.

[S7] Jin F, Jansson J, Law M, Prestage GP, Zablotska I, Imrie JCG, Kippax SC, Kaldor JM, Grulich AE, and Wilson DP. Per-contact probability of HIV transmission in homosexual men in Sydney in the era of HAART. *AIDS*, 24(6):907–913, 2010. doi: 10.1097/QAD.0b013e3283372d90.

For peer review only

For peer review only - http://bmjopen.bmj.com/site/about/guidelines.xhtml

STROBE Statement—Checklist of items that should be included in reports of ***cross-sectional studies***

	Item No	Recommendation	Reported on page #
**Title and abstract**	1	(*a*) Indicate the study's design with a commonly used term in the title or the abstract	1
		(*b*) Provide in the abstract an informative and balanced summary of what was done and what was found	2-3
**Introduction**			
Background/rationale	2	Explain the scientific background and rationale for the investigation being reported	4-5
Objectives	3	State specific objectives, including any prespecified hypotheses	5
**Methods**			
Study design	4	Present key elements of study design early in the paper	5-6
Setting	5	Describe the setting, locations, and relevant dates, including periods of recruitment, exposure, follow-up, and data collection	9
Participants	6	(*a*) Give the eligibility criteria, and the sources and methods of selection of participants	9
Variables	7	Clearly define all outcomes, exposures, predictors, potential confounders, and effect modifiers. Give diagnostic criteria, if applicable	Supplementary material S3
Data sources/ measurement	8*	For each variable of interest, give sources of data and details of methods of assessment (measurement). Describe comparability of assessment methods if there is more than one group	
Bias	9	Describe any efforts to address potential sources of bias	
Study size	10	Explain how the study size was arrived at	
Quantitative variables	11	Explain how quantitative variables were handled in the analyses. If applicable, describe which groupings were chosen and why	Supplementary material S3
Statistical methods	12	(*a*) Describe all statistical methods, including those used to control for confounding	
		(*b*) Describe any methods used to examine subgroups and interactions	
		(*c*) Explain how missing data were addressed	
		(*d*) If applicable, describe analytical methods taking account of sampling strategy	
		(*e*) Describe any sensitivity analyses	
**Results**			
Participants	13*	(a) Report numbers of individuals at each stage of study—eg numbers potentially eligible, examined for eligibility, confirmed eligible, included in the study, completing follow-up, and analysed	9
		(b) Give reasons for non-participation at each stage	
		(c) Consider use of a flow diagram	
Descriptive data	14*	(a) Give characteristics of study participants (eg demographic, clinical, social) and information on exposures and potential confounders	Separate paper
		(b) Indicate number of participants with missing data for each variable of interest	9
Outcome data	15*	Report numbers of outcome events or summary measures	11
Main results	16	(*a*) Give unadjusted estimates and, if applicable, confounder-adjusted	12-14

For peer review only

For peer review only - http://bmjopen.bmj.com/site/about/guidelines.xhtml

		estimates and their precision (eg, 95% confidence interval). Make clear which confounders were adjusted for and why they were included	
		(*b*) Report category boundaries when continuous variables were categorized	
		(*c*) If relevant, consider translating estimates of relative risk into absolute risk for a meaningful time period	
Other analyses	17	Report other analyses done—eg analyses of subgroups and interactions, and sensitivity analyses	Supplemental material S4
**Discussion**			
Key results	18	Summarise key results with reference to study objectives	15-16
Limitations	19	Discuss limitations of the study, taking into account sources of potential bias or imprecision. Discuss both direction and magnitude of any potential bias	17
Interpretation	20	Give a cautious overall interpretation of results considering objectives, limitations, multiplicity of analyses, results from similar studies, and other relevant evidence	16
Generalisability	21	Discuss the generalisability (external validity) of the study results	17
**Other information**			
Funding	22	Give the source of funding and the role of the funders for the present study and, if applicable, for the original study on which the present article is based	18

*Give information separately for exposed and unexposed groups.

Note: An Explanation and Elaboration article discusses each checklist item and gives methodological background and published examples of transparent reporting. The STROBE checklist is best used in conjunction with this article (freely available on the Web sites of PLoS Medicine at http://www.plosmedicine.org/, Annals of Internal Medicine at http://www.annals.org/, and Epidemiology at http://www.epidem.com/). Information on the STROBE Initiative is available at www.strobe-statement.org.

For peer review only

For peer review only - http://bmjopen.bmj.com/site/about/guidelines.xhtml
